# Supplementary material for: Substrate scope expansion of 4-phenol oxidases by rational enzyme selection and sequence-function relations
Source: Commun Chem. 2024 Jun 3;7:123. doi: 10.1038/s42004-024-01207-1 (PMC11148156; doi:10.1038/s42004-024-01207-1)
Supplement: Supplementary file 2 — Supplementary Information [file 42004_2024_1207_MOESM2_ESM.pdf]

Supplementary Information

Substrate Scope Expansion of 4-Phenol Oxidases by Rational Enzyme Selection and Sequence-Function Relations

Daniel Eggerichs, Nils Weindorf, Heiner G. Weddeling, Inja M. Van der Linden and Dirk Tischler*

Content

Supplementary Note 1: Amino acid cluster analysis 3
Description of the Amino Acid Cluster Analysis (A^2CA) tool 3
User guide 4
Active side comparison of 4-phenol oxidoreductases 8
Supplementary Methods 1: Heterologous production of wildtype 4-phenol oxidases 12
Protein yields 13
SDS-PAGE analysis and absorption spectra 14
Supplementary Methods 2: Sequence analysis of 4-phenol oxidases 18
Codon optimized sequences 18
Multiple Sequence alignment 21
Supplementary Methods 3: Initial enzyme characterization 23
Thermal stability and solvent tolerance 23
Initial rates for substrate conversion 25
Substrate overview and products detected by GC-MS 27
Supplementary Methods 4: Substrate binding in 4-phenol oxidases 31
Substrate binding in *Ao*EUGO 32
Substrate binding in *Asp*EUGO 33
Supplementary Methods 5: Buffer optimization 34
Comparison of *A*spEUGO and *Gb*EUGO 34
Supplementary Methods 6: Michaelis-Menten kinetics 37
Michaelis-Menten kinetics of *A*spEUGO and *Gb*EUGO 38
Supplementary Methods 7: Correlation of first shell residues of the catalytic center with substrate selectivity 40
Correlation of residue type in *ortho* position 41
Correlation of residue type in *para* position 42
Supplementary Note 2: Oxidase screening 44
Background 44
Overview 44
Evaluation 45

Supplementary Methods 8: Creation and characterization of enzyme variants 47
List of mutagenesis primers 48
List of created variants..49
Activity of purified enzyme variants 50
Crude extract assay for *Sc*EUGO A166X and V427X libraries for target (i) 51
Michaelis-Menten kinetics of *Gc*4EPO wildtype and V166D variant 52
Structural analysis of Gc4EPO V166D 54
Michaelis-Menten kinetic and structural analysis of *Sc*EUGO Q425E 55
Crude extract assay for *Sc*EUGO L381X and V427X libraries for target (iii)......................56
Michaelis-Menten kinetic and structural analysis of *Sc*EUGO V427I L282M 57
Structural analysis of *Sc*EUGO V427I 59
Supplementary References 60

Supplementary Note 1: Amino acid cluster analysis

Description of the Amino Acid Cluster Analysis (A^2CA) tool

The tool is written in the programming language R and R studio (Vers. 2023.03.0+386, "Cherry Blossom") was used as editor. The script was created under R version 4.2.0 (“Vigorous Calisthenics”). For higher versions, compatibility issues are reported. The tool can be downloaded from: www.doi.org/10.57760/sciencedb.09549

A^2CA visualizes selected residues from a multiple sequence alignment (MSA) in a phylogenetic context. Therefore, the input of a MSA and a corresponding phylogenetic tree is required. Further, characteristics of selected amino acids can be computed and included in the visualization. In the following, the computing logic of the program will be outlined. For a user guide, see the next section.

After successful upload of the input files, the user can select a reference sequence. Upon selection, the respective amino acid sequence is loaded from the alignment file into the residue selection menu. While extracting the residues, the temporary file Target_AA is written which contains the respective residue and the alignment position including gaps. After the user selects a residue from the list, the alignment position is used to extract residues for all sequences into the file Selection_Matrix. This file contains, the identifier of each sequence in the first column and residues at selected positions in the following columns represented in single letter code. Based on the user selection for amino acid characteristics, the single letter code is subsequently replaced by the respective value in the file Parameter_Matrix. The matrix has the same dimension as the Selection_Matrix, but the numerical values allow for calculations. The user can choose between the calculation of the sum or the average of the values for selected amino acid residues. As each row of the Parameter_Matrix contains the values for an individual sequence, the row values are calculated and saved in the RowValue_Matrix. The calculated values from this matrix are added as last row to the Selection_Matrix which results in the final table which is displayed in the software (Displayed_Table).

For visualization, the phylogenetic tree from the input file is used as first layer in a facet-plot. Successively, additional layers are added during the calculation process. The selected amino acids are represented as a heat map next to the tree tips of the phylogenetic tree. The data for the heat map is taken from the selection matrix. Thus, the heatmap represents the single letter code of each residue which is colored accordingly. As the tree and the Selection_Matrix use the same identifiers, no additional sorting of the matrix is required. For more detailed analysis, a bar chart can be added as additional layer to the facet-plot. As data for this plot, the RowValue_Matrix is used. The resulting facet-pot is displayed in the software as output and can be saved in the .png, .pdf or .wmf format.

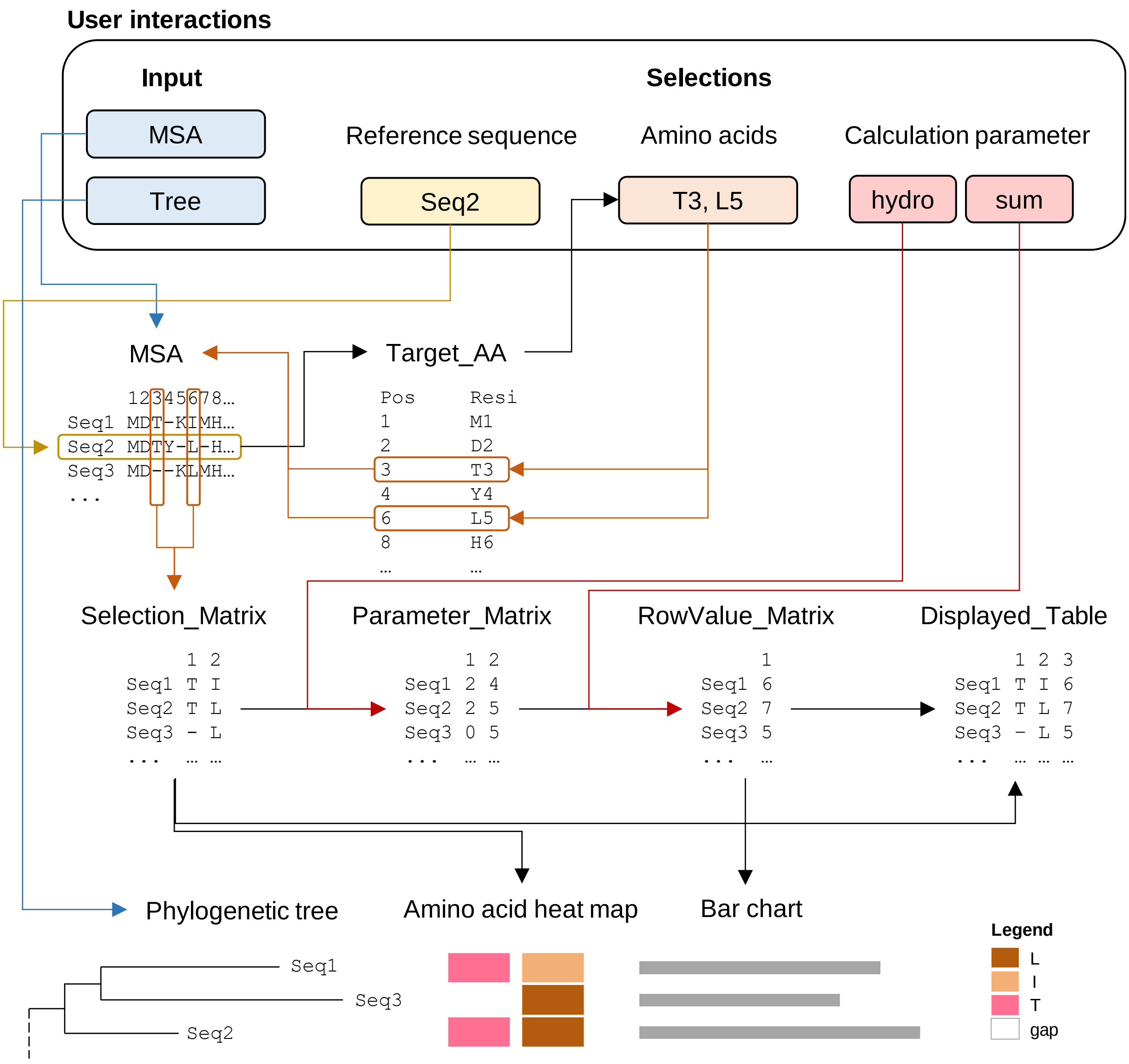

User guide

After double clicking the “run.vbs” file, the software opens in the default web browser. The version number is displayed, and the software can be entered by clicking “continue”.

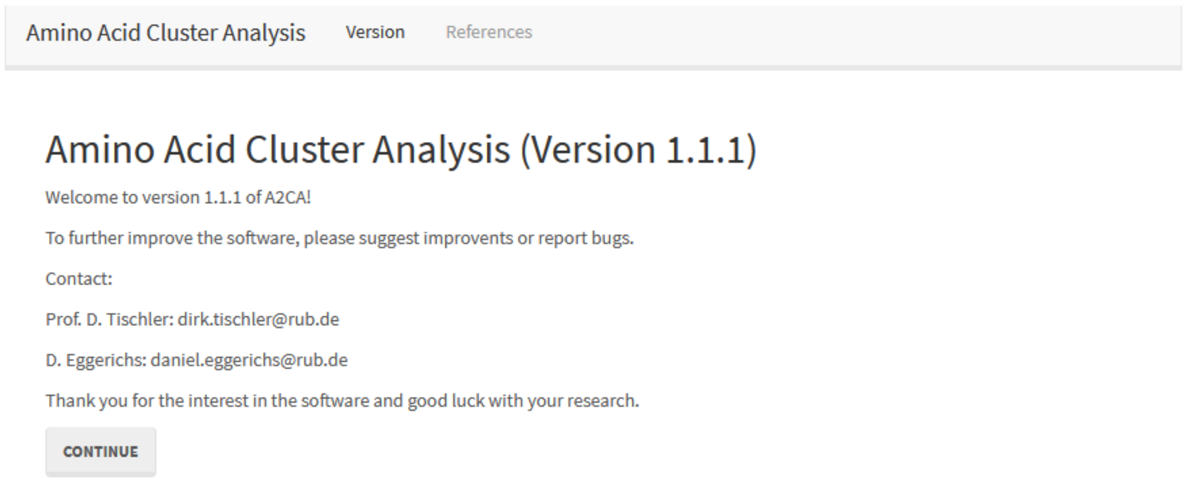

A multiple sequence alignment (MSA) and a phylogenetic tree are required as input. Both can be uploaded in the respective sections (red box). The MSA is displayed in the “Alignment” tab (blue arrow), while the phylogenetic tree is displayed in the “Tree” tab (green arrow).

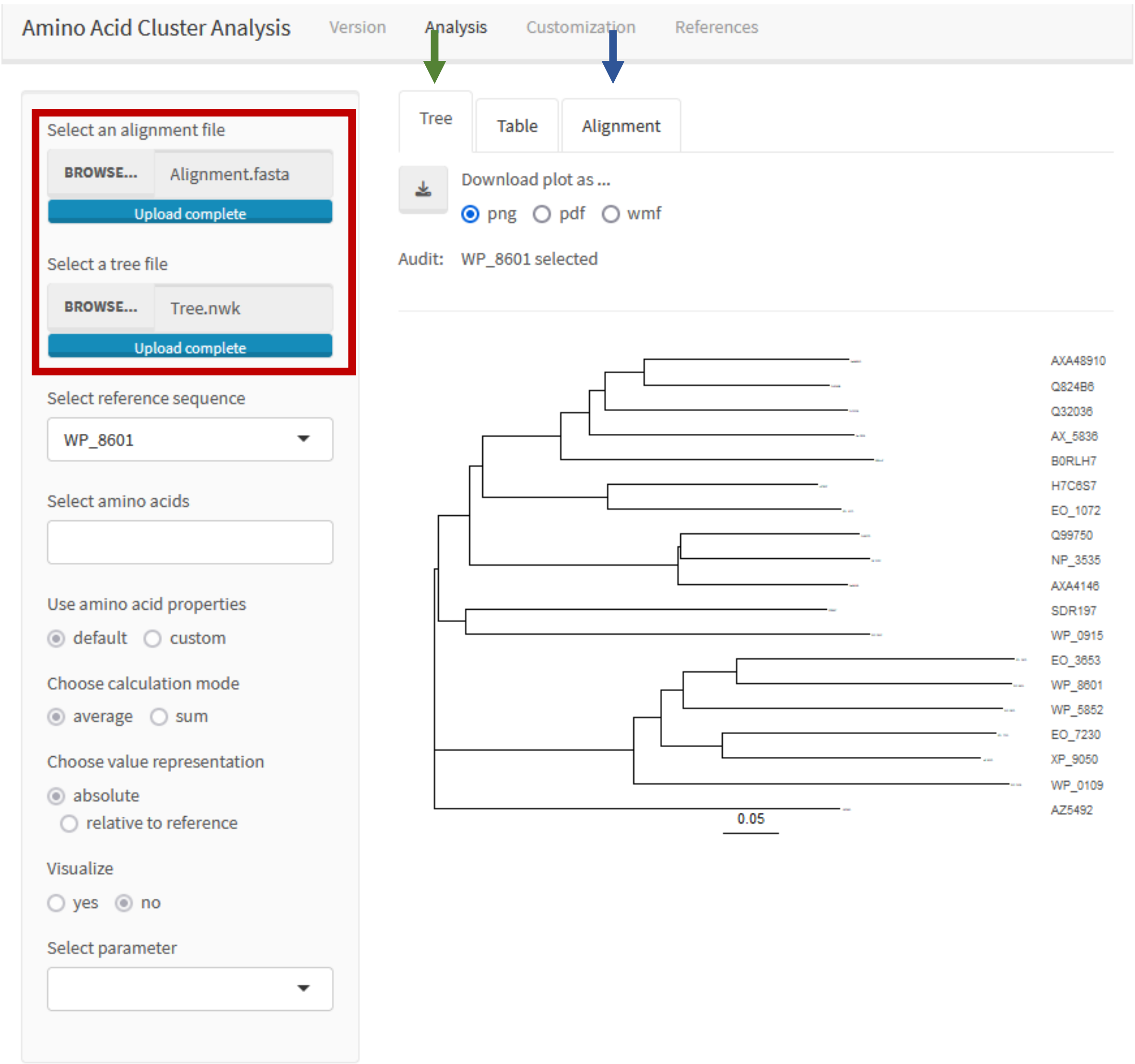

Now, a reference sequence can be selected (orange box), which enables the further selection of amino acid residues from this sequence (yellow box). Upon residue selection, the figure will be updated to show the color-coded residue for every position in the phylogenetic tree.

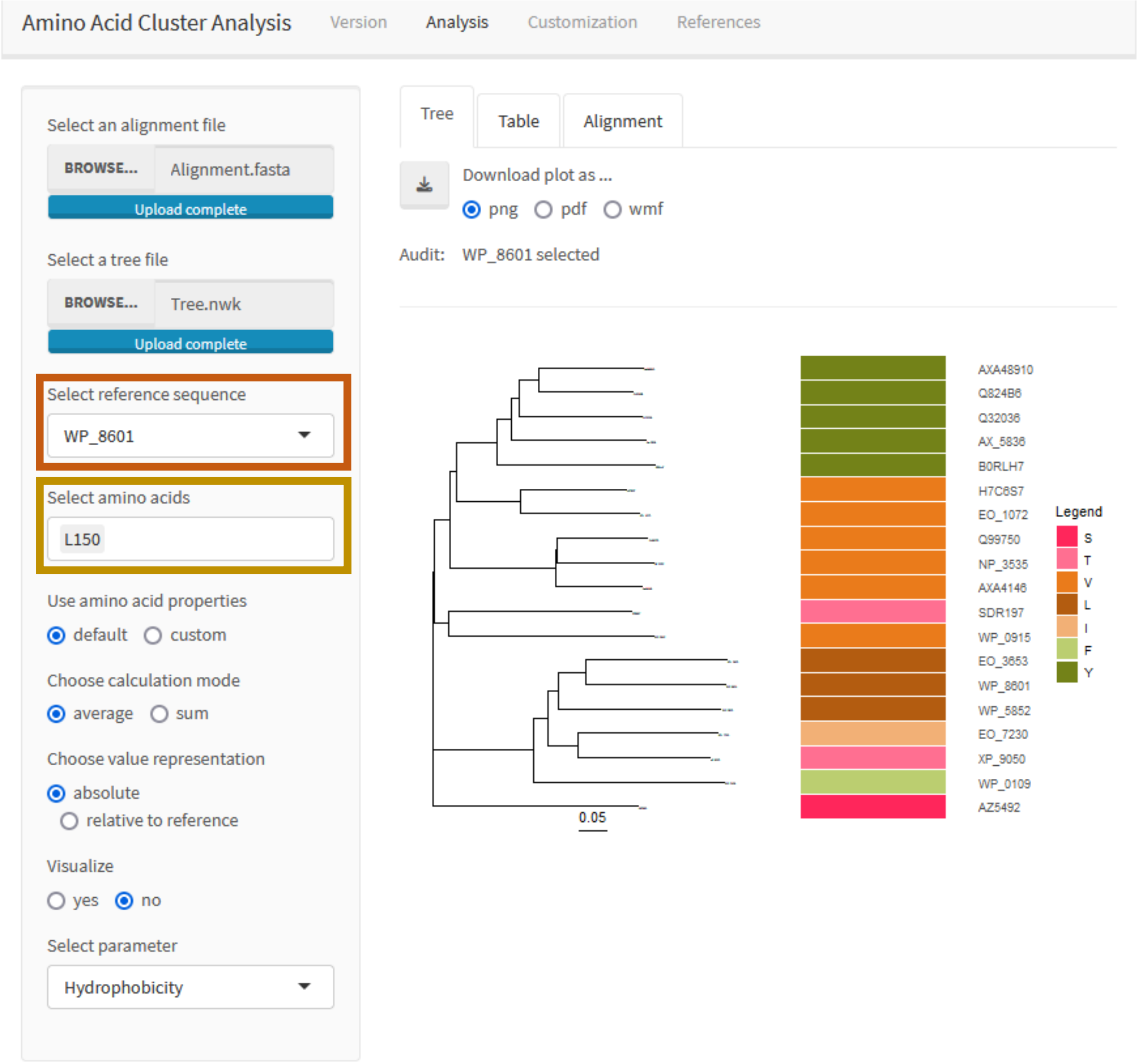

The respective parameters of the amino acid selection can be visualized as a bar chart (red arrow). The calculation parameters can be selected from a default list (green box). Alternatively, own parameters can be uploaded in the “Customization” section. The calculation mode can be chanced according to the requirements (blue box). The plot can be saved as a graphic by clicking the download button. The raw data can be accessed in the “Table” tab and downloaded accordingly.

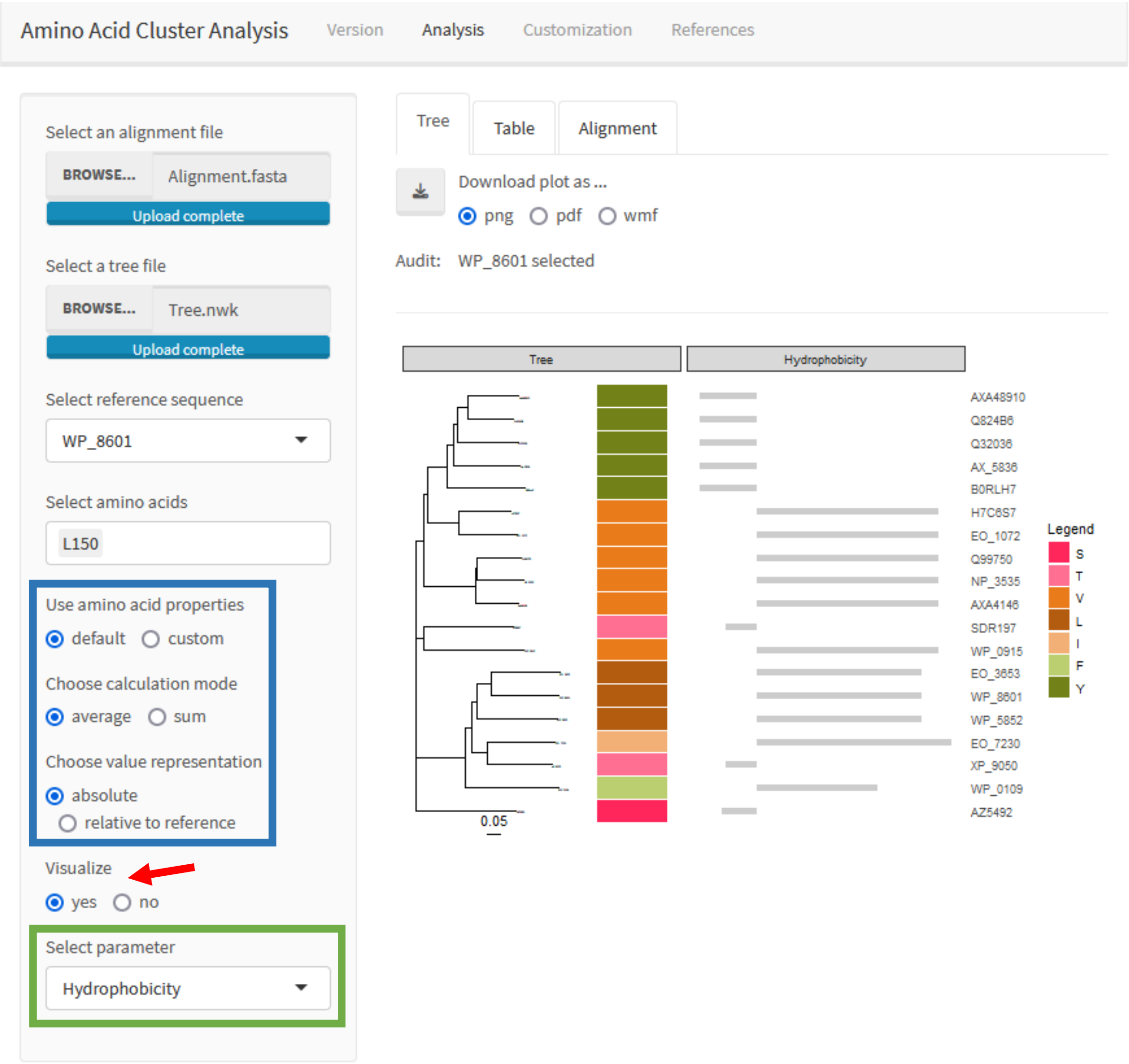

Active side comparison of 4-phenol oxidoreductases

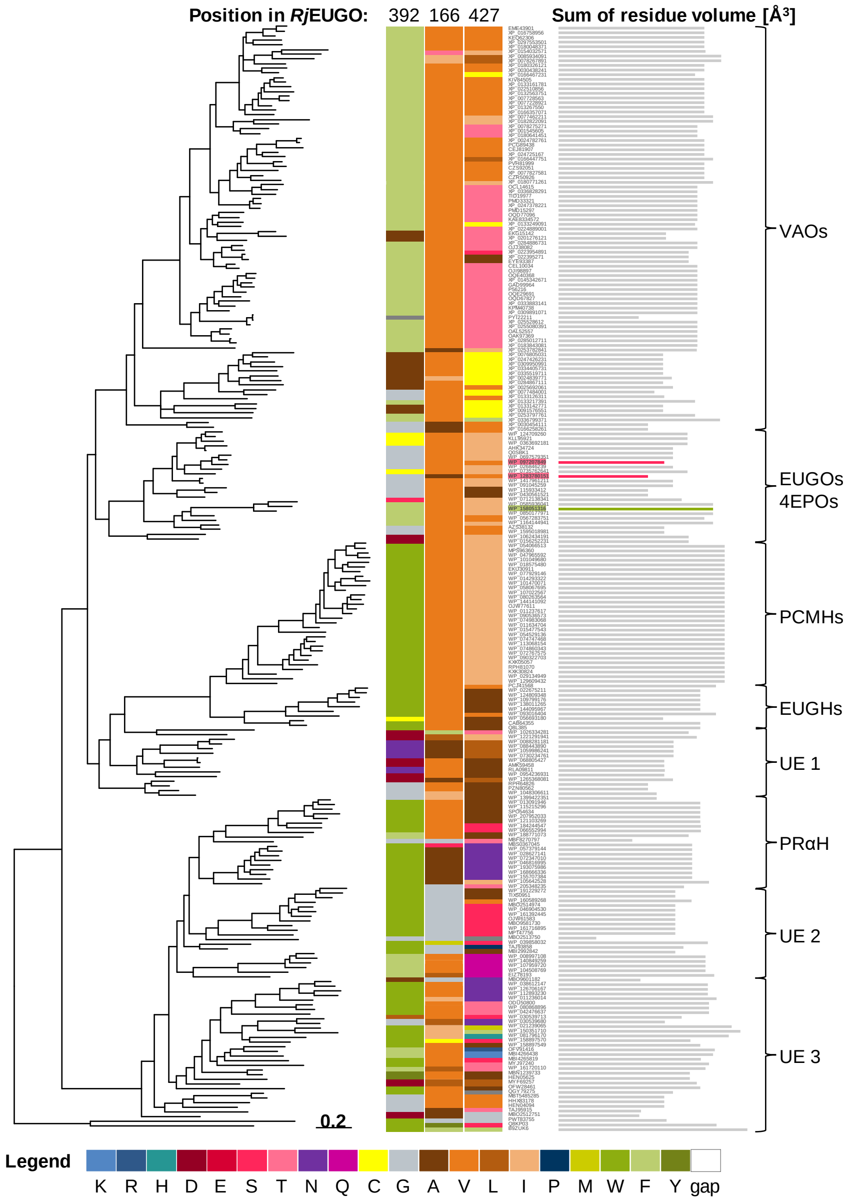

Figure S1. Comparison of the sum of the residue size for each sequence in the family of 4-phenol oxidoreductases. Based on the sequence of *Rj*EUGO, positions 392 (H-cluster), 166 and 427 (T-cluster) were selected for the comparison as both clusters interact with the *ortho* substituent of the substrate molecule. Two enzymes from *Streptomyces cavernae* (WP_1283780151) and *Geodermatophilus sabuli* (WP_097207849) of the subfamily of bacterial 4-phenol oxidases (light red) were selected for further studies as they contain a comparably wide catalytic pocket. Also, the enzyme from *Gulosibacter chungangensis* (WP_158051316) was chosen due to a comparably narrow catalytic pocket (light green). The data for the figure was calculated by and exported from A^{2}CA. Primary data is provided in Supplementary Data 1.

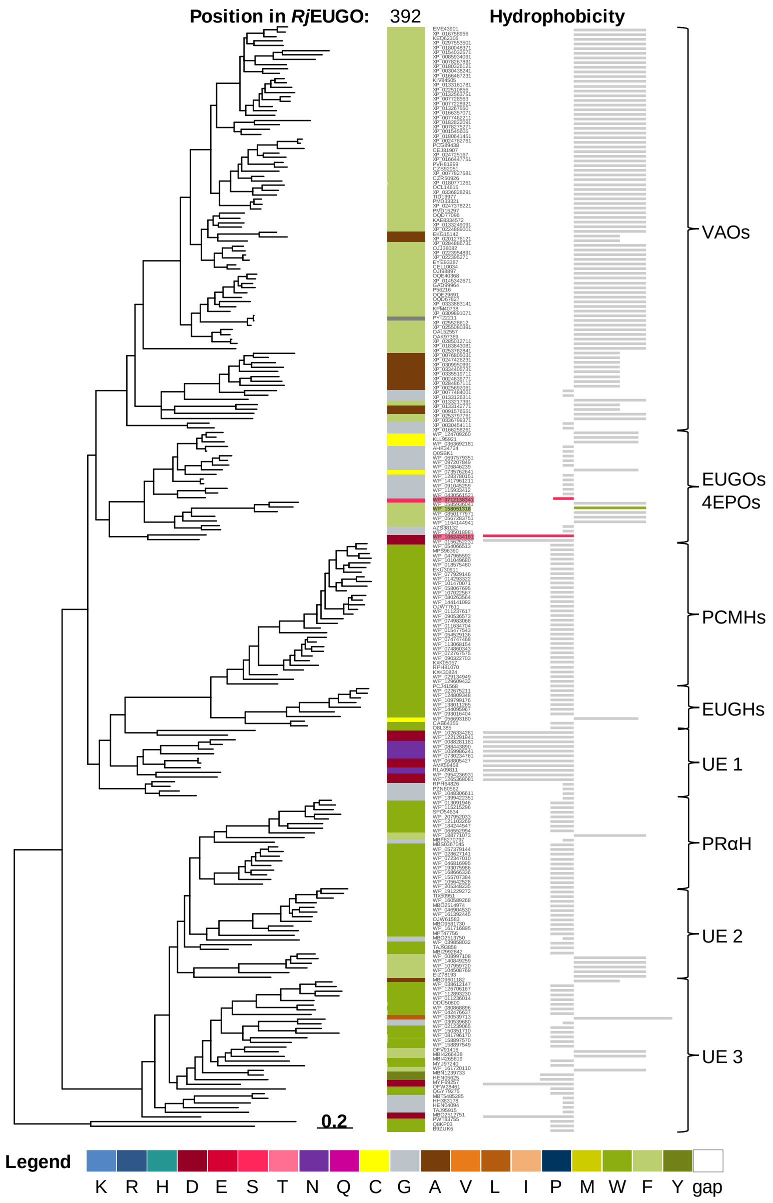

Figure S2. Comparison of the hydrophobicity of residues in position 392 (H-cluster) (*Rj*EUGO numbering) for each sequence in the family of 4-phenol oxidoreductases. Two enzymes from *Arthrobacter* sp. (WP_0712138341) and *Allonocardiopsis opalescens* (WP_1062434191) were identified with a more polar residue in this position and were selected for further characterization (light red). Also, the enzyme from *Gulosibacter chungangensis* (WP_158051316) was chosen due to a comparably hydrophobic residue in this position (light green). The data for the figure was calculated by and exported from A^{2}CA. Primary data is provided in Supplementary Data 1.

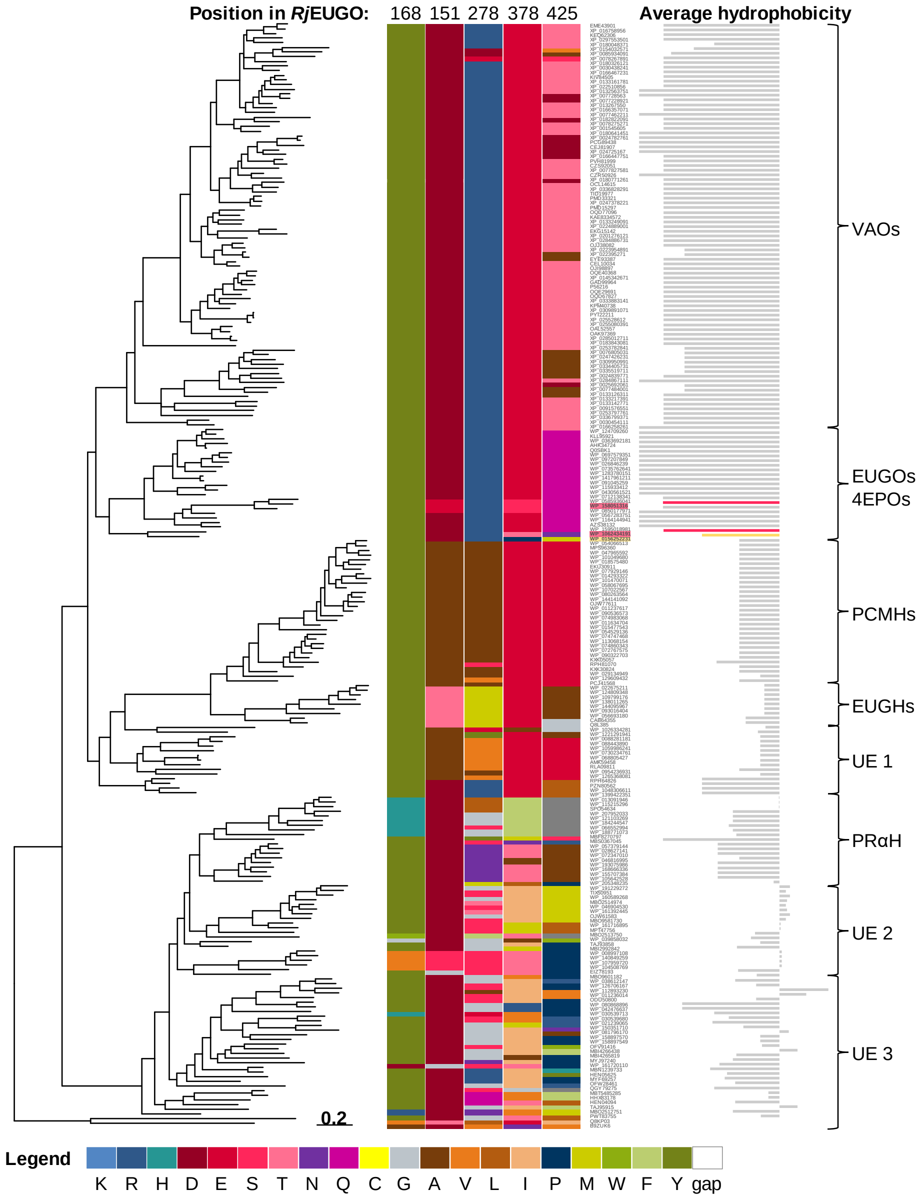

Figure S3. Comparison of the average hydrophobicity of residues in the W-cluster for each sequence in the family of 4-phenol oxidoreductases. Two enzymes from *Gulosibacter chungangensis* (WP_158051316) and *Allonocardiopsis opalescens* (WP_1062434191) were identified with a less polar cluster and were selected for further characterization (light red). The enzyme from *Actinoplanes* sp. N902-109 (WP_015625223) contained the comparably most hydrophobic W-cluster but was not included in this study (light yellow). The data for the figure was calculated by and exported from A^{2}CA. Primary data is provided in Supplementary Data 1.

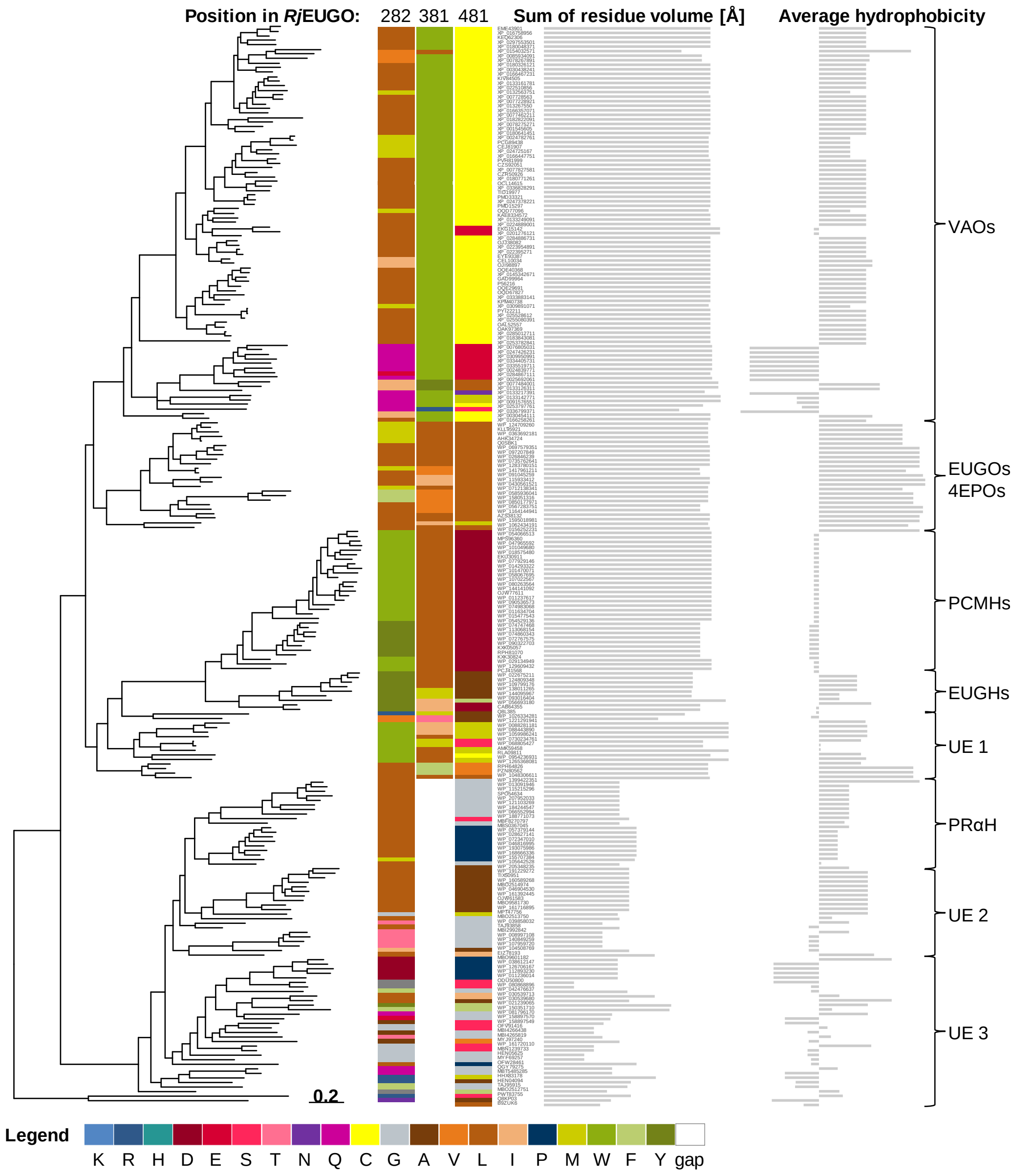

Figure S4. Comparison of the residue size and the average hydrophobicity of residues in the A-cluster for each sequence in the family of 4-phenol oxidoreductases. For many subclades, the residue size is quite similar. The third major clade sticks out due to a gap in the alignment. More subclade specific differences are observed with regard to hydrophobicity. The data for the figure was calculated by and exported from A^{2}CA. Primary data is provided in Supplementary Data 1.

Supplementary Methods 1: Heterologous production of wildtype 4-phenol oxidases

For heterologous protein production, codon-optimized sequences were synthesized and cloned in pET16bp vectors. Most proteins could be produced in *E. coli* BL21 (DE3) with yields ranging between 20 and 160 mg L^{-1} (Table S1). The expression of *Gs*EUGO was only possible by co-expression of the chaperone vector pKE7. The pBADNk vector encoding for *Rj*EUGO was kindly provided by Prof. M. Fraaije (Groningen, The Netherlands).

Gibson-assembly

Codon-optimized genes were synthesized by Twist Bioscience and resulting gene fragments were cloned into pET16bp vectors by means of Gibson-assembly.[1] All PCRs were performed using the PrimeSTAR Max DNA-polymerase (Takara) with 25 µL of total reaction volume, 100 ng of template DNA, and 10 ng of the respective primers. Treatment with DpnI (10 U) for 1.5 hours at 37°C removed template DNA. Successful amplification was verified by agarose gel electrophoresis using a 1 % agarose gel containing in-gel dye, SERVA HiSens Stain G. For Gibson-assembly, 3.75 µL of Gibson master mix (NEB) were mixed with 0.5 µL of plasmid backbone and 1 µL of the gene of interest, as well as 2.25 µL of water. After one hour incubation at 50°C, a heat shock transformation in *E. coli* DH5α was performed. For validation, plasmids were obtained from transformants and controlled by sequencing before transformation in the respective expression strain (Table S1) was conducted.

Heat shock transformation

100 ng of plasmid were mixed into the 50 µL aliquots of chemically competent *E. coli* cells which were incubated on ice for 20 minutes. The heat shock was performed at 42°C for 45 seconds followed by another 2 minutes incubation on ice. Afterwards, 950 µL of LB-medium (10 g L^{-1} tryptone, 5 g L^{-1} yeast extract, 10 g L^{-1} sodium chloride) were added and incubation for 90 min at 37°C was performed for recovery. The cells were harvested by centrifugation (5 min at 5,500 x g) and ~900 µL of the supernatant were discarded. The cell pellet was resuspended in the remaining 100 µL and subsequently streaked out onto a LB-agar plate, containing the respective antibiotics (100 mg L^{-1} ampicillin for pET16bp and pBADNk, and additional 50 mg L^{-1} chloramphenicol for pKJE7). The plates were incubated at 37°C overnight and transformants were observed the next day.

Main culture

A 50 to 100 mL preculture in LB-medium (10 g L^{-1} tryptone, 5 g L^{-1} yeast extract, 10 g L^{-1} sodium chloride) containing the respective antibiotics (100 mg L^{-1} ampicillin for pET16bp and pBADNk, and additional 50 mg L^{-1} chloramphenicol for pKJE7) was inoculated with the respective expression strain and was grown overnight at 37°C. The preculture was used to inoculate TB auto-induction medium (12 g L^{-1} peptone, 24 g L^{-1} yeast extract. 100 mM potassium phosphate buffer pH 7.0, 0.5 g L^{-1} glucose, 2.0 g L^{-1} lactose, 5.0 mL L^{-1} glycerol) to a starting OD_{600} of 0.05. Either 1 L medium in a 2.8 L Fernbach flask or 4.5 L medium in a 5 L fermenter were used. After inoculation, the cultures were shaken (Fernbach flask, 130 rpm) or stirred (fermenter, 300 rpm, 2 L min^{-1} air supply) for 4 hours at 37°C, reaching an OD_{600} of about 1.0. Then, the temperature was reduced to 25°C and cultures were incubated overnight. Final OD_{600} ranged from 5 to 8 in Fernbach flasks and reached 8 to 10 in the fermenter.

Cell harvest and protein purification

The main cultures were harvested by centrifugation (20 min at 5,000 x g and 4°C) and the cell pellet was washed in 30 mL 100 mM potassium phosphate buffer pH 7.0 per liter of culture medium. The resuspension was transferred into 50 mL tubes and centrifuged again. The resulting cell pellets were either directly used for cell lysis or stored at -20°C until further use.

For lysis, cell pellets were resuspended in 30 mL of buffer A (10 mM Tris pH 7.5, 500 mM sodium chloride), before treated with 15 cycles by sonication (Bandelin sonoplus). Cell debris were removed by centrifugation (1.5 h at 20,000 x g and 4°C) and the supernatant was filtered through a 0.2 µm pore before being used for protein affinity chromatography, which was performed with an Äkta Start FPLC system (GE Healthcare) equipped with a 5 mL Ni-NTA-HisTrap column. Elution was performed stepwise with 20% and 100% buffer B (10 mM Tris-HCl pH 7.5, 500 mM NaCl, 500 mM imidazole) in buffer A. During the whole process, the absorbance at 280 nm was monitored to collect protein containing fractions. Further, SDS samples were collected (Figures S5a to S12a). Yellow fractions were pooled, and the buffer was exchanged overnight at 8°C to 50 mM potassium phosphate buffer pH 7.5 by means of dialysis. Dialyzed proteins were transferred into 1.5 mL tubes and a final glycerol concentration of 60% was added for storage at -20°C. SDS samples were collected during the purification.

Determination of protein concentration

To determine the concentration of cofactor loaded protein, the absorption of the histidinyl-bound FAD-cofactor was measured at 441 nm (ε = 14,200 L mol^{-1} cm^{-1}, Figures S5b to S12b).[2] The absorption was transformed into cofactor concentration using the Lambert-Beers law.

Protein yields

Table S1. Overview about enzymes used in this work.

Donor organism	Accession number	Designation (this study)	Vector backbone	Host organism	Yield [mg L^{-1}]	Source
Streptomyces cavernae	WP_128378015	*Sc*EUGO	pET16bp	*E. coli* BL21 (DE3)	28	This study
Geodermatophilus sabuli DSM 46844	WP_097207849	*Gs*EUGO	pET16bp	*E. coli* NiCo21 (DE3) + pKE7	2.4	This study
Rhodococcus jostii RHA1	Q0SBK1	*Rj*EUGO	pBADNk	*E. coli* Top10	94	Jin et al.[2]
Nocardioides sp. YR527	WP_091045259	*N*spEUGO	pET16bp	*E. coli* BL21 (DE3)	172	Eggerichs et al.[3]
Arthrobacter sp. UCD-GKA	WP_071213834	*A*spEUGO	pET16bp	*E. coli* BL21 (DE3)	106	This study
Geodermatophilaceae bacterium URHA0031	WP_026846239	*Gb*EUGO	pET16bp	*E. coli* BL21 (DE3)	52	This study
Allonocardiopsis opalescens DSM 45601	WP_106243419	*Ao*EUGO	pET16bp	*E. coli* BL21 (DE3)	25	This study
Gulosibacter chungangensis KCTC 13959	WP_158051316	*Gc*4EPO	pET16bp	*E. coli* BL21 (DE3)	24	Alvigini et al.[4]

SDS-PAGE analysis and absorption spectra

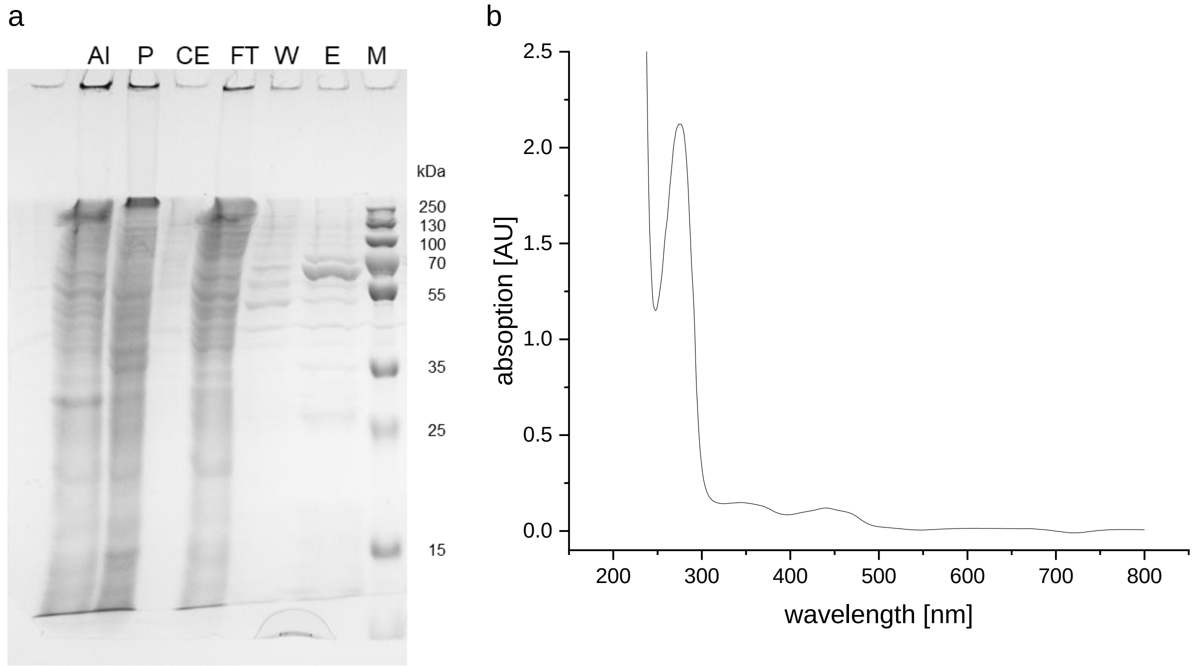

Figure S5. a SDS PAGE from the expression of the 4-phenol oxidase from *Streptomyces cavernae* (*Sc*EUGO). AI: after induction, P: pellet, CE: crude extract, FT: flow through, W: wash, E: elution, M: Marker (PAGE-Ruler Plus, Thermo Scientific) **b** UV/vis spectrum of the elution fraction after dialysis.

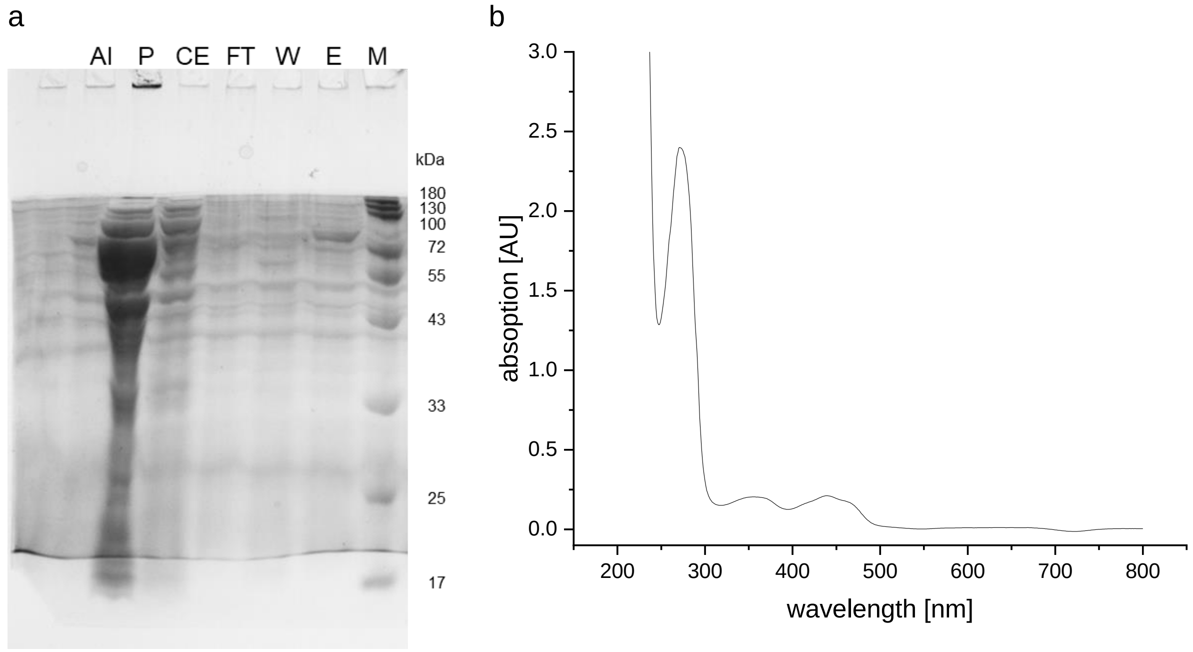

Figure S6. a SDS PAGE from the expression of the 4-phenol oxidase from *Geodermatophilus sabuli* DSM 46844 (*Gs*EUGO). AI: after induction, P: pellet, CE: crude extract, FT: flow through, W: wash, E: elution, M: Marker (Prestained Protein Ladder (10-180 kDa), Cohesion Biosciences) **b** UV/vis spectrum of the elution fraction after dialysis.

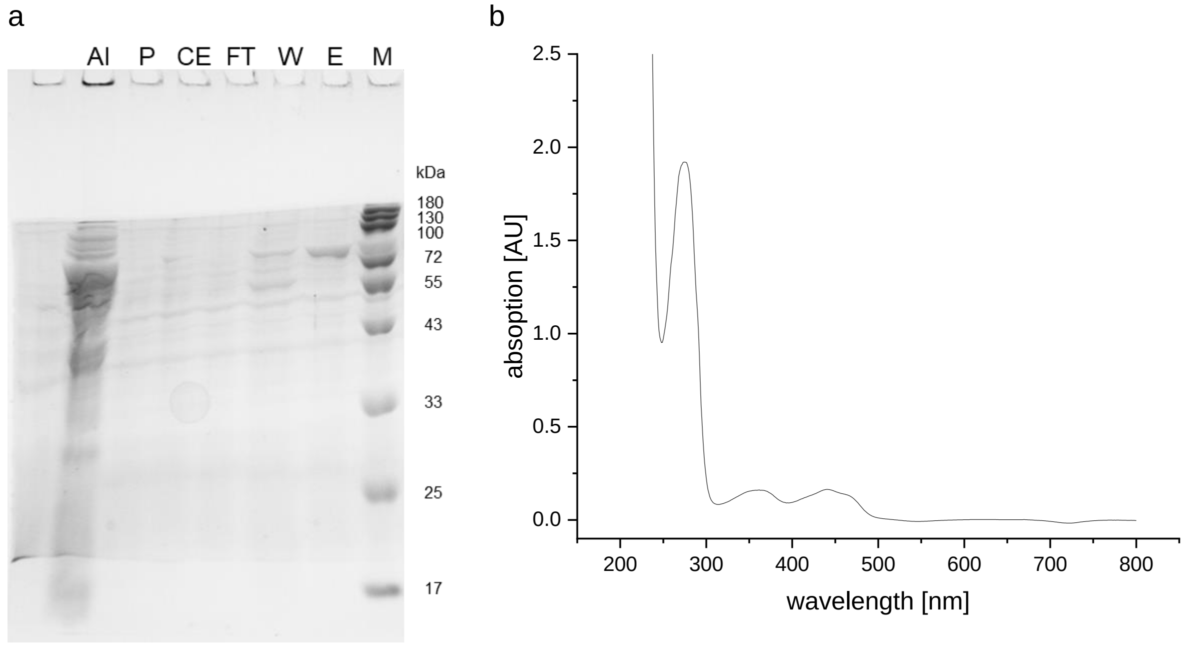

Figure S7. a SDS PAGE from the expression of the 4-phenol oxidase from *Rhodococcus jostii* RHA1 (*Rj*EUGO). AI: after induction, P: pellet, CE: crude extract, FT: flow through, W: wash, E: elution, M: Marker (Prestained Protein Ladder (10-180 kDa), Cohesion Biosciences) **b** UV/vis spectrum of the elution fraction after dialysis.

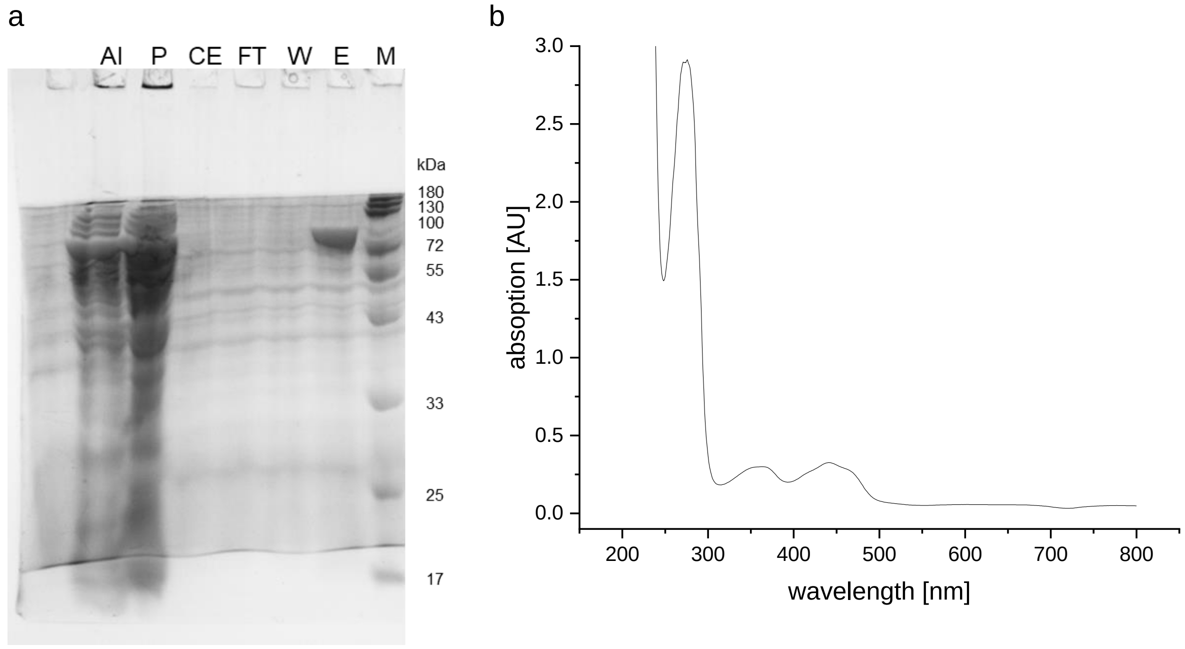

Figure S8. a SDS PAGE from the expression of the 4-phenol oxidase from *Nocardioides* sp. YR527 (*N*spEUGO). AI: after induction, P: pellet, CE: crude extract, FT: flow through, W: wash, E: elution, M: Marker (Prestained Protein Ladder (10-180 kDa), Cohesion Biosciences) **b** UV/vis spectrum of the elution fraction after dialysis.

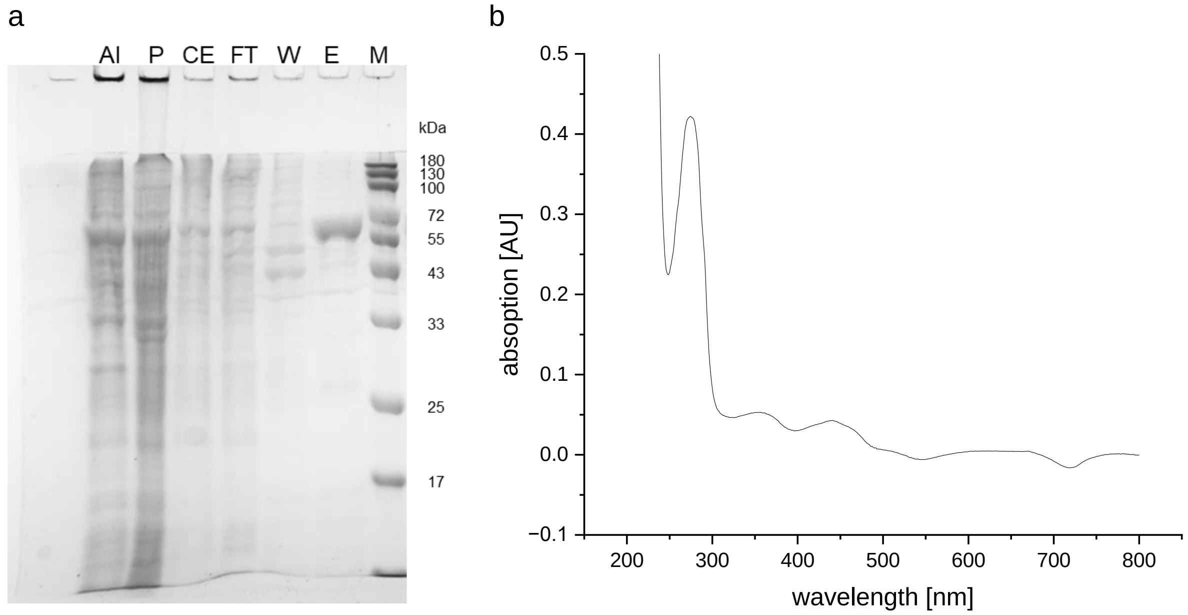

Figure S9. a SDS PAGE from the expression of the 4-phenol oxidase from *Arthrobacter* sp. UCD-GKA (*A*spEUGO). AI: after induction, P: pellet, CE: crude extract, FT: flow through, W: wash, E: elution, M: Marker (Prestained Protein Ladder (10-180 kDa), Cohesion Biosciences) **b** UV/vis spectrum of the elution fraction after dialysis.

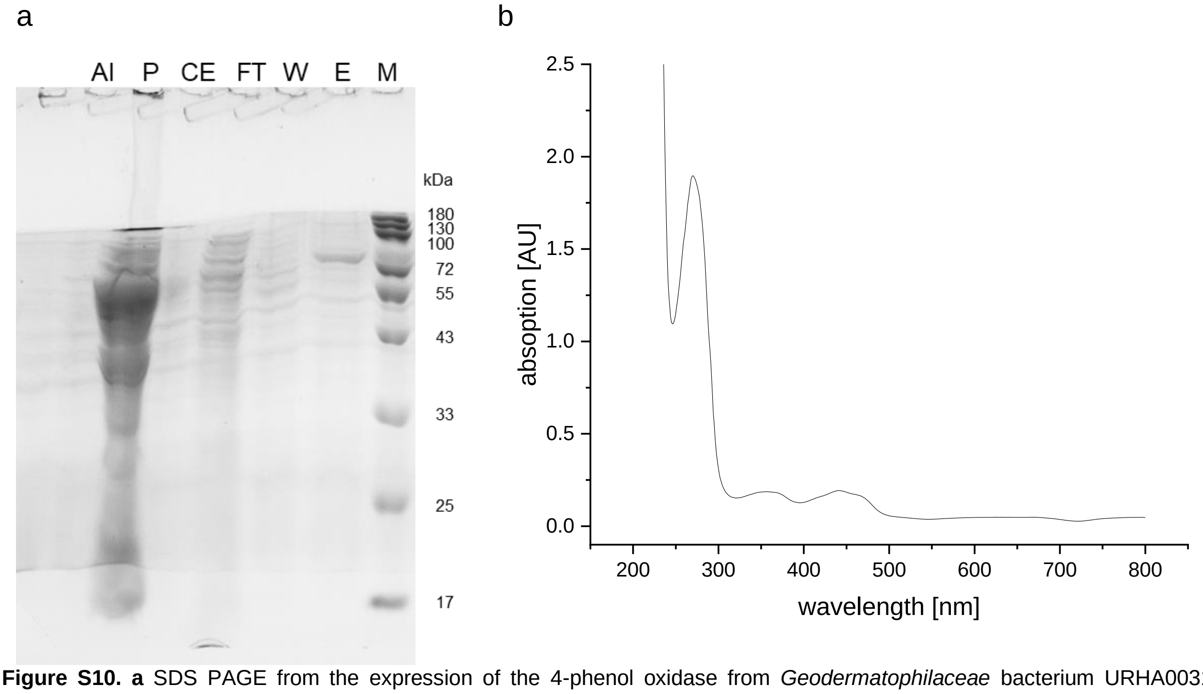

Figure S10. a SDS PAGE from the expression of the 4-phenol oxidase from *Geodermatophilaceae* bacterium URHA0031 (*Gb*EUGO). AI: after induction, P: pellet, CE: crude extract, FT: flow through, W: wash, E: elution, M: Marker (Prestained Protein Ladder (10-180 kDa), Cohesion Biosciences) **b** UV/vis spectrum of the elution fraction after dialysis.

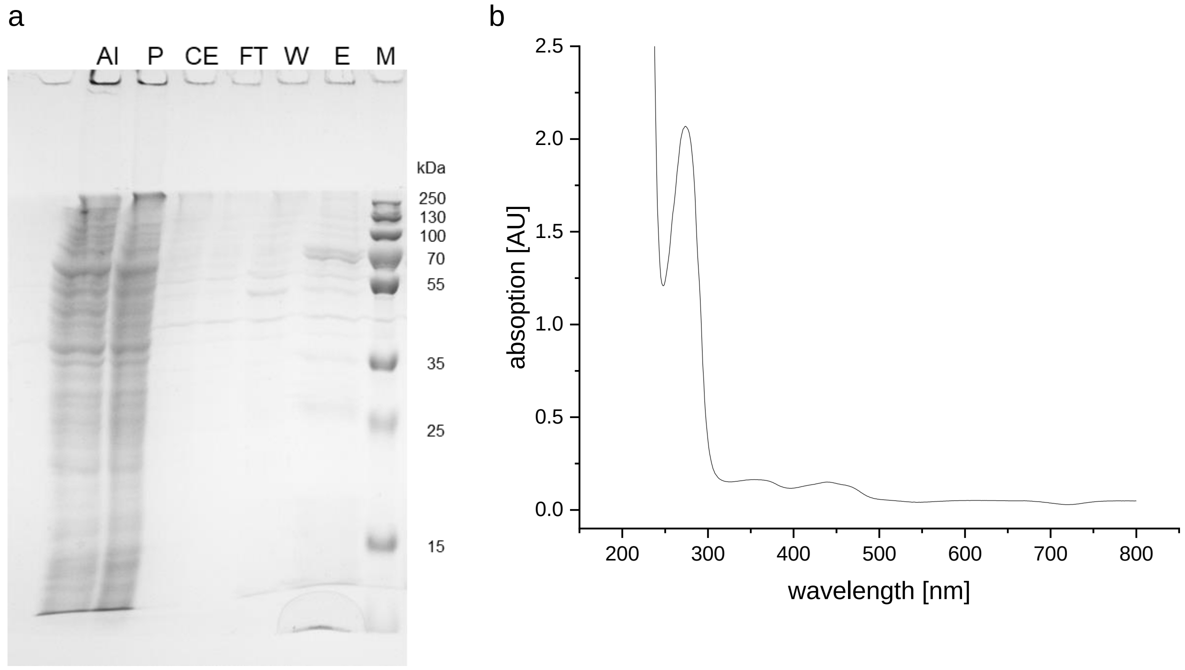

Figure S11. a SDS PAGE from the expression of the 4-phenol oxidase from *Allonocardiopsis opalescens* DSM 45601 (*Ao*EUGO). AI: after induction, P: pellet, CE: crude extract, FT: flow through, W: wash, E: elution, M: Marker (PAGE-Ruler Plus, Thermo Scientific) **b** UV/vis spectrum of the elution fraction after dialysis.

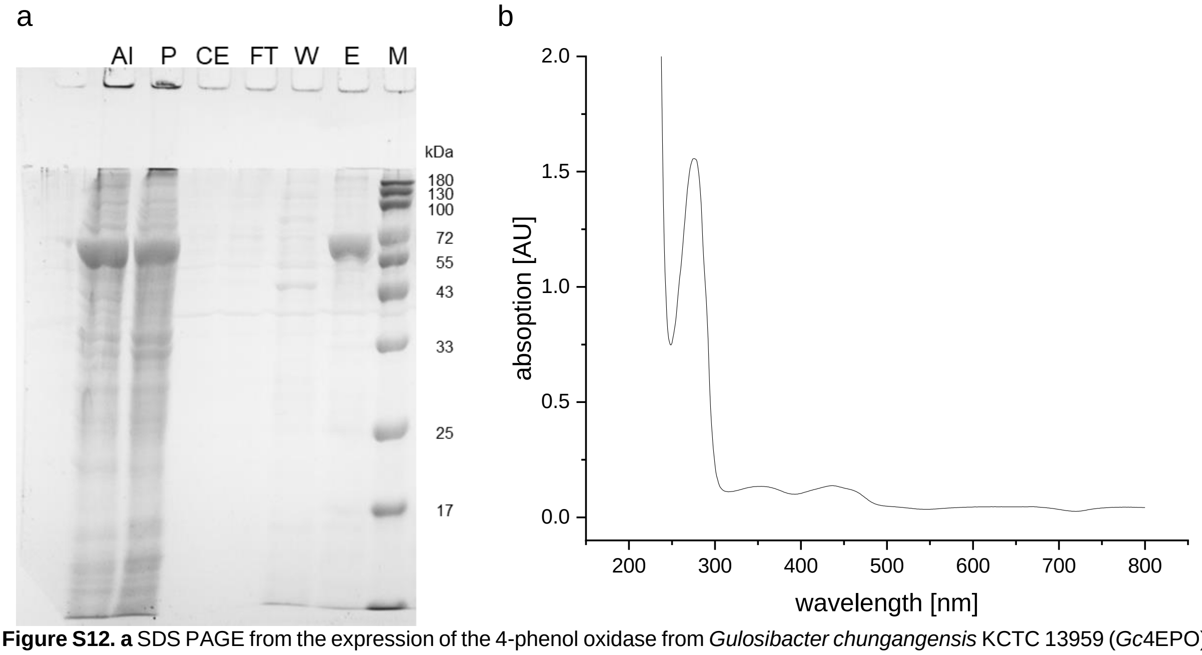

Figure S12. a SDS PAGE from the expression of the 4-phenol oxidase from *Gulosibacter chungangensis* KCTC 13959 (*Gc*4EPO). AI: after induction, P: pellet, CE: crude extract, FT: flow through, W: wash, E: elution, M: Marker (Prestained Protein Ladder (10-180 kDa), Cohesion Biosciences) **b** UV/vis spectrum of the elution fraction after dialysis.

Supplementary Methods 2: Sequence analysis of 4-phenol oxidases

Codon optimized sequences

> WP_128378015 [*Streptomyces cavernae*]

```
ATG ACG CGC ACA CTG CCG CCT GGC GTG AGT GAT GAG GAC TTC ACC AGC GCG CTG ACC GCA TTT CGC GAT
GTT GTG GGT GAC GAG TTT GTT CGC ACG GAT GAG GCT GAA CTG GCC CGC TTT CAC GAT CCG TAC CCG GTT
GGA GAT GCT GAT GCT CAT TTA GCC TCT GCG GTG ATT AGC CCT CGC GAC ACG GAA CAA GTA CAG GAA GTC
GTG CGC ATT GCA AAC CGC TAT GGC ATT CCG CTT TCG GTG ATT TCA ACT GGC CGG AAT AAT GGC TAT GGC
GGT AGT GCG CCG CGT TTA AGC GGC GCG GTT GTT GTG AAT ACG GGC GAA CGC ATG AAC CGC ATT CTG GAA
GTG GAT GAG AAA CTG GGA TAC GCG CTG TTG GAA CCT GGC GTG ACA TAC TTC GAT CTG CAC GAA TAC CTT
GAA GCC CAT GCA CCG TCG TTA ATG ATT GAC TGC CCG GAT CTG GGT TGG GGT TCG GTG GTT GGG AAC GCG
TTA GAT CGT GGG GCA GGC TAT ACC CCG TAT GGG GAT CAC TTC ATG TGG CAA ACT GGT ATG GAA GTA GTC
CTT CCA CAG GGT GAT GTT ATG CGT ACT GGC ATG GGC GCC TTA CCG GGT AGC ACG ACA TGG CAG CTC ATT
CCG TAT GGT TTT GGA CCA TAC CCA GAC GGC ATG TTC ACC CAG TCC AAC CTG GGT ATT GTC ACG AAA ATG
GGC ATT GCA CTC ATG CAG AAA CCG CCA GCG TCC ATG ACC TAT CAG ATC ACG TTT GAG AAC GAA AGC GAT
CTG GAG CAG ATC GTC GAC ATC ATG CTG CCA CTG CGT ATC AAT ATG GCT CCG CTG CAG AAT GTA CCG GTT
CTG CGC AAC ATC ATC CTC GAT GCC GCC GTA GTG TCT CAA CGG GCC GAT TGG TAC GAT GGG GAT GGG CCT
CTG CCG CCC GAA GCG ATC GAA CGC ATG AAG AAA GAG CTG GGC TTG GGT TAC TGG AAT TTC TAC GGC ACC
CTG TAT GGC CCA CCG CAA CTC ATC GAA ATG AAC TAC GGC ATT ATT AAG GAC GCC TTT GGC CAG ATT CCT
GGT TCA CGC TTT CAG ACC CAT GAG GAA CGT CAC GAT CGT GGA GCA CAT GTC TTG CAA GAT CGC CAC AAA
ATC AAC AAT GGT ATC CCC TCC CTG TCT GAG ATG AAA CTT ATG GAC TGG ATT CCC GGT GCA GGA CAT GTC
GGT TTT AGC CCG ATC AGT CCG CCG GTA GGT CGT GAC GCT ATG AAA CAG TTC CGC ATG GTG CGT TCA CGT
GCG GAC GAA TAT GCG AAG GAC TAT GCA GCG CAG TTT GTG GTC GGG TTA CGG GAA ATG CAC CAT ATT GCG
CTG CTT CTG TTT GAT ACC CAA GAC GCG ACA GCA CGT AAT GAA ACC TTG GCC TTG ACT CGT CTG CTG ATT
GAT GAA GCT GCT GCC GAA GGG TAT GGC GAA TAT CGT ACC CAT AAT GCC CTG ATG GAT CAA GTT ATG GGC
ACC TAT AAC TGG GGC GAT GGC GCG CTG CTG AAA TTC CAT GAA GCG ATC AAA GAC GCC CTC GAC CCC AAC
GGT ATT ATT GCG CCT GGT AAA TCG GGT GTG TGG CCA GCA CGC TAT CGC GGG AAA GGA TTG GCG TAG
```

>WP_097207849 [*Geodermatophilus sabuli* DSM 46844]

```
ATG GCA CGC TTG CTT CCA CCA GGT CTG TCT GAG TCG GAT TTT GAT GCC GCC ATT GCG CGT TTC CGC GAT
GTA GTA GGC GAC AAA TAC GTC GTA ACA GAG GAT GGG GAT TTA GCG CGT TAT CGT GAC CCG TAT CCG GTT
GGG TCT GAG CCG GCC ACT GGT GCT TCA GCT GCG ATT AGT CCT GAA AGC ACT GAA CAG GTT CAG GAA ATC
GTT CGT ATT GCG AAC GAA TAT GGT GTC CCG TTG TCG CCG ATT AGT ACC GGA CGC AAC AAC GGC TAT GGA
GGC GGG CAA CCT CGC CTT TCA GGC GCA GTC GTG GTG AAT ACC GGA GAA CGG ATG AAT CGT ATC ATC GAG
GTC AAC GAG AAG TAC GGT TAT GCC CTG CTG GAA CCA GGC GTG TCC TAT TTC GAT CTG TAC GAG TAC CTC
GAA GCC AAT GCT CCG TCC TTG ATG TTA GAC TGC CCA GAT CTG GGT TGG GGT TCA GTG GTC GGG AAC ACC
CTT GAT CGC GGA GTG GGT TAT ACG CCT TAT GGT GAC CAT CTG ATG TGG CAG ACT GGC CTG GAA GTA GTG
CTG CCT ACA GGG GAA GTG ATG CGC ACA GGC ATG GGT GCG GTA CCA GGC TCT ACT ACA TGG CAG TTG TTC
CAG TAC GGT TTT GGA CCG TTT CCG GAT GGC CTC TTC ACC CAG AGT AAT CTG GGA ATT GTT ACG AAA ATG
GGC ATT CAA CTC ATG CAG CGT CCG CCG AGC AGC ACG ACC TTC CTC ATC ACG TTC GAT CGC GAA GAG GAC
CTG GCG CAA GTC GTT GAC ATC ATG TTT CCC TTG CGC GTG AAT ATG GCC CCG CTG CAG AAT GTC CCC GTA
CTG CGT AAC ATT GTG CTG GAT GCG GGT GTT GTG TCC AAA CGG ACC GAA TGG CAT GAT GGG GAT GGT CCA
CTT CCC GCA GAA GCA ATT GAG CGC ATG AAA TCG GAA CTG GGC TTA GGC TAC TGG AAC CTG TAT GGC ACG
GTG TAT GGC CCT CCG CCT GTC GTC GAA CAA TAT CTG GGC ATG ATC CGC GAT GCC TTT CTG CAA GTT CCG
GGC TCG CGC TTT AGC ACC CAT CAT GAT CGC GAT GAA GCG ACC GAT CGT GGC GCT CAC GTG CTT CAT GAC
CGT CAT CGC ATC AAC AAC GGT ATT CCG AGT CTG GAC GAA ATG AAG CTG CTG GAA TTT GTT CCG AAT GGT
GGC CAC ATT GGT TTT AGC CCC ATC AGC GCC CCA GAT GGG GCG GAC GCC TTA CGC CAG GCT CAA ATG GTG
CGT CAG CGT GCG GAT GAA TAC CGC CAG GAT TAC GCG GCA CAG TTT GTG GTT GGC CTG CGG GAA ATG CAC
CAC ATT GCC TTG CTG TTA TTC GAC ACC ACC AAA CCG GAA CAG CGT CAA CGT GCC CTG GAC CTG GCA CGC
GTT CTC ATT GAC GAA GCA GCA GCG GAG GGG TAT GGC GAG TAC CGC ACG CAT AAT GCG CTG ATG GAT CAG
GTG ATG GGT ACG TAC GAT TGG GGT GAC GGT GCT TTA CGT CGC TTT CAC GAA ACC ATC AAG GAT GCG CTG
GAT CCG AAC TCC ATC ATG GCA CCG GGC AAA AGC GGC ATT TGG GGC CGG AAA TAT CGC GAC AAA GGT TTA
GCG TAG
```

>WP_071213834 [*Arthrobacter* sp. UCD-GKA]

```
ATG AGC CGT ATT ATT CCG CTG GAT GTA TCC GAA GCC GAC TTT GAT GCG GCG CTG GAA GAA TTT CGT GGA
GCT CTT GGG GCG CAA TGG GTT CTG AGT TCT CCA GTT GAA CTC GAA GCG TTT GCG GAC CCA TAT CCG ACC
ACG AAT GGC CTG GAG TTC CTG CCA GGT GCT GTA ATC TCG CCA TCG ACT CCA GAA GAA GTA CAG GTG ATT
GTG GGG ATT GCC AAC AAA TAC AAA GTT CCG CTG TCT CCG GTT TCC ACA GGG AAG AAT TTG GGC TAT GGC
GGT GCG GCA CCA CGC CTG TCG GGA ACA GTC GTC GTT AAC ACC GGT GAA CGG ATG AAC AAG ATC ATC GAA
GTA AAC GAG AAA TAT GCG TTT GCG CTC GTG GAA CCT GGT GTC ACG TAC TTT GAT CTG TAC AAC CAT ATT
CAG GAG AAA GGC TAC AAT TTG TGG ATT GAT GTC CCG GAC TTA GGC TGG GGC AGC ATT GTC GGC AAC ACC
CTG GAT CGT GGG GTG GGC TAT ACC CCG TAT GGT GAC CAT TGG TCA TGG CAG ACA GGG TTG GAG GTC GTT
TTG CCT GAT GGT GAT CTG CTG CGT ACG GGT ATG GGC GCT ATG TCT GGT AGC GAC GCA TGG CAG CTG TTT
CCG TAT GGC TTC GGT CCG TAC CCG GAC GGC TTG TTT TCC CAA AGC AAC TAT GGC ATT GTG ACC AAA CTG
GGC ATT GCA CTG ATG CCT GCT CCT CCG GCG AGC GAG ACT TTC CTC ATT ACG TTC GAA AAC GAG GCC GAT
CTG GAA CAG GTG ATT GAC ATC ATG CTG CCG CTC CGC ATC GGT ATG GCC CCG TTA CAG AAC GTT CCA GTG
TTG CGC AAC ATC TTC ATG GAT GCT GCG GCC GTG TCA CAT CGC GAT GAG TGG CAT GCT GGC CCG GGA CAT
CTG TCG GAC GAC GAA ATC AAG ACC ATG CAA CGC GAA CTG AAT CTG GGT TAC TGG AAT CTG TAT GCC AGT
GTG TAC GGT CCG CCT CCT CAG ATC GAA ATG TTC CTG GCC ATG ATC AAA GAA GCG TTC CTG CAA GTT CCG
GGA GCA CGC TTT GCT ACG ACC AAA GAT CGT CCC GAA AGT CCG GAA GAT CGC GGT GGT CAT GTG CTT CAC
GAT CGT CAC AAA ATC AAT CGT GGC ATT CCC ACC ATT GAA GAA CGC CAC CTG ATG GAT TGG GTC CCC AAT
```

GGT GGT CAC ACT AGC TTT TCT CCG GTA AGC GCG CCG GAC GGG AAA GAT GCG ATG CGC CAG GCG CTT ATG
GTG AAG AAA CGG GCA GAC GAG TTT GGC CAG GAT TAT GCC GCA CAA TTC ATT GTG GGG CTT CGG GAG ATG
CAT CAC ATC TGC CTG TTC CTG TAT AAT ACC GCG GTA CCG CGT GAG CGC GAT AAC ACC CTG GCT ATG GCA
CGC ATT TTG GTG GAA GAA GCC GCC GAT GCG GGC TAT GGG GAG TAT CGC ACT CAT CTG GCC TTA ATG GAC
CAG GTC ATG GCG ACG TTT GAT TAC AAT GAT GGC GCA TTA CTT CGC TTT CAC GAA CGT GTT AAA GAC GCC
CTG GAT CCC AAC TCC ATT ATG GCC CCG GGT AAA AGC GGC ATC TGG GGA GCA CGC TAC CGT GAT CGT GGC
CTC GAA TTA TTA CAT GCA CCC GCG TTA GAA GAT AGT ACC CCG AAT TCA GCG TAG

>WP_026846239 [*Geodermatophilaceae* bacterium URHA0031]

ATG AGC CGC ATC CTC CCT CCG GAA CTG AGC GAT AGC GAT TTT GAT GCC GCT ATT GCG CGC TTT CGC GAC
GTG GTT GGC GAG AAA CAT GTG CTT ACC GAG GAT GGG GAC CTG TCT CGG TAT CGG GAT CCG TAT CCG GTG
GGT GGG CAG CCT TCC GGA GGT GCA AGT GCA GCG GTC GCG CCC GAA ACG TCG GAA CAG GTA CAG GAA ATT
GTC CGC ATT GCG AAC GAG TAT GGG GTA CCC CTG AGT CCG ATT TCT ACC GGC AAG AAC AAC GGC TAC GGC
GGC GGT CAA CCA CGT CTG TCT GGT GCT GTT GTG GTT GAC ACC GGC CTG CGC ATG AAC CGC ATC CTG GAA
GTC AAT GAG AAG TTT GGC TAC GCG CTG CTT GAA CCA GGG GTT TCC TAC TTC GAC CTG TAC GAA CAC TTG
CAG GCG AAT GCC CCG TCC TTG ATG CTG GAT TGC CCG GAT CTG GGT TGG GGT AGT GTG GTT GGT AAT ACG
CTT GAT CGC GGT GTG GGC TAT ACC CCT TAT GGC GAT CAC TTG ATG TGG CAA ACA GGC CTG GAA GTA GTG
TTG CCC ACC GGC GAT GTC ATG CGT ACG GGT ATG GGT GCG GTT CCC GGT TCA AAC GCG TGG CAG CTG TTT
CCG TAC GGA TTC GGG CCG TTT CCG GAC GGG CTG TTT ACA CAG AGC AAC TTA GGT ATT GTG ACC AAA ATG
GGG ATT GCG CTT ATG CAA CGT CCA CCA GCC TCT GAG ACT TTC GTC ATT TCC TTC GAT CGC GAA GAG GAT
CTC GAA CAG GTA GTG GAC ATT ATG TTG CCG TTA CGC ATC AAC ATG GCA CCG CTT CAG AAT GTC CCG GTT
CTC CGC AAC ATC ATC TTG GAT GCC GGC GTT GTG AGC AAA CGC ACT GAG TGG CAT GAT GGT GAC GGC CCA
CTG CCG CCA GAA GCC ATT AGC CGT ATG AAA GCT GAA CTG GGC TTG GGC TAT TGG AAT CTG TAT GGA ACC
GTA TAC GGT CCT CCG CCA GTG CTG GAA GCT CAT CTG GGT ATC ATC AAA GAC GCG TTC TTA CAG GTG CCG
GGC AGT CGC TTT GCA ACG ACG CAG GAT CGT GAT GAA GCG ACT GAT CGT GGG GCT CAC GTC CTG CAT GAT
CGT CAC CGG ATT AAT AAC GGA ATT CCG TCA CTC GAC GAA ATG AAA CTG ATG GAA TTC GTA CCG AAT GGT
GGC CAT ATT GGC TTT AGC CCT GTC TCA GCA CCG GAT GGT GCC GAT GCG CTG CGT CAG TCG CAA ATG GTG
CGC CGT CGT GCG GAC GAA TAC CGC AAG GAT TAT GCC GCG CAA TTC ATC GTG GGC TTA CGT GAG ATG CAT
CAC ATT GGC CTC TTC CTG TTT GAC ACG ACC GAT GCC GTT GCC CGT CAG GAA ACA CTG GAC TTA GCT CGC
GTT CTG ATC GAT GAA GCT GCA GCA GCG GGG TAT GGC GAA TAT CGC ACC CAT AAC GCA CTG ATG GAC CAA
GTC ATG GGT ACC TAT AAT TGG GGA GGT GAC GCC TTA CTG AAA TTT CAC GAG ACT ATC AAA GAC GCC TTA
GAT CCG AAT TCG GTG ATG GCA CCT GGA AAA TCG GGC ATT TGG GGC CGC AAA TAC CGC GAT CGT GCC CTG
GCG TAG

>WP_106243419 [*Allonocardiopsis opalescens* DSM 45601]

ATG GTC CGG AAA TTG CCG CCA GGT TTG AAT GAA CAA TCG TTT GCG GAT GCG GTT GCA AGC TTT CGC CGT
GCG GTA GGG GAC AAA TGG GTT ATT GAC GAC GAT GCC CAA CTT GCC AAC TAC CGT GAC CCC TTT GCG GTG
CTC GAC CCG GAA CAC CTG ACG GCA AGT GCC GTA GTG ATG CCA GCC AGC GTG GAA GAA GTG CAG GCA GTT
TTA GCG GTT GCG AAC GAC ACT GGC GTG CCA CTG TCT CCG GTG TCC ATT GGT AAG AAC CTG GGC TAT GGT
GGG CCT GCA CCG CGC TTA CCC GGC GCG GTT GTC GTG GAT CTC AAA CGT ATG AAT CGC ATT CTT GAG GTG
AAC GAG AAG TTT GGT TAT GCT CTG GTA GAG CCG GGA GTT TCT TTC ATG GAG TTA GAC AAC TAT CTG CGC
GAA CGC GGG ATC GAT TTC TGG GTC GAT GTT CCG GAT CTG GGT TGG GGC AGC GTC TTA GGC AAT ACG CTG
GAA CGT GGA GTT GGC TAT ACA GCG TAT GGT GAT CAC TTT GCC ATC CAG TGC GGT ATG GAG GTG GTG CTG
GCG GAT GGC GAC GTC GTA CGC ACG GGA ATG GGC GGT GTA CCA GGC TCA AGC ACG GCT CAG CTG TTT AAG
TAC GGC TTT GGT CCG GTC TAT GAC GGC ATT TTC ACG CAG TCT AAC TTC GGC ATC GTC ACC AAA ATG GGT
CTG TGG CTG TTG CCG AAA CCG CCG GGC TAT CAA GCG TAC ATG ATT ACC CTG GCC CAT GAG GAA GAT TTG
GGG CCT TTC GTA GAA ATT CTC CGC ACC CTG AAG ATG AAT GGC ACC ATC ACC AAT GTG CCG TCA TTA CGC
AGT GTC TTG CTG GAT GCT GCA GCT GTT GCA CCA CGC TCT CAT TTC TAC AGT GGC ACT GGA CCC GTG CCC
GAT TCG GTA TCC CGC AAA ATT ATG GCG GAC CTG AAC ATT GGG TGG TGG AAC TTC TAT GGT GCG ATG TAT
GGC CCG CAA GCC TCG ATC GAT CTT CAG TGG CAG ACA GTT CGG GAT GCC TTT TCC ACC ATT CCT GGC GCG
AGC TTC TAC CTC GCT GGT GAA CAC GAT TCC CCA GTC TTG GCA ACT CGT GCT AAA GTG ATG GCG TGT CAA
CCG AAT CTG GAA ACC GCT GAC ATC TTT CAG TGG TTC GAC AAT GGA GGC CAT GTG GAT TTT GCC CCG CTG
AGT CCT GCG ACC AGC GAA GAT GCC CTG TCA CAG TAC GCA ATG GTT CGT GAT GCG TGC TTA GAG TTT GGG
AAA GAT TAC ATG GGC AAT TGG ATC GTG GGT CGT CGC GAA ATG CAT CAT ATC CAG ATG ACA ATG TTC GAT
ACC AAA GAC CCG GAT GAT CGC ACC CGC ACT CTG GCC TTT ACG AAA CAG CTG ATT CGT CAG GCC GCA GAA
CGT GGT TAC GGC GCC TAT CGC GCT CAC CCT GCA ATC ATG GAT GAA GTG GCG GCG ACC TTT TCG TTT AAC
GAT GGT GCG CTC ATG CGT CTG AGC GAA CGG GTC AAA GAC GCC CTG GAT CCG AAA GGG ATT CTG GCA CCG
GGT AAA CAA GGG ATT TGG CCG AAA AGC CTG CGT GGT AAA GGC CTT GCG TAG

>KAB1645308 [*Gulosibacter chungangensis* KCTC 13959]

ATG AAT TTT CGC ACG CTC CCG GAT GGG GTT AGT GCC GAG CAG TTC GCA AAT GCG ATA AGC GAG TTT AGT
GAA ACT ATC GGC AGC GAA TAC GTG CGC GTA GAT GAA GCC ACC GTC AGC GAA TAT GAT GAT AAA TTC CCC
GTC ACC GAC GGT GAT GAA TTC AAG GGC AGT GCG GTT ATC TGG CCG GGC AGC ACG GAA GAC GTT CAG GTG
ATT GTT CGT ATT GCC AAT AAA TAT GGC ATC CCG CTG CAC GCG TTC TCC GGC GGC CGG AAC CTC GGA TAC
GGT GGC TCA TCA CCC ATG CTC ACG GGG ACG GTC CTG CTC CAC TTG GGT AAG CGT ATG AAC CGT GTG CTC
GAA ATC AAC GAG AAG CTC GCC TAT GCC GTT GTC GAG CCG GGG GTG GAC TAC AAG ACC CTG TAT GAA GCG
GTT CGA GAC TCG GGT GCC AAA CTC ATG ATT GAC CCC GCC GAA CTC GAC TGG GGC AGC GTC ATG GGC AAC
ACC ATG GAG CAT GGT GTG GGC TAC ACC CCC TAT GCC GAC CAC TCG ATG TGG CGG TGC GGG ATG GAG GTA
GTC CTG GCT GAC GGG GAA GTG CTG CGG ACC GGT ATG GGC GGG CTA CCC GGC TCG GAA GCT TGG CAT CTC
TAC CCG GGC CAG TTG GGG CCA TCG ATT GAA GGG CTG TTC GAG CAG TCG AAC TTC GGT ATC TGC ACC CGC
ATG GGG ATG CAG CTC ATG CCG ACA CCT CCC GAG ATG CTG AGC TTC GCG ATC TAC TTC GAG AAC GAA GAC
GAT CTC CCG GCG ATC ATG GAG ACC ACG CTG CCG CTG CGG ATC GGC ATG GCA CCG CTT CAG GCG GCA CCG
ATC GTG CGA AAC GTT ACC TTC GAT GCC GCA TGC GTG TCG AAG CGT GAA GAG TGG CAG ACC GAG CCC GGC
CCG CTG ACT GAT GAA GCG AAG CAG CGC ATG GTG GAT GAA CTC GGT ATC GGG CAC TGG ATC GTG TAT GGC
ACC TGC TAC GGT CCT CGC TGG CAG ATC GAC AAG TAC ATC GAA ATG ATC CGT GAC GCC TAT TTG CAG ATC

CCC GGC GCG CGG TTC GAG ACG AAT GAG ACG CTC CCA CTT CGC GAG GGT GAT CGT GCG AGC GAG CTG CTG
AAC GCC CGT CAT GAA CTG AAC ACC GGT GTC CCG AAC CGT CAC TCC GCC GCG GTA TTC GAC TGG TTC CCG
AAC GCT GGC CAC TTC TTC TAC GCC CCG GTG TCA GCG CCC AGT GGT GAG GAC GCC GCG AAG CAA TAT GAA
GAT ACG AAG CGA ATC AGC GAC GAC CAC GGC ATC GAC TAT CTT GCG CAA TTC ATT ATT GGC CTG CGT GAG
ATG CAC CAC ATT TGT TTG CCG CTG TAT GAC ACC GCA GAC CCA GCA AGT CGT AAA GAA ACG CTC GAT ATG
ACG CGT GAG CTT ATT CGC GCT GGT GCT GAA GAA GGG TAT GGA ATC TAT CGA GCG CAC AAT GTA CTT GCG
GAT CAA GTT GCT GAA ACC TAT AGC TTT AAT AAC CAC ATT CAA CGT CGA TCC CAC GAG CGC ATT AAA GAT
GCG CTG GAC CCC AAT GGA ATT CTG AAC CCT GGA AAG TCG GGA ATC TGG CCG GAG CGA TTG CGC AAT AAA
TAA

Multiple Sequence alignment

Based on the translated protein sequence, using the standard codon usage for *E. coli*, a multiple sequence was calculated using MEGA 11.[5] The standard parameter in the program were used.

```
WP_106243419      -MVRKLPPGLNEQSFADAVASFRRAVGDKWVIDDDAQLANYRDPFAVLDPEHLTASAVVM 59
KAB1645308        MNFRTLPDGVSAEQFANAISEFSETIGSEYVRVDEATVSEYDDKFPVTDGDEFKGSAVIW 60
WP_071213834      -MSRIIPLDVSEADFDAALEEFRGALGAQWVLSSPVELEAFADPYPTTNGLEFLPGAVIS 59
WP_097207849      -MARLLPPGLSESDFDAAIARFRDVVGDKYVVTEDGDLARYRDPYPVGSEPATGASAAIS 59
WP_026846239      -MSRILPPELSDSDFDAAIARFRDVVGEKHVLTEDGDLSRYRDPYPVGGQPSGGASAAVA 59
WP_091045259      -MSRTLPPDVSEQDFARALDAFAAVVGEKWVISDPAELATYADPYPVGEGFGQAPSAVVS 59
WP_128378015      -MTRTLPPGVSDEDFTSALTAFRDVVGDEFVRTDEAELARFHDPYPVGDADAHLASAVIS 59
ABG95085          -MTRTLPPGVSDERFDAALQRFRDVVGDKWVLSTADELEAFRDPYPVGAAEANLPSAVVS 59
                     * :*  :.   *  *:  *  .:* : *      :  : * : .        .*.:

WP_106243419      PASVEEVQAVLAVANDTGVPLSPVSIGKNLGYGGPAPRLPGAVVVDL-KRMNRILEVNEK 118
KAB1645308        PGSTEDVQVIVRIANKYGIPLHAFSGGRNLGYGGSSPMLTGTVLLHLGKRMNRVLEINEK 120
WP_071213834      PSTPEEVQVIVGIANKYKVPLSPVSTGKNLGYGGAAPRLSGTVVVNTGERMNKIIEVNEK 119
WP_097207849      PESTEQVQEIVRIANEYGVPLSPISTGRNNGYGGGQPRLSGAVVVNTGERMNRIIEVNEK 119
WP_026846239      PETSEQVQEIVRIANEYGVPLSPISTGKNNGYGGGQPRLSGAVVVDTGLRMNRILEVNEK 119
WP_091045259      PADVEQVQAVVRIANEHGIPLMPISTGKNNGYGGASPRLAGSVVVNTGARMNRILEVNEK 119
WP_128378015      PRDTEQVQEVVRIANRYGIPLSVISTGRNNGYGGSAPRLSGAVVVNTGERMNRILEVDEK 119
ABG95085          PESTEQVQDIVRIANEYGIPLSPVSTGKNNGYGGAAPRLSGSVIVKTGERMNRILEVNEK 119
                  *   *:** :: :**   :**  .* *:* ****  * * *:*::.   ***:::*::**

WP_106243419      FGYALVEPGVSFMELDNYLRERGIDFWVDVPDLGWGSVLGNTLERGVGYTAYGDHFAIQC 178
KAB1645308        LAYAVVEPGVDYKTLYEAVRDSGAKLMIDPAELDWGSVMGNTMEHGVGYTPYADHSMWRC 180
WP_071213834      YAFALVEPGVTYFDLYNHIQEKGYNLWIDVPDLGWGSIVGNTLDRGVGYTPYGDHWSWQT 179
WP_097207849      YGYALLEPGVSYFDLYEYLEANAPSLMLDCPDLGWGSVVGNTLDRGVGYTPYGDHLMWQT 179
WP_026846239      FGYALLEPGVSYFDLYEHLQANAPSLMLDCPDLGWGSVVGNTLDRGVGYTPYGDHLMWQT 179
WP_091045259      YGYALVEPGVTYFDLYEYLEEHAPSLMLDCPDLGWGSVVGNTLDRGVGYTPYGDHLMWQT 179
WP_128378015      LGYALLEPGVTYFDLHEYLEAHAPSLMIDCPDLGWGSVVGNALDRGAGYTPYGDHFMWQT 179
ABG95085          YGYALLEPGVTYFDLYEYLQSHDSGLMLDCPDLGWGSVVGNTLDRGVGYTPYGDHFMWQT 179
                   .:*::**** :  * : :.     : :*  :*.***::**:::*.***  *.**   :

WP_106243419      GMEVVLADGDVVRTGMGGVPGSSTAQLFKYGFGPVYDGIFTQSNFGIVTKMGLWLLPKPP 238
KAB1645308        GMEVVLADGEVLRTGMGGLPGSEAWHLYPGQLGPSIEGLFEQSNFGICTRMGMQLMPTPP 240
WP_071213834      GLEVVLPDGDLLRTGMGAMSGSDAWQLFPYGFGPYPDGLFSQSNYGIVTKLGIALMPAPP 239
WP_097207849      GLEVVLPTGEVMRTGMGAVPGSTTWQLFQYGFGPFPDGLFTQSNLGIVTKMGIQLMQRPP 239
WP_026846239      GLEVVLPTGDVMRTGMGAVPGSNAWQLFPYGFGPFPDGLFTQSNLGIVTKMGIALMQRPP 239
WP_091045259      GLEVVLPQGEVMRTGMGAVPDSNTWQLFPYGFGPFPDGLFTQSNLGVVTKMGIALMQRPP 239
WP_128378015      GMEVVLPQGDVMRTGMGALPGSTTWQLIPYGFGPYPDGMFTQSNLGIVTKMGIALMQKPP 239
ABG95085          GLEVVLPQGEVMRTGMGALPGSDAWQLFPYGFGPFPDGMFTQSNLGIVTKMGIALMQRPP 239
                  *:****  *:::*****.: .* : :*    :**  :*:* *** *: *::*: *:  **

WP_106243419      GYQAYMITLAHEEDLGPFVEILRTLKMN-GTITNVPSLRSVLLDAAAVAPRSHFYSGTGP 297
KAB1645308        EMLSFAIYFENEDDLPAIMETTLPLRIGMAPLQAAPIVRNVTFDAACVSKREEWQTEPGP 300
WP_071213834      ASETFLITFENEADLEQVIDIMLPLRIGMAPLQNVPVLRNIFMDAAAVSHRDEWHAGPGH 299
WP_097207849      SSTTFLITFDREEDLAQVVDIMFPLRVNMAPLQNVPVLRNIVLDAGVVSKRTEWHDGDGP 299
WP_026846239      ASETFVISFDREEDLEQVVDIMLPLRINMAPLQNVPVLRNIILDAGVVSKRTEWHDGDGP 299
WP_091045259      ASATYLITFENESDLEQIVDTMLPLRINMAPIQNVPVIRNIILDAAAVSQRSEWYDGEGP 299
WP_128378015      ASMTYQITFENESDLEQIVDIMLPLRINMAPLQNVPVLRNIILDAAVVSQRADWYDGDGP 299
ABG95085          ASQSFLITFDKEEDLEQIVDIMLPLRINMAPLQNVPVLRNIFMDAAAVSKRTEWFDGDGP 299
                     :: * : .* **  .::    *::. . :  .* :*.: :**. *: * .:    *

WP_106243419      VPDSVSRKIMADLNIGWWNFYGAMYGPQASIDLQWQTVRDAFSTIPGASFYLAGEH---- 353
KAB1645308        LTDEAKQRMVDELGIGHWIVYGTCYGPRWQIDKYIEMIRDAYLQIPGARFETNETLPLRE 360
WP_071213834      LSDDEIKTMQRELNLGYWNLYASVYGPPPQIEMFLAMIKEAFLQVPGARFATTKDRPESP 359
WP_097207849      LPAEAIERMKSELGLGYWNLYGTVYGPPPVVEQYLGMIRDAFLQVPGSRFSTHHDRDE-A 358
WP_026846239      LPPEAISRMKAELGLGYWNLYGTVYGPPPVLEAHLGIIKDAFLQVPGSRFATTQDRDE-A 358
WP_091045259      LPAEAIERMKSELDLGYWNFYGTVYGPPPMIEMYLSMIKEAFLQIPGARFFTHEDRND-P 358
WP_128378015      LPPEAIERMKKELGLGYWNFYGTLYGPPQLIEMNYGIIKDAFGQIPGSRFQTHEERH--- 356
ABG95085          MPAEAIERMKKDLDLGFWNFYGTLYGPPPLIEMYYGMIKEAFGKIPGARFFTHEERD--- 356
                  :  .    :  :*.:* * .*.: ***   ::     :::*:  :**: *
```

```
WP_106243419      ---DSPVLATRAKVMACQPNLETADIFQWFDNGGHVDFAPLSPATSEDALSQYAMVRDAC 410
KAB1645308        GDRASELLNARHELNTGVPNRHSAAVFDWFPNAGHFFYAPVSAPSGEDAAKQYEDTKRIS 420
WP_071213834      EDRGGHVLHDRHKINRGIPTIEERHLMDWVPNGGHTSFSPVSAPDGKDAMRQALMVKKRA 419
WP_097207849      TDRGAHVLHDRHRINNGIPSLDEMKLLEFVPNGGHIGFSPISAPDGADALRQAQMVRQRA 418
WP_026846239      TDRGAHVLHDRHRINNGIPSLDEMKLMEFVPNGGHIGFSPVSAPDGADALRQSQMVRRRA 418
WP_091045259      ADRGAHVLHDRHKINNGRPSLDELAVLDFVPHGGHIGFSPVSAPEGRDAMRQAAMVKARA 418
WP_128378015      -DRGAHVLQDRHKINNGIPSLSEMKLMDWIPGAGHVGFSPISPPVGRDAMKQFRMVRSRA 415
ABG95085          -DRGGHVLQDRHKINNGIPSLDELQLLDWVPNGGHIGFSPVSAPDGREAMKQFEMVRNRA 415
                      . :*  * .:    *.     ::::.  .**  ::*:*   . :*  *   .:  .

WP_106243419      LEFGKDYMGNWIVGRREMHHIQMTMFDTKDPDDRTRTLAFTKQLIRQAAERGYGAYRAHP 470
KAB1645308        DDHGIDYLAQFIIGLREMHHICLPLYDTADPASRKETLDMTRELIRAGAEEGYGIYRAHN 480
WP_071213834      DEFGQDYAAQFIVGLREMHHICLFLYNTAVPRERDNTLAMARILVEEAADAGYGEYRTHL 479
WP_097207849      DEYRQDYAAQFVVGLREMHHIALLLFDTTKPEQRQRALDLARVLIDEAAAEGYGEYRTHN 478
WP_026846239      DEYRKDYAAQFIVGLREMHHIGLFLFDTTDAVARQETLDLARVLIDEAAAAGYGEYRTHN 478
WP_091045259      DEYVKDYAAQFIIGLREMHHICLFLYDTHDADARQETLDLTRLLIKEAAAEGYGEYRTHN 478
WP_128378015      DEYAKDYAAQFVVGLREMHHIALLLFDTQDATARNETLALTRLLIDEAAAEGYGEYRTHN 475
ABG95085          NEYNKDYAAQFIIGLREMHHVCLFIYDTAIPEAREEILQMTKVLVREAAEAGYGEYRTHN 475
                   :.  ** .::::* *****: : :::*     * . * ::: *:  .*  *** **:*

WP_106243419      AIMDEVAATFSFNDGALMRLSERVKDALDPKGILAPGKQGIWPKSLRGKGLA-------- 522
KAB1645308        VLADQVAETYSFNNHIQRRSHERIKDALDPNGILNPGKSGIWPERLRNK----------- 529
WP_071213834      ALMDQVMATFDYNDGALLRFHERVKDALDPNSIMAPGKSGIWGARYRDRGLELLHAPALE 539
WP_097207849      ALMDQVMGTYDWGDGALRRFHETIKDALDPNSIMAPGKSGIWGRKYRDKGLA-------- 530
WP_026846239      ALMDQVMGTYNWGGDALLKFHETIKDALDPNSVMAPGKSGIWGRKYRDRALA-------- 530
WP_091045259      ALMDDVMATFDWNDGALLKFHESIKDALDPNGVIAPGKSGVWPAKYRGRGL--------- 529
WP_128378015      ALMDQVMGTYNWGDGALLKFHEAIKDALDPNGIIAPGKSGVWPARYRGKGLA-------- 527
ABG95085          ALMDDVMATFNWGDGALLKFHEKIKDALDPNGIIAPGKSGIWSQRFRGQNL--------- 526
                  .: *:*  *:.:..    :  * :******:.:: ***.*:*    *.:

WP_106243419      ------- 522
KAB1645308        ------- 529
WP_071213834      DSTPNSA 546
WP_097207849      ------- 530
WP_026846239      ------- 530
WP_091045259      ------- 529
WP_128378015      ------- 527
ABG95085          ------- 526
```

Table S2. Sequence identity of selected 4-phenol oxidases towards the eugenol oxidase from *Rhodococcus jostii* RHA1 (*Rj*EUGO)

Enzyme	Accession number	Organism	Sequence identity to *Rj*EUGO
*Rj*EUGO	Q0SBK1	*Rhodococcus jostii* RHA1	100%
*N*spEUGO	WP_091045259	*Nocardioides* sp. YR527	76%
*Sc*EUGO	WP_128378015	*Streptomyces cavernae*	76%
*Gs*EUGO	WP_097207849	*Geodermatophilus sabuli* DSM 46844	73%
*Gb*EUGO	WP_026846239	*Geodermatophilaceae bacterium* URHA0031	73%
*A*spEUGO	WP_071213834	*Arthrobacter* sp. UCD-GKA	68%
*Gc*4EPO	KAB1645308	*Gulosibacter chungangensis* KCTC 13959	51%
*Ao*EUGO	WP_106243419	*Allonocardiopsis opalescens* DSM 45601	50%

Supplementary Methods 3: Initial enzyme characterization

Thermal stability and solvent tolerance

Thermal shift assay

Enzyme solutions of 5 µM concentration were prepared in 50 mM potassium phosphate buffer pH 7.5 containing a final SYPRO-Orange concentration of 5 x (according to commercial stock solution, ThermoFischer). Of this mixture, 25 µL were used for every replicate in a 96-well-qPCR-plate. A temperature gradient ranging from 20 to 90°C, with an increment of 3°C min^{-1} was applied while continuous detection was done using the FRET-channel of the qPCR-cycler.

No large differences were observed for the melting points of six of the tested proteins (Table S3). *Gb*EUGO was found to be the most stable one with a melting point of 65.5°C, followed by *Rj*EUGO, with a melting point of 64.0°C. *N*spEUGO was found to be the least stable one, with a melting point of 56.0°C. These results are in agreement with additional stability tests for the selected enzymes (Figure S13). Here, it was observed that *Gb*EUGO is also stable towards a broad spectrum of organic solvents (Figure S14).

Table S3. Melting points determined by thermal shift assay. Primary data is provided in Supplementary Data 5.

**Enzyme**	**Melting point [°C]**
ScEUGO	61.5
GbEUGO	65.5
RjEUGO	64.0
NspEUGO	56.0
AspEUGO	61.5
GcEUGO	58.5

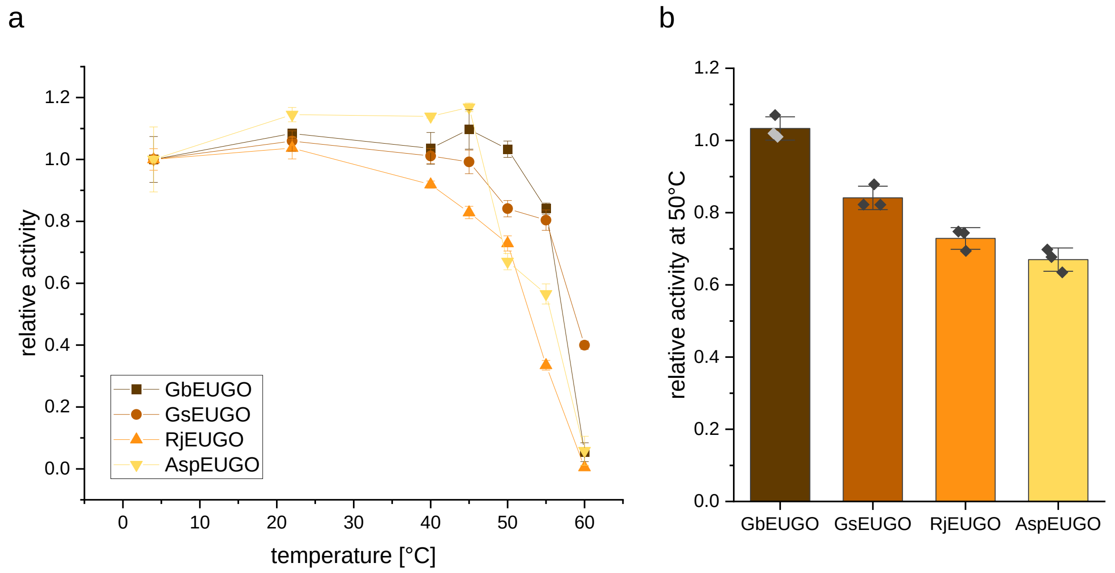

Figure S13. Temperature stability of selected EUGOs. **a** Relative activity of EUGOs from *Geodermatophilaceae bacterium, Geodermatophilus sabuli, Rhodococcus jostii RHA1* and *Arthrobacter* sp. after two-hour incubation at the respective temperature on 2 mM eugenol (**2**) in 50 mM potassium phosphate buffer pH 7.5. **b** Relative activity after two-hour incubation at 50°C for indicated EUGOs. All experiments were performed as triplicate and the standard deviation is shown as error bars.

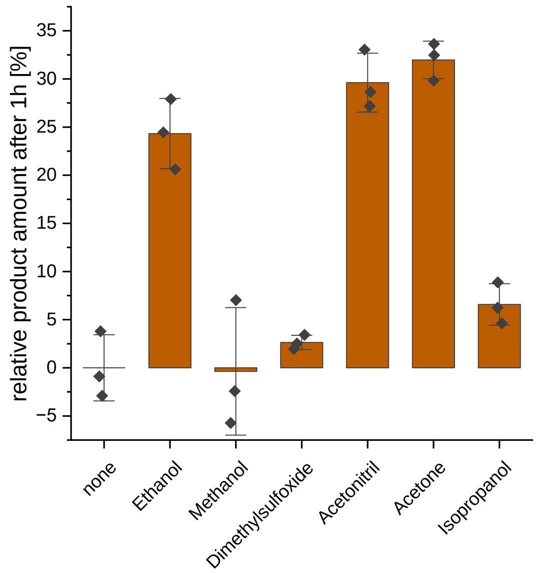

Figure S14. Solvent stability of *Gb*EUGO. Relative amount of coniferyl alcohol after two-hour reaction of *Gb*EUGO in 50 mM bis-Tris-propane buffer pH 9.5 with 2 mM eugenol (**2**) as substrate supplemented with 10 vol% of the indicated solvent. As reference a reaction without solvent was used. No solvent decreased the product amount significantly, but ethanol, acetonitrile and acetone were found to increase the product amount over 10%. All reactions were performed as triplicate and the standard deviation is shown as error bars.

Initial rates for substrate conversion

Xylenol orange assay for determination of initial rates

Initial rates were determined by colorimetric detection of hydrogen peroxide in the reaction solution as described in earlier studies.[6,7] Triplicates of 100 µL, containing a respective buffer (typically 50 mM potassium phosphate buffer pH 7.5) and 2.5 µM to 10 mM of substrate (typically 2 mM) shaken at 25°C in a 96-well-plate. For determination of tolerance towards solvents or ionic strength, additional substances were added in a respective amount. The reactions were started by the addition of a final enzyme concentration of 10 to 250 nM (typically 50 nM). After 3, 6, and 9 minutes, 20 µL of each reaction were transferred into a new 96-well-plate, already containing 180 µL of detection solution (250 µM $FeSO_4$, 25 mM H_2SO_4, 100 µM xylenol orange). After 30 min, the absorption at 560 nm (A_{560}) was measured and the initial rate was calculated from the linear slope through all three measurement points applying a hydrogen peroxide calibration curve (see Supplementary Data 4). For fitting of Michaelis-Menten curves please refer to section Supplementary Equations 1 to 4.

Table S4. Initial rate [s^{-1}] of selected enzymes on the indicated substrate. The rate was determined by xylenol orange assay in 50 mM potassium phosphate buffer pH 7.5 wit 2 mM of the respective substrate at 25°C. Errors represent the standard deviation of a triplicate measurement. The cells are colored in a gradient from highest (blue) to lowest (light orange) rate. Product formation was validated by GC-MS. The measured substrates and detected products are listed in Table S5.

No	*Sc*EUGO	*Gs*EUGO	*Rj*EUGO	*N*spEUGO	*A*spEUGO	*Gb*EUGO	*Ao*EUGO	*Gc*4EPO
**1**	0.48 ± 0.29	5.30 ± 0.36	1.60 ± 0.41	3.24 ± 0.06	1.67 ± 0.06	5.08 ± 0.13	n.d.	n.d.
**2**	5.25 ± 0.64	5.63 ± 0.05	4.77 ± 0.14	3.73 ± 0.08	1.40 ± 0.06	4.41 ± 0.15	2.99 ± 0.50	3.91 ± 0.08
**3**	1.21 ± 0.09	1.42 ± 0.05	0.39 ± 0.04	0.26 ± 0.03	0.14 ± 0.06	0.34 ± 0.03	0.23 ± 0.02	n.d.
**4**	0.30 ± 0.03	0.29 ± 0.03	0.62 ± 0.03	1.35 ± 0.03	0.70 ± 0.12	0.37 ± 0.01	2.63 ± 0.04	0.03 ± 0.02
**5**	2.93 ± 0.20	3.12 ± 0.02	4.77 ± 0.15	2.82 ± 0.01	2.14 ± 0.13	3.08 ± 0.12	3.75 ± 0.06	5.78 ± 0.25
**6**	2.64 ± 0.18	2.16 ± 0.11	0.25 ± 0.02	n.d.	n.d.	0.07 ± 0.01	0.10 ± 0.09	n.d.
**7**	1.12 ± 0.11	1.00 ± 0.07	1.92 ± 0.07	n.d.	1.44 ± 0.04	0.57 ± 0.17	2.84 ± 0.12	6.37 ± 0.03
**8**	0.07 ± 0.01	0.78 ± 0.05	1.41 ± 0.09	0.88 ± 0.01	0.25 ± 0.12	0.75 ± 0.07	0.68 ± 0.03	2.29 ± 0.10
**9**	0.33 ± 0.02	1.64 ± 0.03	2.66 ± 0.13	n.d.	0.99 ± 0.03	1.74 ± 0.10	2.13 ± 0.10	3.66 ± 0.12
**10**	0.03 ± 0.01	n.d.	n.d.	n.d.	n.d.	n.d.	n.d.	n.d.
**11**	0.11 ± 0.02	0.15 ± 0.05	0.06 ± 0.01	0.28 ± 0.09	0.13 ± 0.04	0.13 ± 0.02	n.d.	1.03 ± 0.06
**12**	1.50 ± 0.09	0.22 ± 0.06	0.08 ± 0.02	0.33 ± 0.05	n.d.	0.13 ± 0.05	0.33 ± 0.04	0.07 ± 0.01
**13**	0.10 ± 0.08	0.35 ± 0.07	0.11 ± 0.03	0.50 ± 0.03	n.d.	0.34 ± 0.05	n.d.	n.d.
**14**	n.d.	n.d.	n.d.	n.d.	n.d.	n.d.	n.d.	n.d.
**15**	n.d.	n.d.	n.d.	0.09 ± 0.04	0.05 ± 0.03	n.d.	0.10 ± 0.09	0.10 ± 0.02
**16**	0.27 ± 0.01	n.d.	0.44 ± 0.04	0.06 ± 0.03	0.14 ± 0.02	0.16 ± 0.03	0.40 ± 0.04	0.13 ± 0.02
**17**	n.d.	n.d.	n.d.	n.d.	0.05 ± 0.03	n.d.	n.d.	0.37 ± 0.04
**18**	n.d.	0.08 ± 0.07	n.d.	n.d.	n.d.	0.05 ± 0.04	n.d.	0.16 ± 0.02
**19**	n.d.	n.d.	n.d.	n.d.	n.d.	0.05 ± 0.01	n.d.	n.d.
**20**	n.d.	n.d.	n.d.	0.09 ± 0.05	n.d.	0.03 ± 0.02	n.d.	0.12 ± 0.02
**21**	n.d.	0.06 ± 0.02	0.07 ± 0.03	n.d.	0.05 ± 0.01	0.14 ± 0.09	0.16 ± 0.08	0.07 ± 0.03
**22**	0.21 ± 0.04	0.20 ± 0.12	0.44 ± 0.02	n.d.	n.d.	0.41 ± 0.07	n.d.	n.d.
**23**	0.24 ± 0.11	0.19 ± 0.10	0.48 ± 0.07	0.09 ± 0.08	0.16 ± 0.05	0.22 ± 0.01	n.d.	0.06 ± 0.02
**24**	n.d.	n.d.	0.03 ± 0.01	0.04 ± 0.02	n.d.	n.d.	n.d.	n.d.
**25**	0.34 ± 0.20	1.10 ± 0.04	0.81 ± 0.13	0.07 ± 0.03	0.51 ± 0.10	0.53 ± 0.05	0.58 ± 0.07	2.96 ± 0.02
**26**	n.d.	n.d.	n.d.	n.d.	n.d.	0.06 ± 0.04	n.d.	1.42 ± 0.03
**27**	0.09 ± 0.07	n.d.	0.22 ± 0.10	n.d.	n.d.	n.d.	n.d.	n.d.
**28**	n.d.	n.d.	n.d.	n.d.	n.d.	n.d.	n.d.	n.d.
**29**	n.d.	n.d.	n.d.	n.d.	n.d.	n.d.	n.d.	n.d.
**30**	n.d.	n.d.	0.06 ± 0.04	0.08 ± 0.04	n.d.	n.d.	n.d.	n.d.
**31**	n.d.	n.d.	n.d.	n.d.	n.d.	n.d.	n.d.	n.d.
**32**	n.d.	n.d.	n.d.	n.d.	n.d.	n.d.	n.d.	4.58 ± 0.18
**33**	0.44 ± 0.09	0.39 ± 0.18	0.63 ± 0.11	0.11 ± 0.05	0.48 ± 0.09	0.22 ± 0.08	0.27 ± 0.07	1.10 ± 0.08
**34**	0.02 ± 0.01	n.d.	n.d.	0.05 ± 0.01	0.06 ± 0.04	0.05 ± 0.04	n.d.	2.38 ± 0.07
**35**	n.d.	n.d.	n.d.	0.03 ± 0.02	n.d.	n.d.	n.d.	n.d.
**36**	n.d.	n.d.	n.d.	0.09 ± 0.05	n.d.	n.d.	n.d.	n.d.
**37**	n.d.	n.d.	n.d.	0.10 ± 0.01	n.d.	n.d.	n.d.	3.42 ± 0.1
**38**	0.07 ± 0.03	n.d.	n.d.	n.d.	n.d.	0.05 ± 0.04	n.d.	0.39 ± 0.04
**39**	0.09 ± 0.05	n.d.	0.08 ± 0.02	n.d.	0.10 ± 0.02	0.10 ± 0.04	n.d.	0.10 ± 0.05
**40**	0.06 ± 0.03	0.02 ± 0.01	n.d.	n.d.	n.d.	0.09 ± 0.06	n.d.	n.d.
**41**	n.m.	n.m.	n.m.	n.m.	n.m.	n.m.	n.m.	0.19 ± 0.02
**42**	0.34 ± 0.02	0.48 ± 0.09	1.95 ± 0.20	0.20 ± 0.04	n.d.	0.60 ± 0.01	0.90 ± 0.03	0.60 ± 0.03
**43**	n.d.	0.04 ± 0.03	0.03 ± 0.02	n.d.	n.d.	0.04 ± 0.03	n.d.	n.d.
**44**	0.04 ± 0.01	0.09 ± 0.02	0.08 ± 0.01	0.12 ± 0.01	0.04 ± 0.01	n.d.	n.d.	2.88 ± 0.18
**45**	0.03 ± 0.02	n.d.	0.06 ± 0.01	n.d.	n.d.	n.d.	n.d.	0.61 ± 0.07
**46**	n.d.	n.d.	n.d.	n.d.	n.d.	n.d.	n.d.	n.d.

n.d. = not detected; n.m. = not measured

Substrate overview and products detected by GC-MS

GC-MS measurements

200 to 400 µL of the reaction mixture were extracted with an equal amount of ethyl acetate in by vortexing. For phase separation, the tubes were centrifuged (5 min at 17,000 x g). The ethyl acetate phase was dried over anhydrous $MgSO_4$, before being vortexed and centrifuged again. Afterwards, 100 µL were transferred into a glass vial containing an inset and closed via a septum cap. Measurement was performed on a Shimadzu GCMS-QP2020 NX equipped with a FS-Supreme 5ms column (length: 30 m, id: 0.25 mm, od: 0.36 mm, thickness: 0.25 µm) from CS-Chromatographie Service GmbH was used. Either a temperature gradient ranging from 150°C to 200°C with 5°C min^{-1} increase, and 5 min hold time at the beginning and the end, or a temperature gradient from 150°C to 250°C with a temperature increase of 10°C min^{-1} and a hold time of 2.5 min at the beginning and the end was applied. Compounds were identified by NIST2017 library search.

Table S5. Overview of the substrates tested in this study and the products detected by GC-MS after enzymatic conversion. For initial rates of wildtype enzymes see table S2, and for enzyme variants see Table S6.

No	Name	Substrate	Main product	Side product(s)
**1**	Chavicol	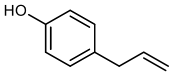	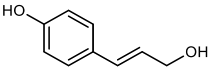	n.d.
**2**	Eugenol	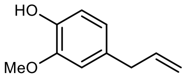	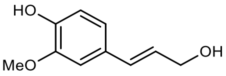	n.d.
**3**	4-Allyl-2,6-dimethoxyphenol	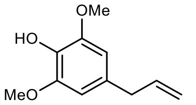	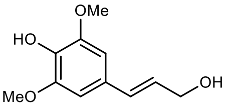	n.d.
**4**	4-Hydroxybenzyl alcohol	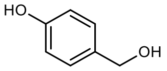	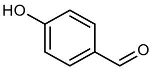	n.d.
**5**	Vanillyl alcohol	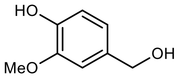	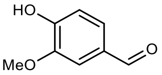	n.d.
**6**	4-Hydroxy-3,5-dimethoxybenzyl alcohol	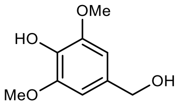	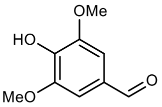	n.d.
**7**	3,4-Dihydroxybenzyl alcohol	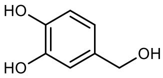	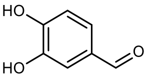	n.d.
**8**	3-Bromo-4-hydroxybenzyl alcohol	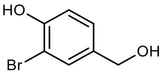	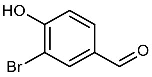	n.d.
**9**	4-(1-Hydroxyethyl)phenol	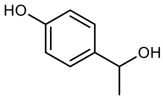	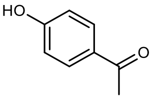	n.d.
**10**	4-Aminobenzyl alcohol	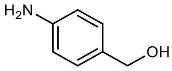	n.d.	n.d.
**11**	4-Methoxymethylphenol	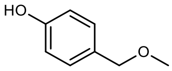	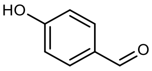	n.d.
**12**	Ethyl vanillyl ether	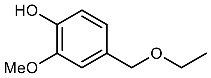	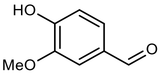	n.d.
**13**	Butyl vanillyl ether	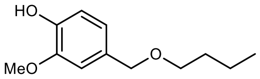	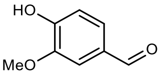	n.d.
**14**	4-(Aminomethyl)phenol	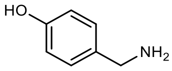	n.d.	n.d.
**15**	4-(1-Aminoethyl)phenol	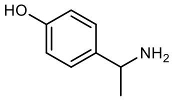	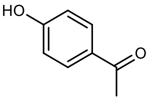	n.d.
**16**	Vanillyl amine	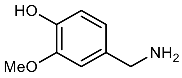	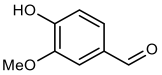	n.d.
**17**	Tyrosol	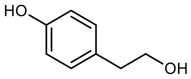	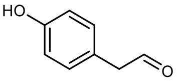	n.d.

Table S3 continued

No	Name	Substrate	Main product	Side product(s)
**18**	4-(2-Methoxyethyl)phenol	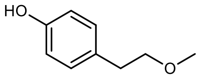	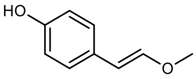	n.d.
**19**	Tyramin	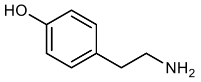	n.d.	n.d.
**20**	4-(2-Bromoethyl)phenol[2]	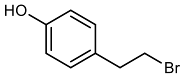	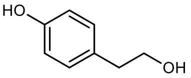	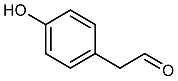
**21**	Hydroxytyrosol	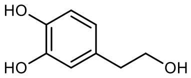	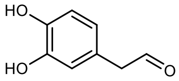	n.d.
**22**	3-Hydroxytyramine	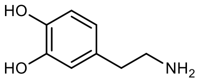	n.d.	n.d.
**23**	4-Hydroxy-3-methoxyphenethylamine	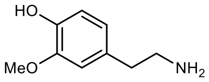	n.d.	n.d.
**24**	*p*-Cresol	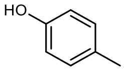	n.d.	n.d.
**25**	4-Methylcatechol	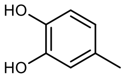	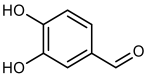	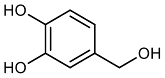
**26**	2-Methoxy-4-methylphenol	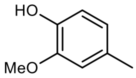	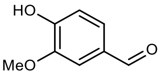	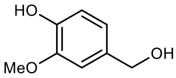
**27**	2-Amino-4-methylphenol	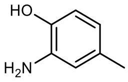	n.d.	n.d.
**28**	2-Chloro-4-methylphenol	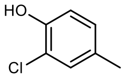	n.d.	n.d.
**29**	2-Brom-4-methylphenol	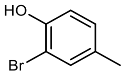	n.d.	n.d.
**30**	*p*-Toluidine	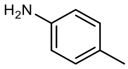	n.d.	n.d.
**31**	*p*-Toluenethiol	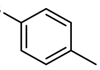	n.d.	n.d.
**32**	4-Ethylphenol	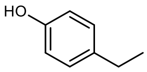	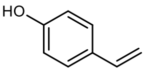	n.d.
**33**	4-Ethylcatechol[1]	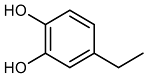	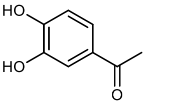	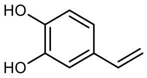 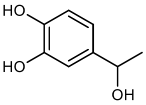
**34**	4-Ethylguaiacol[1]	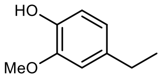	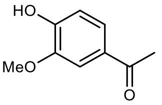	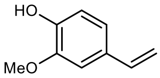

Table S3 continued

No	Name	Substrate	Main product	Side product(s)
**35**	4-Ethylaniline	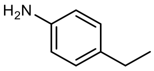	n.d.	n.d.
**36**	4-Ethylthiophenol	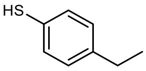	n.d.	n.d.
**37**	4-Propylphenol	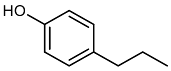	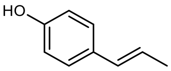	n.d.
**38**	4-Butylphenol	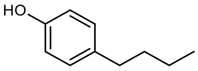	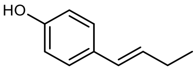	n.d.
**39**	4-Isopropylphenol	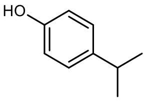	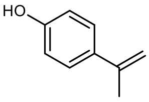	n.d.
**40**	4-*sec*-Butylphenol	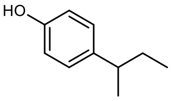	n.d.	n.d.
**41**	2-Methoxy-4-propylphenol	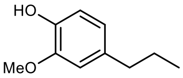	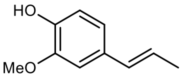	n.d.
**42**	4-Cyclopentylphenol	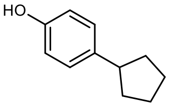	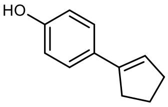	n.d.
**43**	4-Cyclohexylphenol	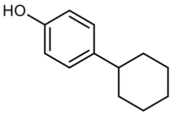	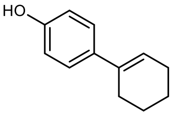	n.d.
**44**	5-Hydroxyindan	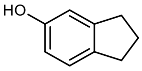	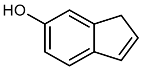	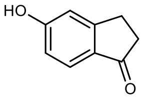
**45**	5,6,7,8-Tetrahydro-2-naphtol	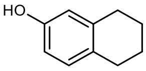	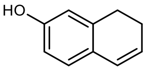	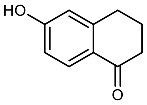
**46**	4-Hydroxybenzyl cyanide	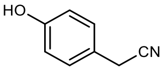	n.d.	n.d.

[1]For *Gc*4EPO, the dehydrogenated product was the main product; [2]Substrate **20** hydrolyses to **17** in aqueous medium, which then converted; n.d. = not detected

Supplementary Methods 4: Substrate binding in 4-phenol oxidases

Homology modelling

The structures of the eugenol oxidase from *Rhodococcus jostii* RHA1 (PDB: 5FXP and 5FXE), the 4-ethylphenol oxidase from *Gulosibacter chungangensis* (PDB: 7BPI), the vanillyl alcohol oxidase from *Penicillium simplicissimum* (PDB: 2VAO) and the *p*-cresol methyl hydroxylase from *Pseudomonas putida* (PDB: 1WVE) were applied as template using the Yasara software.[8] Homology models were docked with eugenol as substrate and refined by molecular dynamics simulation (10 ns, YASARA2-force field, water-filled periodic simulation box 5 Å around the structure). The structures were inspected using PyMOl.[9]

Autodocking of putative substrates

Autodocking experiments were performed using YASARA`s dock_runscreening macro, for which the VINA program was used.[10] Ligand-receptor complexes of interest were refined by molecular dynamics simulation for 10 ns.

Substrate binding in *Ao*EUGO

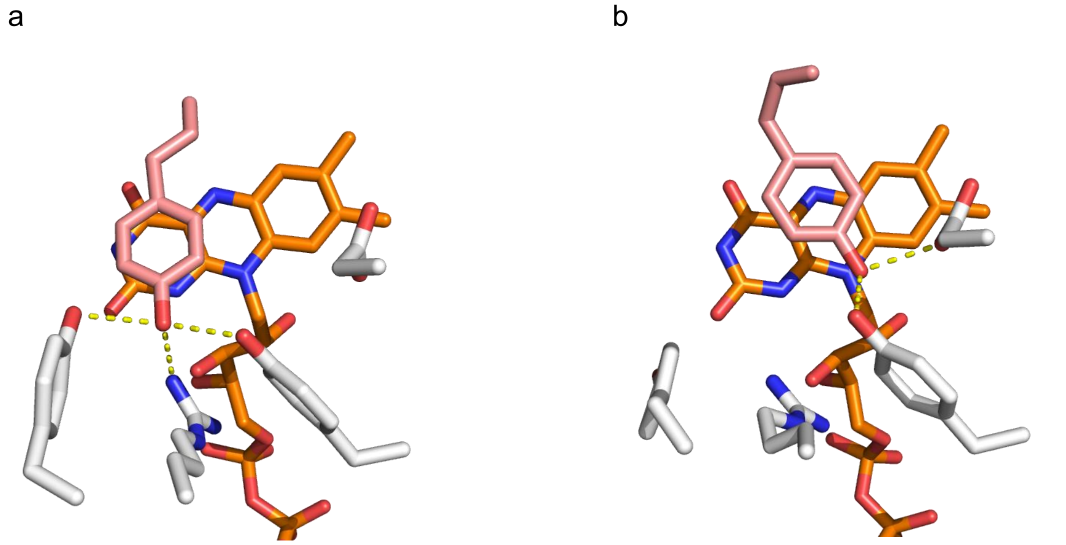

Figure S15. Catalytic center of the homology model of *Ao*EUGO bound to chavicol (**1**) before (**a**) and after 25 ns of MD simulation (**b**). The FAD cofactor is shown in orange and the substrate chavicol (**1**) in light red. Polar interaction between the substrates and the amino acid residues are depicted as dashed, yellow lines. The residues of the P-cluster, Y91, Y466 and R467, and Glu387 are depicted as sticks. The substrate rotates in the simulation and the phenolate is bound by Glu387 and Tyr91.

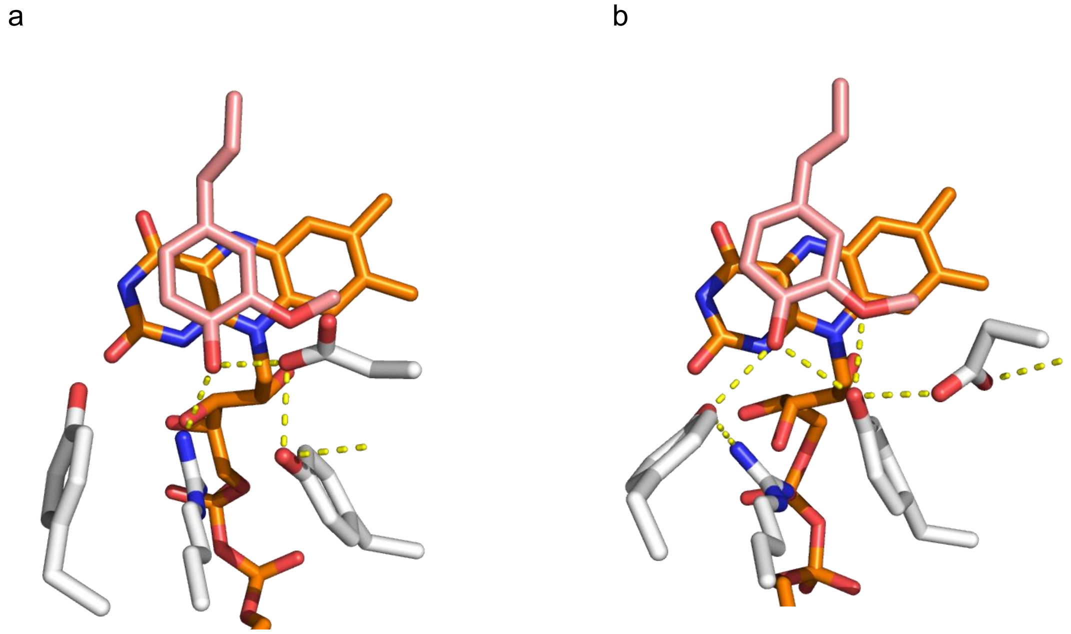

Figure S16. Catalytic center of the homology model of *Ao*EUGO bound to eugenol (**2**) before (**a**) and after 25 ns of MD simulation (**b**). The FAD cofactor is shown in orange and the substrate eugenol (**2**) in light red. Polar interaction between the substrates and the amino acid residues are depicted as dashed, yellow lines. The residues of the P-cluster, Y91, Y466 and R467, and Glu387 are depicted as sticks. The *o*-methoxy group of 2 prevents substrate rotation.

Substrate binding in *Asp*EUGO

a b

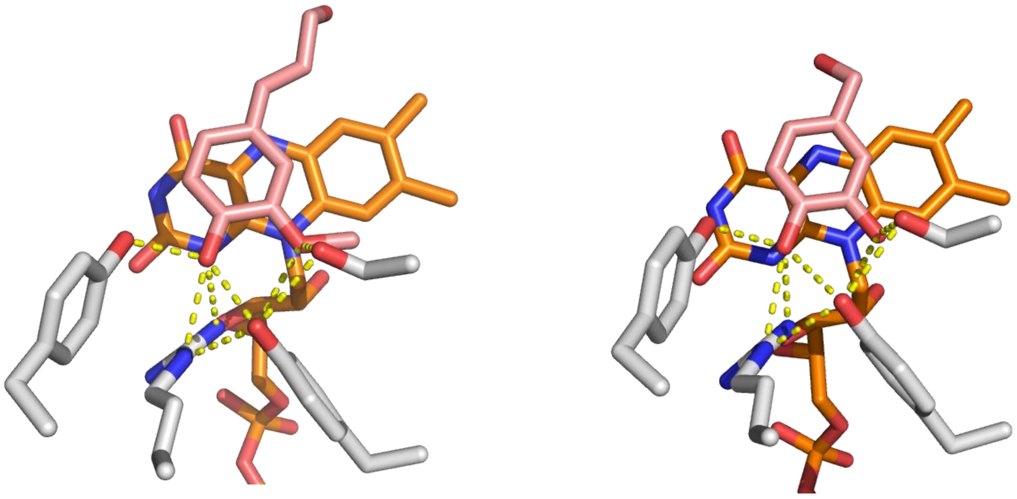

Figure S17. Autodocking of the homology model of *A*spEUGO with the product from eugenol conversion coniferyl alcohol (**a**) and 3,4-dihydroxybenzyl alcohol (**7**, **b**). The FAD cofactor is shown in orange and the respective substrate in light red. Residues of the P-cluster, Y91, Y475 and R476, are highlighted as well as S396. Polar interactions are depicted as dashed, yellow lines.

Supplementary Methods 5: Buffer optimization

pH dependent enzyme stability

Enzyme solutions of 1 µM concentration were incubated for 16 h in Britton-Robinson buffer at respective pH values, before the activity was measured uniformly in potassium phosphate buffer pH 7.5 using 2 mM eugenol as substrate by the xylenol orange assay (see Supplementary Methods 3). A final enzyme concentration of 50 nM was used, and the new pH value was adopted by dilution of the enzyme stock.

Buffer dependent enzyme activity

Initial rates were determined by the xylenol orange assay (see Supplementary Methods 3) using 50 nM of enzyme and 2 mM of eugenol in 50 mM of varying buffers. Buffers were selected according to the pH optimum of the respective enzyme.

Buffer dependent enzyme stability

In a 96-well plate, 200 µL reaction solution were placed containing 5 nM enzyme and 10 mM vanillyl alcohol as substrate in an indicated buffer. The plate was covered with transparent foil to avoid evaporation and was incubated for 16 h at 30°C shaking at 750 rpm in a Tecan plate reader. In 15 min intervals, the absorption of the product vanillin was measured at 350 nm. The vanillin concentration was calculated from a calibration curve (see Supplementary Data 4) and was divided by the applied enzyme concentration to calculate the conversions per active site of enzyme (turnover). Over the course of the reaction, maximal 0.5 mM vanillin were produced (5% conversion) to assure constant conditions for the catalyst during the reaction time.

Temperature dependent enzyme stability

Enzyme solutions of 1 µM concentration were incubated for 2 h in an indicated buffer in a temperature range between 4 to 60°C, before the activity was measured by the xylenol orange assay (see Supplementary Methods 3) uniformly using 2 mM eugenol as substrate in potassium phosphate buffer pH 7.5.

Comparison of *A*spEUGO and *Gb*EUGO

To determine the influence of Ser392 on the substrate acceptance, *A*spEUGO was compared in detail to the closest homolog available, *Gb*EUGO. Both oxidases share a sequence similarity of 79% but differ in the Ser/Gly in position 392 of the catalytic pocket. To investigate the enzymes at their respective optimal conditions, pH and buffer screening was conducted. This approach was chosen over the introduction of point mutations, as the rest of the protein is likely adapted to the altered function as well. Initial rates for the conversion of eugenol (**2**) of both enzymes reached a maximum at pH 9.5, which is comparable to other EUGOs (Figure S18).[2,3] *A*spEUGO reached a 2.5-fold higher rate at pH 9.5 compared to pH 7.5 while a less than 1.5-fold increase was found for *Gb*EUGO. On the contrary, changes in the reaction buffer had a larger impact on the performance of *Gb*EUGO (Figure S19) which is probably linked to the enzyme's lower tolerance for ionic strength (Figure S20). In the end, CHAPS buffer at pH 9.5 was identified to work best for *A*spEUGO while bis-Tris-propane at pH 9.5 was identified as optimal for *Gb*EUGO (Figure S21). Subsequently, kinetic parameters were determined for both enzymes in their respective buffer conditions (see main text).

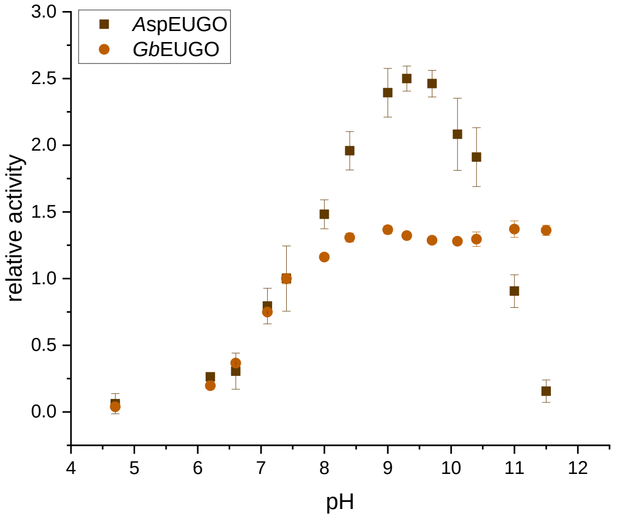

Figure S18. Relative activity of *A*spEUGO and *Gb*EUGO in dependency of the pH value. The initial activity was determined by xylenol orange assay in Britton-Robinson buffer at the indicated pH using a final concentration of 50 nM enzyme and 2 mM eugenol (**2**) as substrate. The reaction at pH 7.5 was used as reference value for both enzymes. All rates were determined as a triplicate and the standard deviation is shown as error bar.

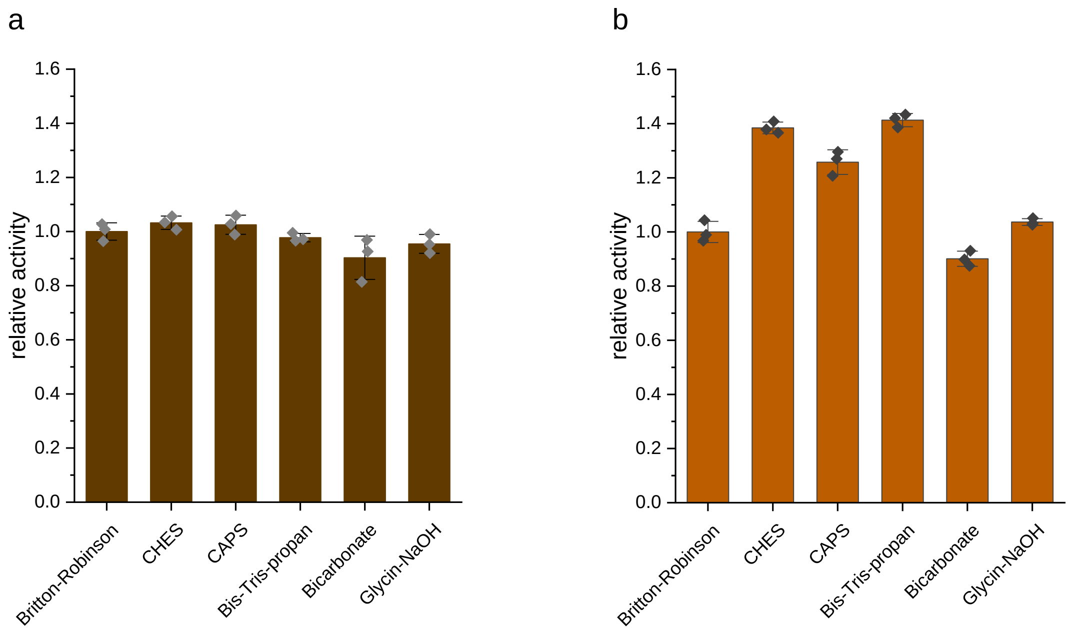

Figure S19. Relative activity of *A*spEUGO (**a**) and *Gb*EUGO (**b**) in dependency of the buffer. The initial activity was determined by xylenol orange assay in the indicated buffer using a final concentration of 50 nM enzyme and 2 mM eugenol (**2**) as substrate. The reaction in Britton-Robinson buffer pH 9.5 was used as reference value for both enzymes. All rates were determined as a triplicate and the standard deviation is shown as error bar. All buffers were adjusted to pH 9.5.

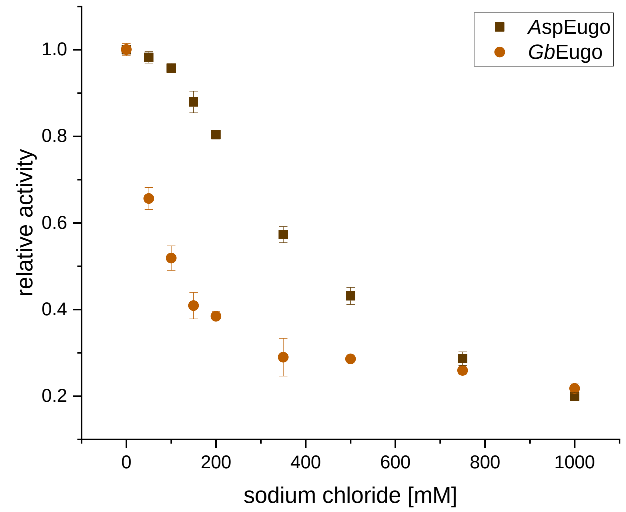

Figure S20. Relative activity of *A*spEUGO and *Gb*EUGO in dependency of the ionic strength. The initial activity was determined by xylenol orange assay in CHAPS buffer pH 9.5 for *A*spEUGO and bis-Tris-propane buffer pH 9.5 for *Gb*EUGO. A final concentration of 50 nM enzyme and 2 mM eugenol (**2**) as substrate were used. The reactions were performed in presence of the indicated amount of sodium chloride while the condition without additional salt was used as reference value for both enzymes. All rates were determined as a triplicate and the standard deviation is shown as error bar.

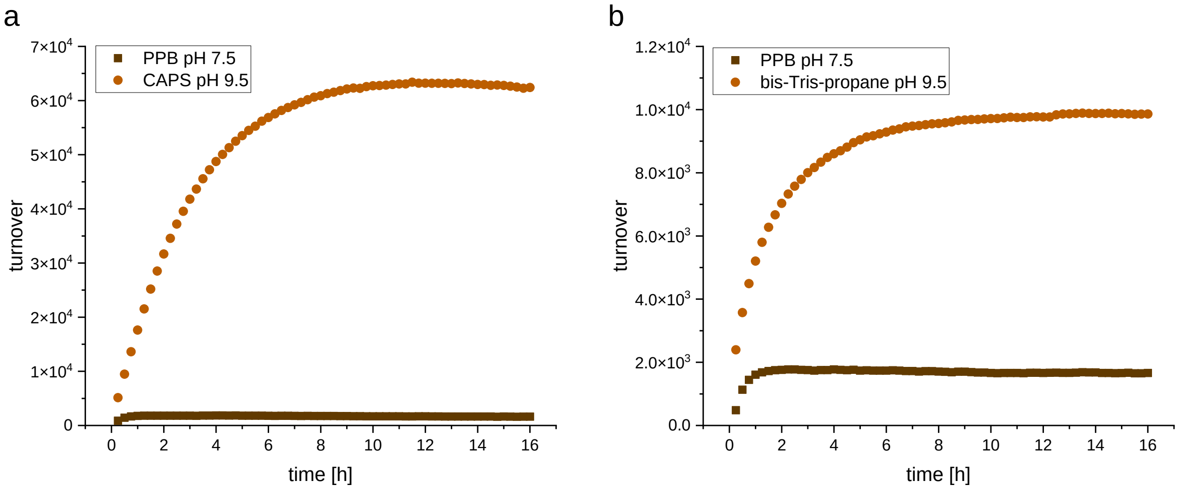

Figure S21. Conversions per active site of *A*spEUGO (**a**) and *Gb*EUGO (**b**) over time. The reaction was performed in the indicated buffer using a final concentration of 5 nM enzyme and 10 mM vanillyl alcohol as substrate. The concentration of the product vanillin was measured at 350 nm and was used to calculate the conversions per active site by division by the enzyme concentration (turnover).

Supplementary Methods 6: Michaelis-Menten kinetics

Michaelis-Menten kinetics were determined by the xylenol orange assay (see supplementary methods 3). The given substrate was prepared in differing concentrations ranging from 2.5 µM to 10 mM in the indicated buffer. The reaction was started by the addition of a respective amount of enzyme ranging between 15 to 250 nM. For *Gc*4EPO and for *Sc*EUGO variants, the curves were fitted according to the classical Michaelis-Menten equation, where v_{max} represents the maximum velocity, K_M the substrate concentration [S] at a velocity v of half v_{max}.

$$v = \frac{v_{max}[S]}{K_M + [S]} \tag{1}$$

For *A*spEUGO and *Gb*EUGO, additional effects were observed. For fitting of cooperative effects, the Hill equation was used where the additional parameter n describes the degree of cooperativity.[11]

$$v = \frac{v_{max}}{1 + \frac{K^n}{[S]^n}} \tag{2}$$

For *A*spEUGO, we further observed substrate inhibition which was described according to Haldane. Here, K_i represents the inhibition constant.[12]

$$v = \frac{v_{max}}{1 + \frac{K}{[S]} + \frac{[S]}{K_i}} \tag{3}$$

As for *A*spEUGO cooperativity and inhibition were observed at the same time, a model according to LiCata was applied which combines Hill and Haldane equation.[13] For simplicity, we assume that there is a single inhibition side per enzyme and the inhibition complex is catalytically inactive.

$$v = \frac{v_{max}}{1 + \frac{K^n}{[S]^n} + \frac{[S]}{K_i}} \tag{4}$$

For comparability between enzymes, v_{max} was transformed into k_{cat} by the following relation, where E_0 represents the enzyme concentration.

$$\frac{v_{max}}{E_0} \tag{5}$$

Michaelis-Menten kinetics of *A*spEUGO and *Gb*EUGO

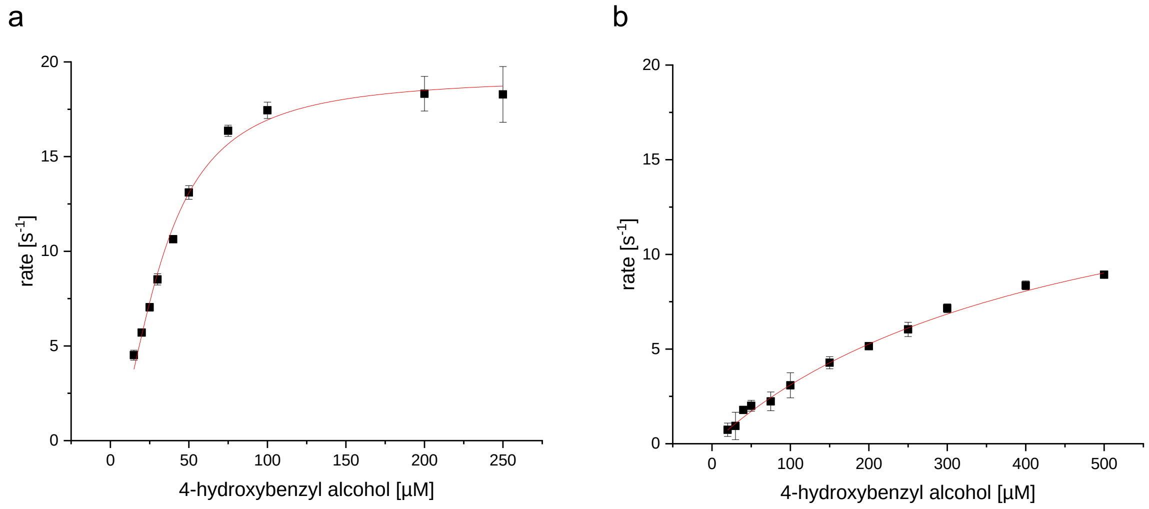

Figure S22. Michaelis-Menten kinetics of *A*spEUGO (**a**) and *Gb*EUGO (**b**) on 4-hydroxybenzyl alcohol (**4**) as substrate. The initial rates were determined by xylenol orange assay in CHAPS buffer pH 9.5 for *A*spEUGO and bis-Tris-propane buffer pH 9.5 for *Gb*EUGO. A final concentration of 50 mM buffer and 50 nM enzyme were used. The curves were fitted according to Hill. All reactions were performed as triplicate and the standard deviation is shown as error bars. Kinetic parameters are summarized in Table S6.

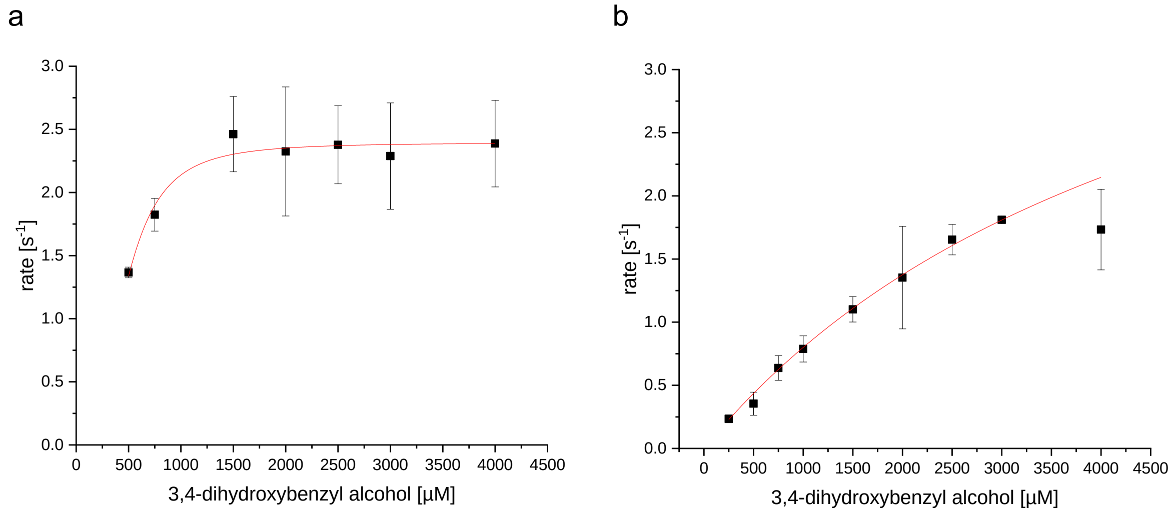

Figure S23. Michaelis-Menten kinetics of *A*spEUGO (**a**) and *Gb*EUGO (**b**) on 3,4-dihydroxybenzyl alcohol (**7**) as substrate. The initial rates were determined by xylenol orange assay in CHAPS buffer pH 9.5 for *A*spEUGO and bis-Tris-propane buffer pH 9.5 for *Gb*EUGO. A final concentration of 50 mM buffer and 50 nM enzyme were used. The curves were fitted according to Hill. All reactions were performed as triplicate and the standard deviation is shown as error bars. Kinetic parameters are summarized in Table S6.

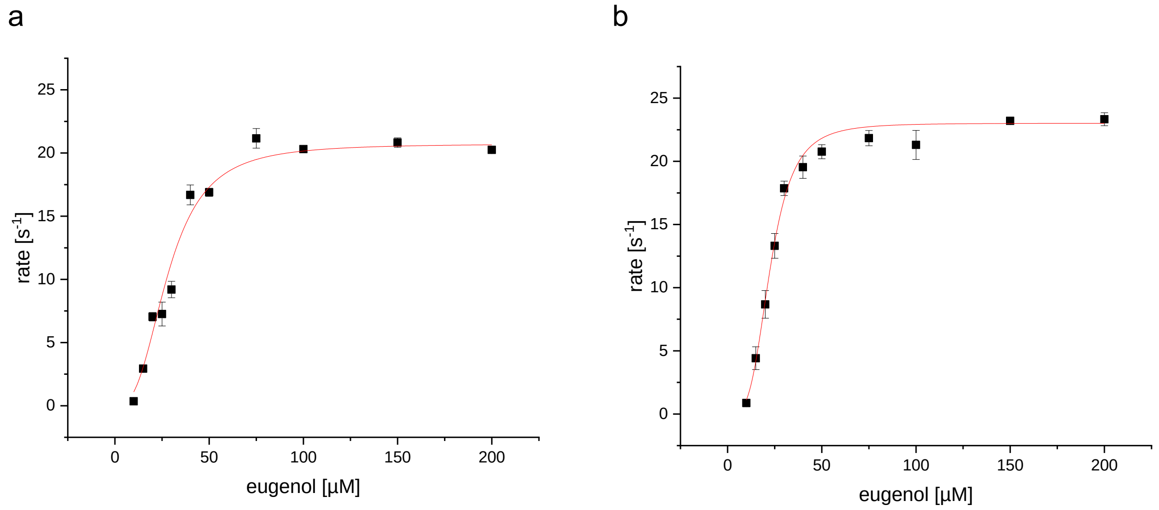

Figure S24. Michaelis-Menten kinetics of *Asp*EUGO (**a**) and *Gb*EUGO (**b**) on eugenol (**2**) as substrate. The initial rates were determined by xylenol orange assay in CHAPS buffer pH 9.5 for *Asp*EUGO and bis-Tris-propane buffer pH 9.5 for *Gb*EUGO. A final concentration of 50 mM buffer and 50 nM enzyme were used. The curves were fitted according to Hill. All reactions were performed as triplicate and the standard deviation is shown as error bars. Kinetic parameters are summarized in Table S6.

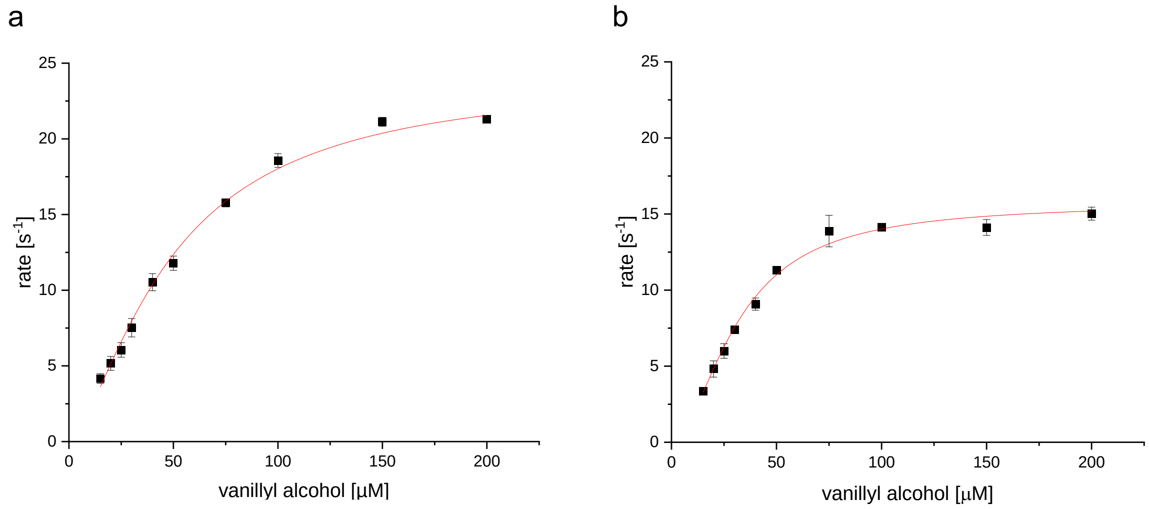

Figure S25. Michaelis-Menten kinetics of *Asp*EUGO (**a**) and *Gb*EUGO (**b**) on vanillyl alcohol (**5**) as substrate. The initial rates were determined by xylenol orange assay in CHAPS buffer pH 9.5 for *Asp*EUGO and bis-Tris-propane buffer pH 9.5 for *Gb*EUGO. A final concentration of 50 mM buffer and 50 nM enzyme were used. The curves were fitted according to Hill. All reactions were performed as triplicate and the standard deviation is shown as error bars. Kinetic parameters are summarized in Table S6.

Table S6. Kinetic parameters of *Asp*EUGO and *Gb*EUGO.

**Substrate**	**No**	**Enzyme**	**Model**	**K_M [µM]**	**k_{cat} [s^{-1}]**	**n**
Eugenol	**2**	*Asp*EUGO	Hill	28 ± 1	21 ± 0.4	2.8 ± 0.3
Eugenol	**2**	*Gb*EUGO	Hill	23 ± 1	23 ± 0.2	3.7 ± 0.3
Vanillyl alcohol	**5**	*Asp*EUGO	Hill	49 ± 3	24 ± 0.8	1.5 ± 0.1
Vanillyl alcohol	**5**	*Gb*EUGO	Hill	31 ± 1	16 ± 0.4	1.8 ± 0.1
4-Hydroxybenzyl alcohol	**4**	*Asp*EUGO	Hill	33 ± 2	19 ± 0.5	1.8 ± 0.1
4-Hydroxybenzyl alcohol	**4**	*Gb*EUGO	Michaelis-Menten	455 ± 50	17 ± 1	-
3,4-Dihydroxybenzyl alcohol	**7**	*Asp*EUGO	Hill	459 ± 33	2.4 ± 0.04	2.7 ± 0.8
3,4-Dihydroxybenzyl alcohol	**7**	*Gb*EUGO	Michaelis-Menten	5122 ± 827	4.9 ± 0.5	-

Supplementary Methods 7: Correlation of first shell residues of the catalytic center with substrate selectivity

Calculation of sequence-activity correlations

The natural logarithm of the activity data of each wildtype enzyme (Table S4) and the hydrophobicity and volume of single amino acids residues (Supplementary Data 2) were correlated in an automatized fashion by a custom R script (Supplementary Data 3). The respective residue characteristic for each enzyme was plotted against the logarithmic activity and a test for correlation was performed. The correlation coefficient r was calculated by the Pearson method (6) for every residue property-activity combination, where m_x and m_y represent the mean of the respective variables.

$$r = \frac{\sum(x - m_x) * (y - m_y)}{\sqrt{\sum(x - m_x)^2 * \sum(y - m_y)^2}} \quad (6)$$

As output, the r values were saved in a matrix and all individual plots were saved for manual inspection (Supplementary Data 3). From the matrix, substrates with structural motives of interest were selected to compare the influence of residues of the catalytic center with observed substrate acceptance (Figures 4 and S26 to S28).

Correlation of residue type in *ortho* position

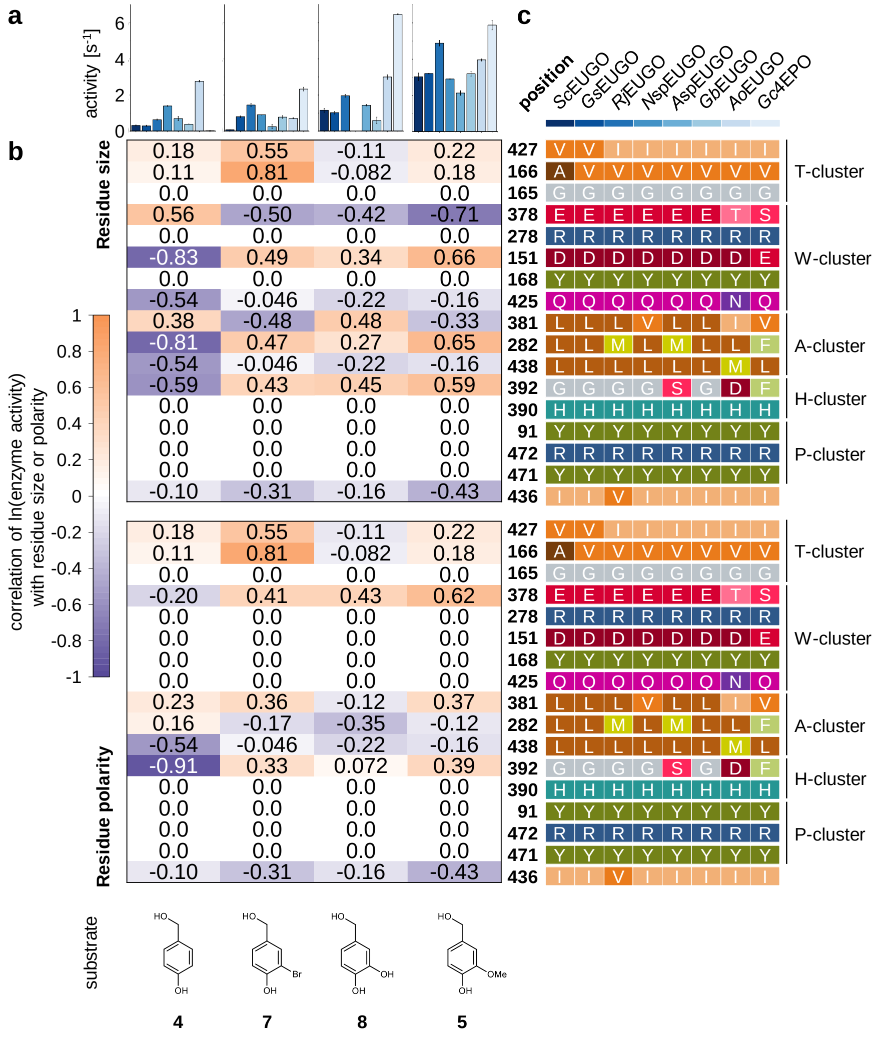

Figure S26. Analysis of the changes in the logarithm of the observed enzyme activity for benzyl alcohol derivatives with changing *ortho* substituent. **a** Observed activity for the enzymes of this study on the respective substrate (for details see Table S4). The error bars represent the standard deviation from a triplicate measurement. **b** Heat map for changes with the steric size (top) or and the polarity (bottom) of the first shell residues of the catalytic pocket. Primary data is provided in Supplementary Data 2 and 03. **c** Diversity of amino acids of the selected enzymes at respective positions.

Correlation of residue type in *para* position

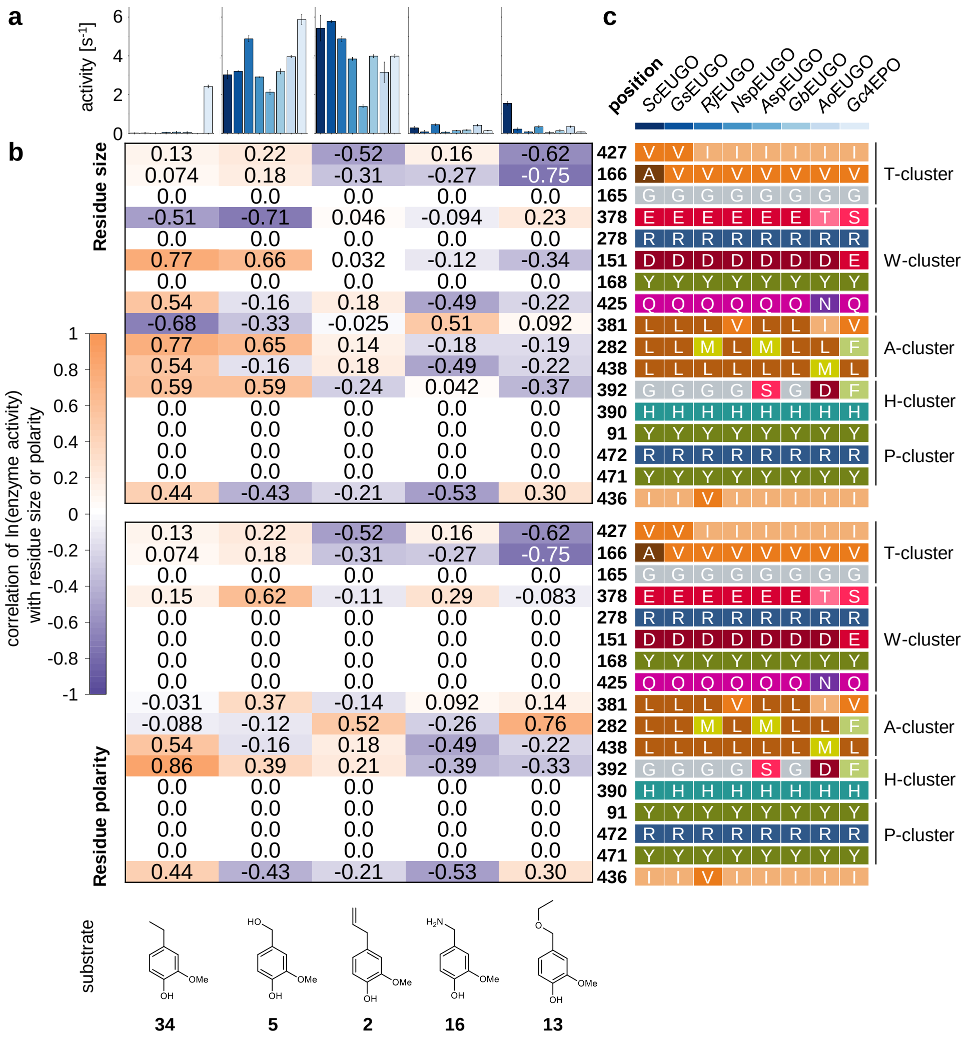

Figure S27. Analysis of the in the logarithm of the observed enzyme activity with changing *para* substituent of *ortho* methoxy phenols. **a** Observed activity for the enzymes of this study on the respective substrate (for details see Table S4). The error bars represent the standard deviation from a triplicate measurement. **b** Heat map for changes with the steric size (top) or and the polarity (bottom) of the first shell residues of the catalytic pocket. Primary data is provided in Supplementary Data 2 and 3. **c** Diversity of amino acids of the selected enzymes at respective positions.

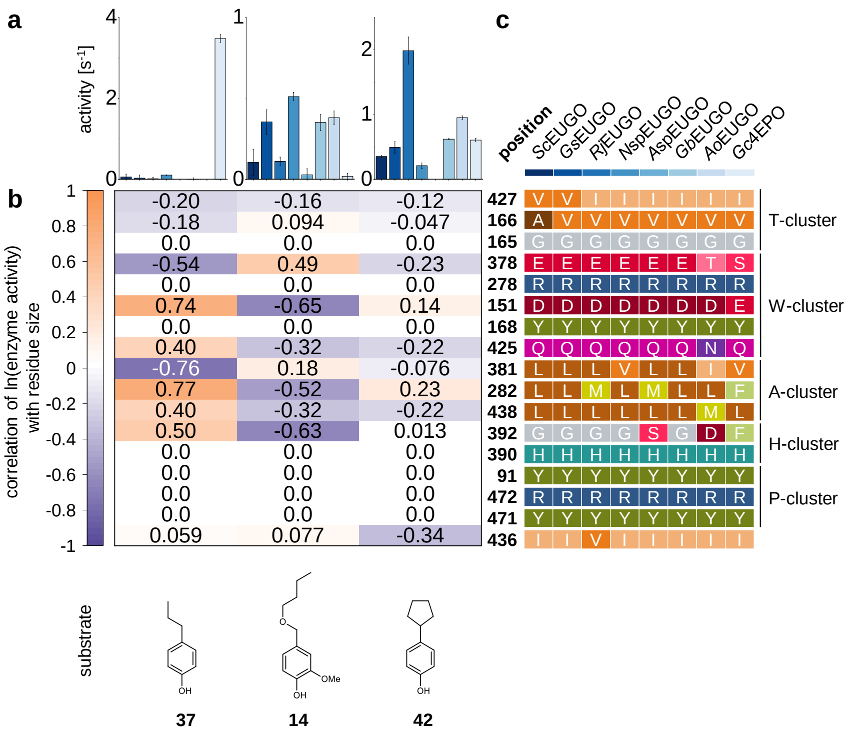

Figure S28. Analysis of the in the logarithm of the observed enzyme activity with changing size in *para* substituents. **a** Observed activity for the enzymes of this study on the respective substrate (for details see Table S4). The error bars represent the standard deviation from a triplicate measurement. **b** Heat map for changes with the steric size of the first shell residues of the catalytic pocket. Primary data is provided in Supplementary Data 2 and 3. **c** Diversity of amino acids of the selected enzymes at respective positions.

Supplementary Note 2: Oxidase screening

Background

Common oxidase screenings are coupled to secondary peroxidase reactions, like HRP, which react with the phenolic substrates of the 4-phenol oxidase.[14,15] Thus, a new screening approach was established within this study. Based on an *in vitro* hydrogen peroxide detection assay (xylenol orange assay), a high throughput screening assay was developed which is applicable in 96-well format and works in cell-free crude extract. In order to enable hydrogen peroxide detection, naturally present *E. coli* catalases like hydroperoxidase I and II had to be inactivated as these heme-dependent enzymes are several orders of magnitude faster than 4-phenol oxidases.[16] Inhibition of heme-containing catalases from human or other eukaryotes has previously been achieved by 3-amino-1,2,4-triazol (3AT).[17–19] Further, hydroxylamine (HA) was reported to efficiently inhibit the manganese-containing catalase of the Gram-negative bacterium *Thermoleophilum album*.[20] Both compounds were tested for their performance in various hydrogen peroxide concentrations (Figure S29). While the addition of 3AT to *E. coli* crude extract had no effect on the coloration of the screening solution, HA addition induced a similar response to hydrogen peroxide as in the buffer control. Therefore, in the 96-well screening assay with crude extracts, 1 mM HA was used to inhibit intrinsic catalase activity.

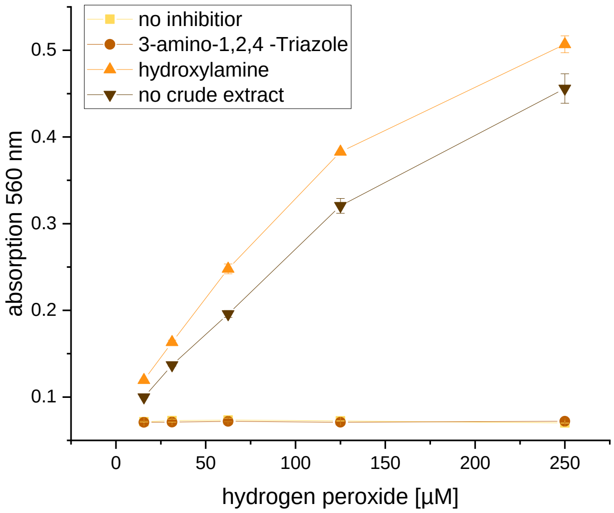

Figure S29. Colorization of the detection solution of the XO assay with increasing hydrogen peroxide concentration. *E. coli* BL21 (DE3) cell-free crude extract was able to complete remove the hydrogen peroxide from the solution without inhibitor. Therefore, 3-amino-1,2,4-triazole and hydroxylamine were tested as catalase inhibitors. With addition of 1 mM Hydroxylamine, a similar response is obtained as without crude extract while no effect is observed for 3-amino-1,2,4-triazole.

Overview

After creation and transformation of the site-saturation library, colonies were picked and transferred into a 96-well deep-well plate containing LB medium. The plate was incubated overnight as a preculture. From this preculture, a new 96-well deep-well plate was inoculated containing TB autoinduction medium. The main culture was grown for four hours at 37°C and at 25°C overnight. The cells were harvested by centrifugation and then chemically lysed. The protein containing supernatant was used for the assay after centrifugation. But for quantitative comparison of the kinetic rates between each well, the amount of protein had to be taken into consideration, as a higher enzyme yield correlates with higher hydrogen peroxide production. This would select for better production strains but not necessarily for improved enzyme variants. Thus, normalization of the enzyme amount was conducted. As normalization on the culture density excludes enzyme yield or disruption efficiency by the chemical lysis, the FAD

fluorescence of the crude extract was measured. Since 4-phenol oxidases contain a covalently bound FAD, the fluorescence of the crude extract gives a good estimation of the oxidase concentration. The measurement also includes the fluorescence of free FAD and other cell components but was found to be a better approximation than cell density. Immediately after fluorescence measurement, a substrate mix was added to each well and the hydrogen peroxide production was measured over time by the xylenol orange assay. The first sample was taken after 10 minutes. The duration for the second and third sample was adjusted for each individual substrate mixture based on the coloration of the detection solution. For substrates with wildtype activities <0.2 s^{-1}, samples were generally taken in the time frame of 45 minutes.

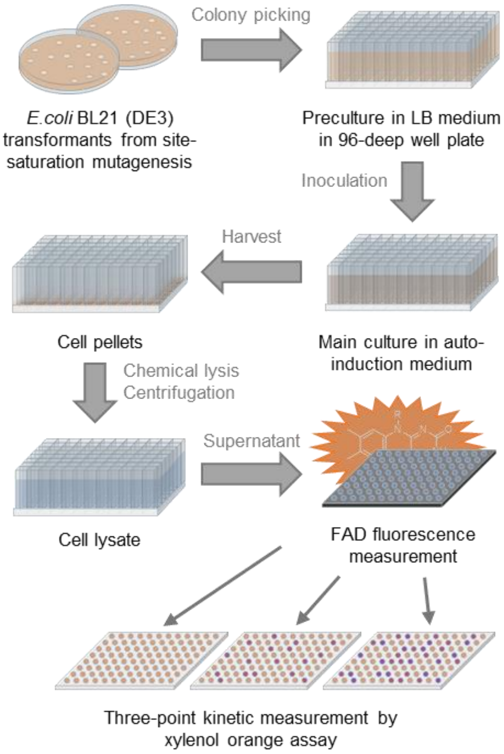

Figure S30. Schematic representation of the high throughput screening assay. Colonies after site-saturation mutagenesis were picked individually and incubated in 96-deep well plates in LB medium overnight at 37°C. The main cultures were inoculated with 10 µL of the preculture. Overnight cultivation was performed in auto-induction medium at 37°C for the first 4 h and at 25°C for the following 14 h. Cells were harvested by centrifugation and resuspended in 300 µL lysis solution. 50 µL of supernatant were transferred to a black 96-well plate and mixed with 47 µL 100 mM glycine NaOH buffer pH 9.5. The FAD fluorescence was measured (excitation: 441 nm, emission: 520 nm) to normalize for oxidase production level. Afterwards, a final concentration of 1 mM hydroxylamine was added to inhibit *E. coli* catalases and the reaction was started with the addition of 2 µL of a 100 mM substrate mix. At respective time points, 20 µL of the solution were taken and mixed in a fresh 96-well plate with 180 µL detection solution. After 30 min, the absorption was measured at 560 nm. The slope from three time points was used for evaluation.

Evaluation

According to the xylenol orange assay, the absorption was measured at 560 nm (A_{560}) after incubation for 30 minutes in the dark. For the evaluation of the screening results, the absorption values of each individual well were divided by the fluorescence counts (F_{FAD}) of the crude extract. Following the slope (m) of the absorption, normalized by the fluorescence (A_n) for all 3 time points (t) was determined.

$$A_n = \frac{A_{560}}{F_{FAD}} \tag{7}$$

$$m = \frac{\sum(t-\bar{t})(A_n - \overline{A_n})}{\sum(t-\bar{t})^2} \tag{8}$$

The slope of each individual well (m_i) was divided by the average slope of the wildtype (m_{WT}) controls to get a relative slope (m_r).

$$m_r = \frac{m_i}{m_{WT}} \tag{9}$$

Special attention was taken when calculating the slope of the individual wells for saturation of the detection solution. In that case the curve is not linear, and the last sample point had to be excluded from the slope calculation. An excel template is provided in the supplement for automatic evaluation.

Supplementary Methods 8: Creation and characterization of enzyme variants

Site-directed mutagenesis

QuikChange PCR with fully overlapping primers was performed (Table S7) using 100 ng template DNA mixed with a final amount of 0.4 µM of each primer and the PrimeSTAR Max DNA-polymerase (Takara) in a total volume of 25 µL. Successful amplification was verified by agarose gel electrophoresis, before the PCR product was digested by DpnI (10 U) and afterwards, was transformed in *E. coli* DH5α. Mutations were validated by sequencing, before transformation in the expression host *E. coli* BL21 (DE3) was conducted.

Generation of site-saturation libraries

The mutant libraries were created by the QuikChange procedure described above using primer with the degenerate codon NNK (Table S7). After QuikChange and DpnI digestion, the plasmids were transformed into *E. coli* BL21 (DE3) by heat shock transformation and plated on LB-agar plates containing 100 mg L^{-1} ampicillin. The colonies formed were used for further cultivation and usage in the screening.

Oxidase screening assay

Colonies were picked after transformation and transferred into individual wells of a 96-well deep-well plate (square well, round bottom) containing 1 mL of LB-medium (10 g L^{-1} tryptone, 5 g L^{-1} yeast extract, 10 g L^{-1} sodium chloride) with 150 mg L^{-1} ampicillin. For every library, a full 96-well plate was used to achieve a library coverage >90%. Every plate contained wildtype controls which were grown in a separate preculture and placed diagonally from well A1 to H8. The plates were closed by foil and grown overnight at 37°C and 130 rpm. Then, a new 96-well deep-well plate (square well, round bottom) containing 1 mL of TB auto-induction medium (12 g L^{-1} peptone, 24 g L^{-1} yeast extract. 100 mM potassium phosphate buffer pH 7.0, 0.5 g L^{-1} glucose, 2.0 g L^{-1} lactose, 5.0 mL L^{-1} glycerol) and 150 mg L^{-1} ampicillin was inoculated with 10 µL of the preculture and also closed with foil. The plates were incubated for 4 hours at 37°C and following at 25°C overnight. The plates of the preculture were stored overnight at 8°C to allow for inoculation of another preculture for sequencing after screening.

The cells were harvested by centrifugation (20 min. at 4,347 x g and 4°C) and the supernatant was carefully discarded. The cell pellets were lysed chemically by resuspension in 300 µL B-PER™ Bacterial Protein Extraction Reagent (ThermoFisher Scientific) containing 0.25 mg mL^{-1} lysozyme and 0.05 mg mL^{-1} DNase. After 15 min incubation at room temperature, the crude extract was separated from the cell debris by centrifugation (30 min. at 4,347 x g and 4°C). From each well, 50 µL of the crude extract were transferred into a black 96-well plate containing 47 µL of 100 mM Glycine-NaOH buffer pH 9.5 and the FAD fluorescence was measured with an excitation wavelength of 441 nm and an emission wavelength of 520 nm, for normalization. Then, 1 µL of a 100 mM hydroxylamine hydrochloride solution was added to inhibit *E. coli* catalases and the plate was incubated for 5 min at 25°C and 750 rpm.

The reaction was started by the addition of 2 µL substrate solution containing 100 mM of up to three substrates to reach a final substrate concentration of 2 mM, respectively. The plate was incubated at 25°C and 750 rpm and at three time points ranging between 10 and 90 minutes, samples were taken to determine the hydrogen peroxide concentration by xylenol orange assay. For evaluation of the results, please refer to Supplementary Note 2. Wells with highest activity were selected and a 10 mL LB culture was inoculated from the respective preculture for plasmids preparation. Finally, mutations were validated by sequencing.

Crude extract assay

A 50 mL main culture was prepared for a respective enzyme variant and the wildtype as described above. The cells were harvested in 10 mL aliquots by centrifugation (20 min at 4,347 x g) and the cell wet weight (CWW) was determined. Chemical lysis was performed by addition of 6 mL B-PER™ Bacterial Protein Extraction Reagent (ThermoFisher Scientific) and water mixture (2:1 Bper:water) per gram of CWW. After 15 minutes incubation at RT, the cell debris was separated by centrifugation (10 min at 15,000 x g and 15°C). 5 to 10 µL of crude extract were transferred into each well of a black 96-well plate which was filled up with Glycine-NaOH buffer pH 9.5 to a final volume of 97 µL. Similar to the screening assay, hydroxyl amine was added, and the FAD fluorescence was determined, before the assay was started by addition of a final substrate concentration of 1 mM. Samples for the xylenol orange assay were taken after 4 and 8 minutes. The evaluation was performed according to the screening assay.

List of mutagenesis primers

Table S7. Primer list.

Enzyme	Primer	Sequence
*Sc*EUGO	V427X_for	5´- GAAGGACTATGCAGCGCAGTTTNNKGTCGGGTTACGGGAAATG -3´
*Sc*EUGO	V427X_rev	5´- CATTTCCCGTAACCCGACMNNAAACTGCGCTGCATAGTCCTTC -3´
*Sc*EUGO	A166X_for	5´- CGTTAGATCGTGGGNNKGGCTATACCCCGTATG -3´
*Sc*EUGO	A166X_rev	5´- CATACGGGGTATAGCCMNNCCCACGATCTAACG -3´
*Sc*EUGO	L381X_for	5´- ATGAAANNKATGGACTGGATTCCCGGTGCAGGACATGTCGGTTTTAGCCC -3´
*Sc*EUGO	L381X_rev	5´- GTCCATMNNTTTCATCTCAGACAGGGAGGGGATACCATTGTTGATTTTGT -3´
*Sc*EUGO	Q425X_for	5´- GGACTATGCAGCGNNKTTTGTGGTCGGGTTACG -3´
*Sc*EUGO	Q425X_rev	5´- CGTAACCCGACCACAAAMNNCGCTGCATAGTCC -3´
*Sc*EUGO	L282X_for	5´- ATCATCNNKGATGCCGCCGTAGTGTCTCAACGGGCCGATTGGTACGATGG -3´
*Sc*EUGO	L282X_rev	5´- GCATCMNNGATGATGTTGCGCAGAACCGGTACATTCTGCAGCGGAGCCAT -3´
*Sc*EUGO	E378T_for	5´- TGTCTACGATGAAACTTATGGACTGGATTCCCGGTGCAGGACATGTCGGT -3´
*Sc*EUGO	E378T_rev	5´- TTCATCGTAGACAGGGAGGGGATACCATTGTTGATTTTGTGGCGATCTTG -3´
*Sc*EUGO	E378Q_for	5´- TGTCTCAGATGAAACTTATGGACTGGATTCCCGGTGCAGGACATGTCGGT -3´
*Sc*EUGO	E378Q_for	5´- TTCATCTGAGACAGGGAGGGGATACCATTGTTGATTTTGTGGCGATCTTG -3´
*Gc*4EPO	I432X_for	5´- GCAATTCNNKATTGGCCTGCGTGAGATGCACCACATTTGTTTGCCGCTGT -3´
*Gc*4EPO	I432X_rev	5´- CCAATMNNGAATTGCGCAAGATAGTCGATGCCGTGGTCGTCGCTGATTCG -3´
*Gc*4EPO	V166X_for	5´- CATGGTNNKGGCTACACCCCCTATGCCGACCACTCGATGTGGCGGTGCGG -3´
*Gc*4EPO	V166X_rev	5´- GTAGCCMNNACCATGCTCCATGGTGTTGCCCATGACGCTGCCCCAGTCGA -3´

List of created variants

Table S8. Overview of enzyme variants generated in this study.

Enzyme	Variant	Cluster	Method	Target	Purified	Expression host
*Sc*EUGO	A166G	T	Site-saturation mutagenesis	(i)	No	*E. coli* BL21 (DE3)
*Sc*EUGO	A166S	T	Site-saturation mutagenesis	(i)	No	*E. coli* BL21 (DE3)
*Sc*EUGO	V427A	T	Site-saturation mutagenesis	(i)	No	*E. coli* BL21 (DE3)
*Sc*EUGO	V427T	T	Site-saturation mutagenesis	(i)	No	*E. coli* BL21 (DE3)
*Sc*EUGO	V427Y	T	Site-saturation mutagenesis	(i)	Yes	*E. coli* BL21 (DE3)
*Gc*4EPO	V166D	T	Site-saturation mutagenesis	(i)	Yes	*E. coli* BL21 (DE3)
*Sc*EUGO	E378T	W	Quick Change	(ii)	Yes	*E. coli* BL21 (DE3)
*Sc*EUGO	E378Q	W	Quick Change	(ii)	Yes	*E. coli* BL21 (DE3)
*Sc*EUGO	Q425E	W	Site-saturation mutagenesis	(ii)	Yes	*E. coli* BL21 (DE3)
*Sc*EUGO	D151N	W	Quick Change	(ii)	Yes	*E. coli* BL21 (DE3)
*Sc*EUGO	D151N Q425E	W	Quick Change	(ii)	Yes	*E. coli* BL21 (DE3)
*Sc*EUGO	L381I	A	Site-saturation mutagenesis	(iii)	No	*E. coli* BL21 (DE3)
*Sc*EUGO	V427F	T	Site-saturation mutagenesis	(iii)	Yes	*E. coli* BL21 (DE3)
*Sc*EUGO	V427I	T	Site-saturation mutagenesis	(iii)	Yes	*E. coli* BL21 (DE3)
*Sc*EUGO	V427M	T	Site-saturation mutagenesis	(iii)	No	*E. coli* BL21 (DE3)
*Sc*EUGO	V427I L282M	T, A	Site-saturation mutagenesis	(iii)	Yes	*E. coli* BL21 (DE3)

Activity of purified enzyme variants

Table S9. Activity of purified enzyme variants. The rate was determined by xylenol orange assay in 50 mM potassium phosphate buffer pH 7.5 wit 2 mM of the respective substrate at 25°C. Errors represent the standard deviation of a triplicate measurement. The cells are colored in a gradient from highest (blue) to lowest (light orange) rate. Product formation was validated by GC-MS. The measured substrates and detected products are listed in Table S5.

No	***Sc*EUGO V427Y**	***Gc*4EPO V166D**	***Sc*EUGO E378Q**	***Sc*EUGO E378T**	***Sc*EUGO Q425E**	***Sc*EUGO D151N**	***Sc*EUGO D151N Q425E**	***Sc*EUGO V427F**	***Sc*EUGO V427I**	***Sc*EUGO V427I L282M**
**1**	0.12 ± 0.06	0.11 ± 0.09	n.m.	n.m.	n.m.	n.m.	n.m.	0.71 ± 0.04	1.33 ± 0.09	n.m.
**2**	2.07 ± 0.01	0.36 ± 0.02	0.11 ± 0.05	0.23 ± 0.02	0.55 ± 0.08	0.06 ± 0.05	0.05 ± 0.01	5.36 ± 0.07	7.20 ± 0.30	4.33 ± 0.11
**3**	n.d.	n.m.	0.16 ± 0.03	0.46 ± 0.01	0.34 ± 0.08	0.08 ± 0.01	n.d.	0.44 ± 0.07	1.11 ± 0.06	1.04 ± 0.07
**4**	0.09 ± 0.03	2.44 ± 0.08	n.m.	n.m.	n.m.	n.m.	n.m.	0.53 ± 0.07	1.51 ± 0.13	n.m.
**5**	2.50 ± 0.17	5.01 ± 0.22	3.14 ± 0.22	1.68 ± 0.17	3.12 ± 0.05	0.52 ± 0.09	n.d.	4.86 ± 0.43	2.99 ± 0.13	1.55 ± 0.06
**6**	0.14 ± 0.02	n.m.	n.m.	2.84 ± 0.12	4.05 ± 0.64	0.14 ± 0.04	0.22 ± 0.03	0.40 ± 0.06	1.91 ± 0.11	2.10 ± 0.09
**7**	n.m.	2.87 ± 0.17	n.m.	n.m.	0.41 ± 0.11	n.m.	n.m.	n.m.	3.19 ± 0.13	n.m.
**8**	0.36 ± 0.02	14.70 ± 0.47	n.m.	n.m.	0.25 ± 0.02	n.m.	n.m.	0.62 ± 0.08	0.52 ± 0.08	0.42 ± 0.02
**9**	n.m.	n.m.	n.m.	n.m.	0.10 ± 0.05	n.m.	n.m.	n.m.	3.29 ± 0.05	2.76 ± 0.30
**10**	0.07 ± 0.04	n.m.	n.m.	n.m.	n.m.	n.m.	n.m.	n.m.	n.m.	n.m.
**11**	n.m.	0.23 ± 0.04	n.m.	n.m.	n.m.	n.m.	n.m.	n.m.	0.43 ± 0.03	n.m.
**12**	n.m.	n.m.	0.40 ± 0.04	1.08 ± 0.03	0.40 ± 0.03	n.m.	0.07 ± 0.03	n.m.	1.58 ± 0.13	0.84 ± 0.08
**13**	n.m.	n.m.	n.m.	n.m.	n.d.	n.m.	n.m.	n.m.	1.25 ± 0.02	0.29 ± 0.03
**15**	n.m.	n.m.	n.m.	n.m.	n.d.	n.m.	n.m.	n.m.	0.09 ± 0.05	n.m.
**16**	n.m.	n.d.	0.12 ± 0.03	0.12 ± 0.08	0.98 ± 0.04	n.d.	n.m.	n.m.	0.98 ± 0.04	0.21 ± 0.03
**17**	n.m.	0.06 ± 0.04	n.m.	n.m.	n.m.	n.m.	n.m.	n.m.	n.m.	n.m.
**18**	n.m.	n.m.	n.m.	n.m.	n.m.	n.m.	n.m.	n.m.	n.d.	n.m.
**24**	n.m.	n.d.	n.m.	n.m.	n.m.	n.m.	n.m.	n.m.	n.m.	n.m.
**25**	n.m.	0.27 ± 0.22	n.m.	n.m.	n.m.	n.m.	n.m.	n.m.	n.m.	n.m.
**26**	n.m.	n.d.	n.m.	n.d.	n.m.	n.m.	n.m.	n.m.	n.d.	n.m.
**27**	n.m.	0.16 ± 0.05	n.m.	n.m.	n.m.	n.m.	n.m.	n.m.	n.m.	n.m.
**28**	n.m.	n.d.	n.m.	n.m.	n.m.	n.m.	n.m.	n.m.	n.m.	n.m.
**29**	n.m.	n.d.	n.m.	n.m.	n.m.	n.m.	n.m.	n.m.	n.m.	n.m.
**32**	n.d.	0.96 ± 0.07	n.m.	n.d.	n.m.	n.d.	n.d.	n.d.	n.m.	n.m.
**33**	n.m.	n.m.	n.m.	n.m.	n.m.	n.m.	n.m.	n.m.	0.30 ± 0.24	n.m.
**34**	n.m.	n.m.	0.20 ± 0.04	0.22 ± 0.01	n.m.	0.04 ± 0.03	n.d.	n.m.	n.d.	n.d.
**36**	n.d.	n.d.	n.m.	n.m.	n.m.	n.m.	n.m.	n.m.	n.m.	n.m.
**37**	n.m.	0.61 ± 0.01	n.m.	n.m.	n.m.	0.05 ± 0.04	n.d.	n.m.	n.m.	n.m.
**38**	n.m.	n.d.	n.m.	n.m.	n.m.	n.m.	n.m.	n.m.	n.d.	n.m.
**39**	n.m.	n.m.	n.m.	n.m.	n.m.	0.08 ± 0.04	n.m.	n.m.	0.09 ± 0.03	0.18 ± 0.07
**41**	n.m.	0.63 ± 0.05	n.m.	n.m.	0.04 ± 0.01	n.m.	n.m.	n.m.	n.m.	n.d.
**42**	n.m.	n.m.	n.m.	n.m.	n.m.	0.08 ± 0.02	0.08 ± 0.01	n.m.	3.66 ± 0.07	4.87 ± 0.23
**44**	n.m.	n.m.	n.m.	n.m.	n.m.	n.m.	n.m.	n.m.	0.13 ± 0.03	0.22 ± 0.01

n.d. = not detected, n.m. = not measured

Crude extract assay for *Sc*EUGO A166X and V427X libraries for target (i)

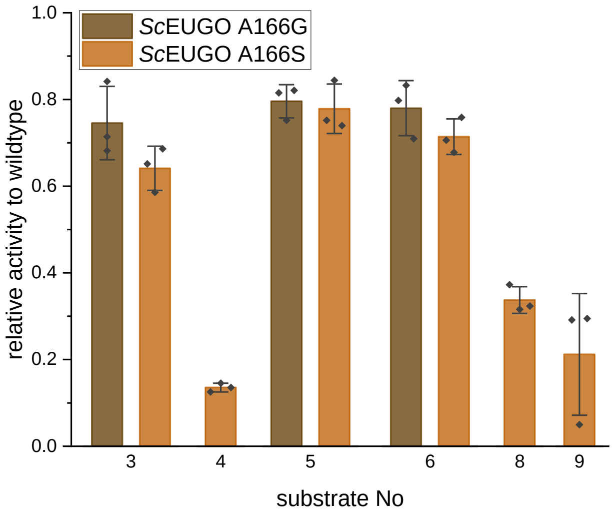

Figure S31. Relative activity of *Sc*EUGO A166G and A166S compared to the wildtype in a crude extract screening on selected substrates. Substrate No **4**, **8** and **9** were only tested for *Sc*EUGO A166S as this variant appeared to be the more interesting one. Error bars represent the standard deviation of a triplicate measurement.

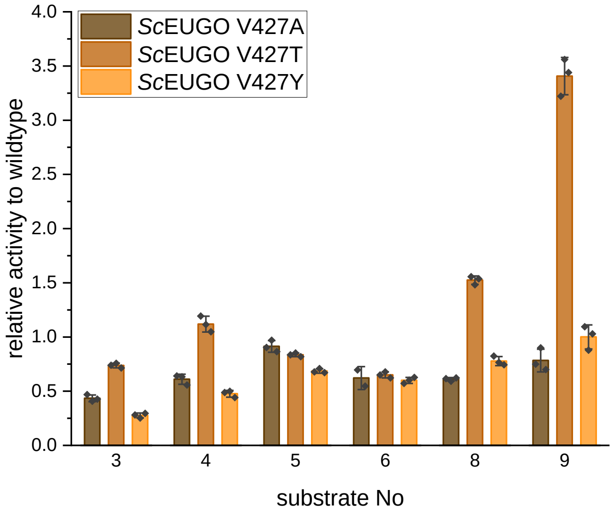

Figure S32. Relative activity of *Sc*EUGO V427A, V427T and V427Y compared to the wildtype in a crude extract screening on selected substrates. Error bars represent the standard deviation of a triplicate measurement.

Michaelis-Menten kinetics of *Gc*4EPO wildtype and V166D variant

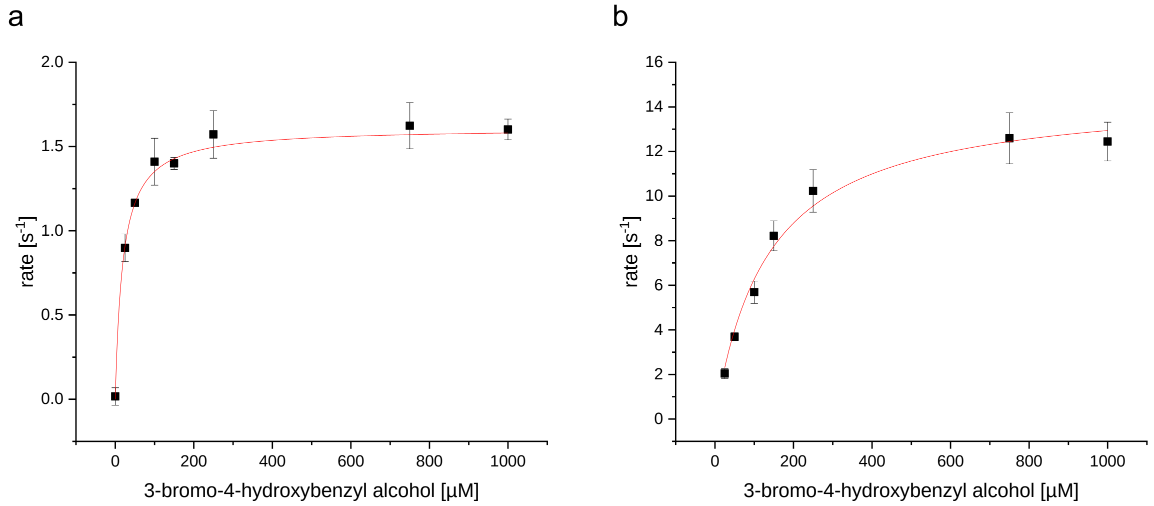

Figure S33. Michaelis-Menten kinetic of *Gc*4EPO wildtype (**a**) and *Gc*4EPO V166D (**b**) on 3-bromo-4-hydroxybenzyl alcohol (**8**) as substrate in 50 mM potassium phosphate buffer pH 7.5. Final enzyme concentrations of 50 nM for the wildtype and 17.5 nM for the variant were used. The curves follow the classical Michaelis-Menten behavior. All reactions were performed as triplicate and the standard deviation is shown as error bars. Kinetic parameters are summarized in Table S10.

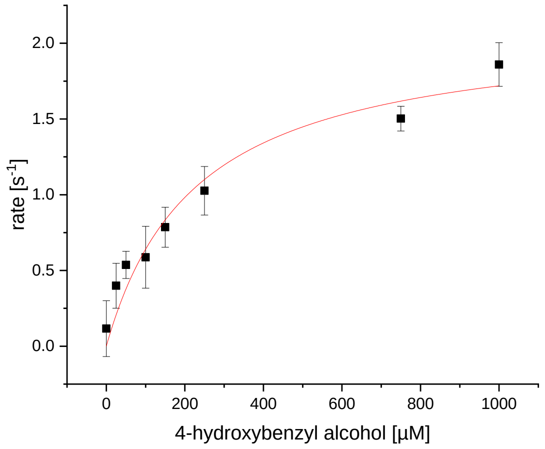

Figure S34. Michaelis-Menten kinetic of *Gc*4EPO V166D on 4-hydroxybenzyl alcohol (**4**) as substrate in 50 mM potassium phosphate buffer pH 7.5. A final enzyme concentration of 17.5 nM was used. The wildtype was no active enough on the substrate to record a kinetic. The curve follows the classical Michaelis-Menten behavior. All reactions were performed as triplicate and the standard deviation is shown as error bars. Kinetic parameters are summarized in Table S10.

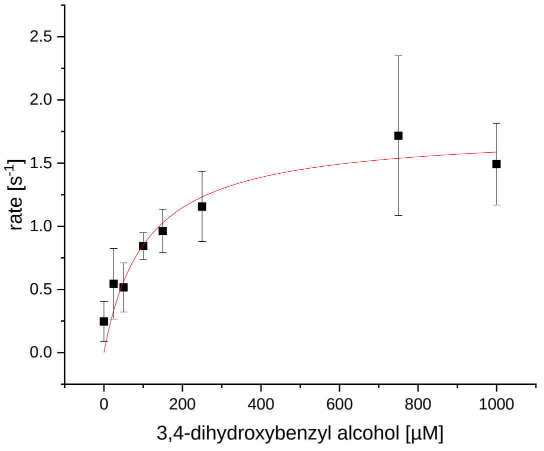

Figure S35. Michaelis-Menten kinetic of *Gc*4EPO V166D on 3,4-dihydroxybenzyl alcohol (**7**) as substrate in 50 mM potassium phosphate buffer pH 7.5. A final enzyme concentration of 17.5 nM was used. The wildtype was no active enough on the substrate to record a kinetic. The curve follows the classical Michaelis-Menten behavior. All reactions were performed as triplicate and the standard deviation is shown as error bars. Kinetic parameters are summarized in Table S10.

Table S10. Kinetic parameters for *Gc*4EPO wildtype and V166D variant.

Substrate	No	Enzyme	Model	K_M	k_{cat}
3-Bromo-4-hydroxybenzyl alcohol	**8**	*Gc*4EPO	Michaelis-Menten	19 ± 1	1.6 ± 0.02
3-Bromo-4-hydroxybenzyl alcohol	**8**	*Gc*4EPO V166D	Michaelis-Menten	134 ± 17	14.7 ± 0.6
4-Hydroxybenzyl alcohol	**4**	*Gc*4EPO V166D	Michaelis-Menten	230 ± 63	2.1 ± 0.2
3,4-Dihydroxybenzyl alcohol	**7**	*Gc*4EPO V166D	Michaelis-Menten	106 ± 33	1.8 ± 0.2

Structural analysis of Gc4EPO V166D

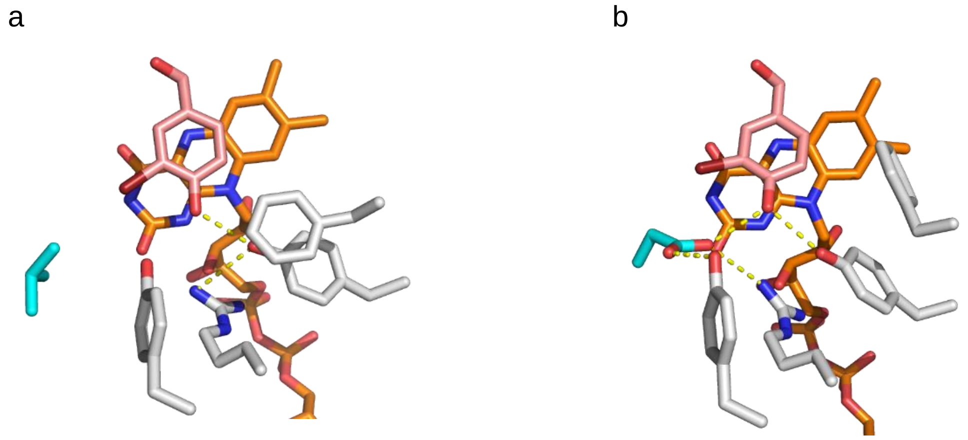

Figure S36. Catalytic center of *Gc*4EPO WT (**a**) and V166D (**b**) in complex with 3-bromo-4-hydroxybenzyl alcohol (**8**) after refinement by 0.1 ns molecular dynamics simulation. The FAD cofactor is shown in orange and the substrate in light red. Polar interaction between the substrate and the amino acid residues are depicted as dashed, yellow lines. The residues of the P-cluster, Y92, Y476 and R477, and Phe397 are depicted as sticks. The changed residue in position 166 is shown in teal. Glu166 interacts with the residues of the P-cluster and causes the substrate to turn in the direction of the substrate entrance tunnel.

Michaelis-Menten kinetic and structural analysis of *Sc*EUGO Q425E

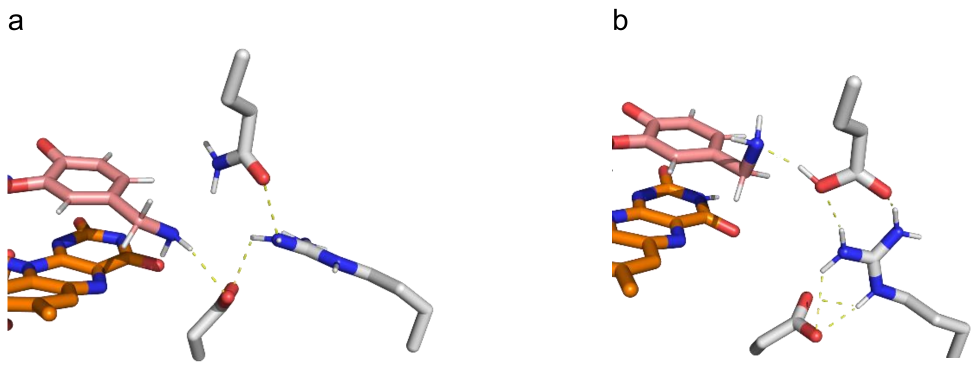

Figure S37. Catalytic center of *Sc*EUGO WT (**a**) and Q425E (**b**) docked with vanillyl amine (**16**) after refinement by 0.1 ns molecular dynamics simulation. The FAD cofactor is shown in orange and the substrate in light red. Polar interaction between the substrate and the amino acid residues are depicted as dashed, yellow lines. The residues of the W-cluster, D151 and R366, and Q/E425 are depicted as sticks. In the wildtype, the amine group interacts with D151 which results in a non-optimal positioning of the benzylic hydrogens for a transfer to the FAD cofactor. The Q425E exchange results in a change in the hydrogen bond network. E425 now interacts with the amine group which positions the benzylic hydrogens in direction of the FAD.

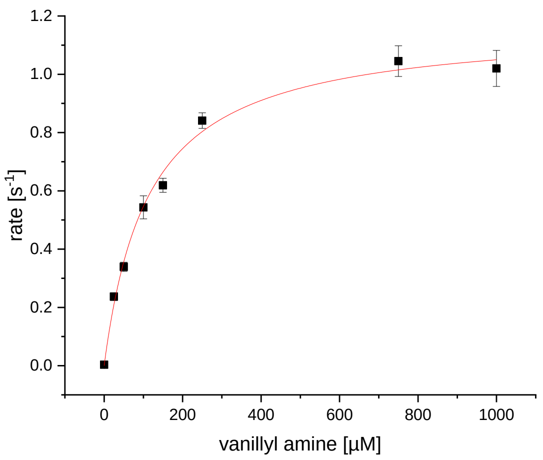

Figure S38. Michaelis-Menten kinetic of *Sc*EUGO Q425E on vanillyl amine (**16**) as substrate in 50 mM potassium phosphate buffer pH 7.5. A final enzyme concentration of 250 nM was applied. The curve follows the classical Michaelis-Menten behavior. All reactions were performed as triplicate and the standard deviation is shown as error bars. The K_M value was determined to 114 ± 13 µM while the k_{cat} value was 1.2 ± 0.06 s^{-1}.

Crude extract assay for *Sc*EUGO L381X and V427X libraries for target (iii)

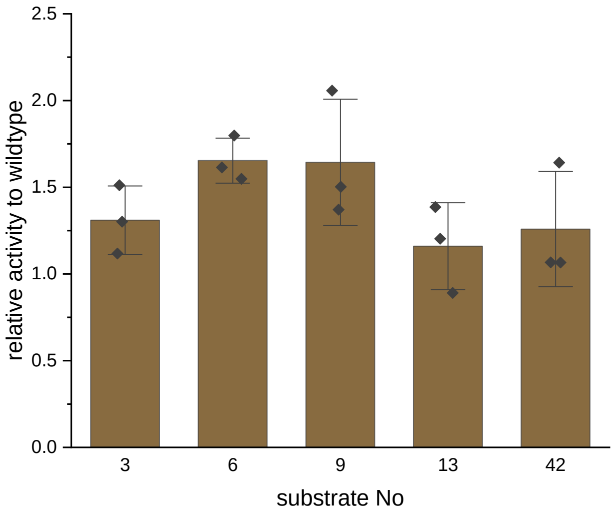

Figure S39. Relative activity of *Sc*EUGO L381I compared to the wildtype in a crude extract screening on selected substrates. Error bars represent the standard deviation of a triplicate measurement.

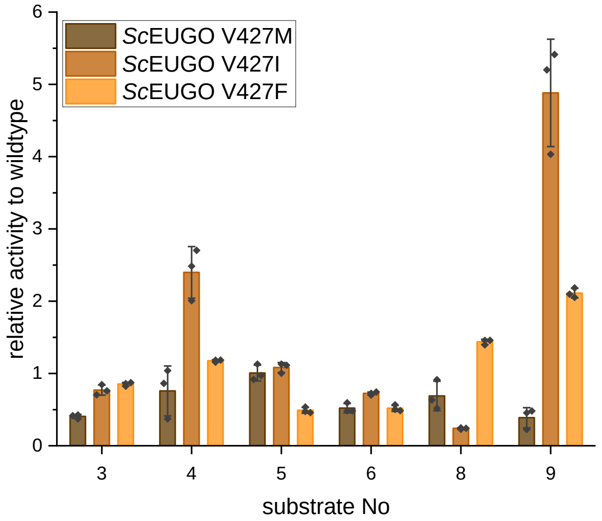

Figure S40. Relative activity of *Sc*EUGO V427M, V427I and V427F compared to the wildtype in a crude extract screening on selected substrates. Error bars represent the standard deviation of a triplicate measurement.

Michaelis-Menten kinetic and structural analysis of *Sc*EUGO V427I L282M

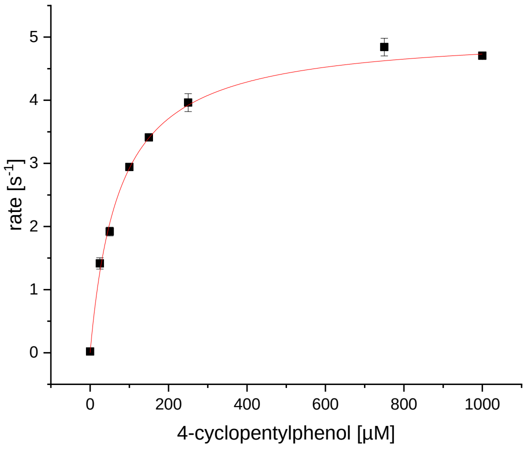

Figure S41. Michaelis-Menten kinetic of *Sc*EUGO V427I L282M on 4-cyclopentylphenol (**42**) as substrate in 50 mM potassium phosphate buffer pH 7.5. A final enzyme concentration of 40 nM was applied. The curve follows the classical Michaelis-Menten behavior. All reactions were performed as triplicate and the standard deviation is shown as error bars. The K_M value was determined to 74 ± 4 µM while the k_{cat} value was 5.1 ± 0.07 s^{-1}.

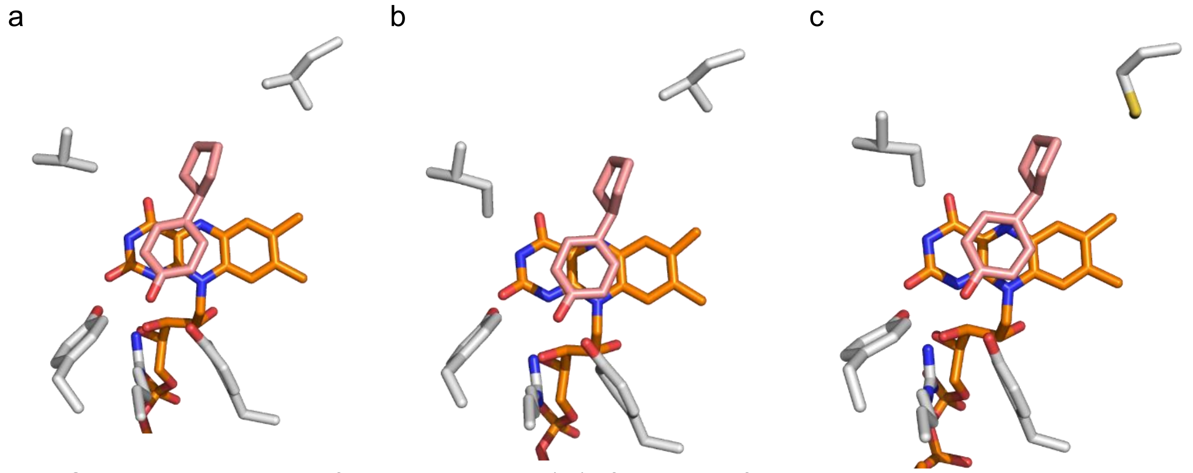

Figure S42. Autodocking results of 4-cyclopentylphenol (**42**) after 0.1 ns refinement by molecular dynamics simulations in *Sc*EUGO WT (**a**), *Sc*EUGO V427I (**b**) and *Sc*EUGO V427I L282M (**c**). The FAD cofactor is shown in orange **42** in light red. I427 pushes the substrate into the catalytic center while no immediate effect for M282 can be observed.

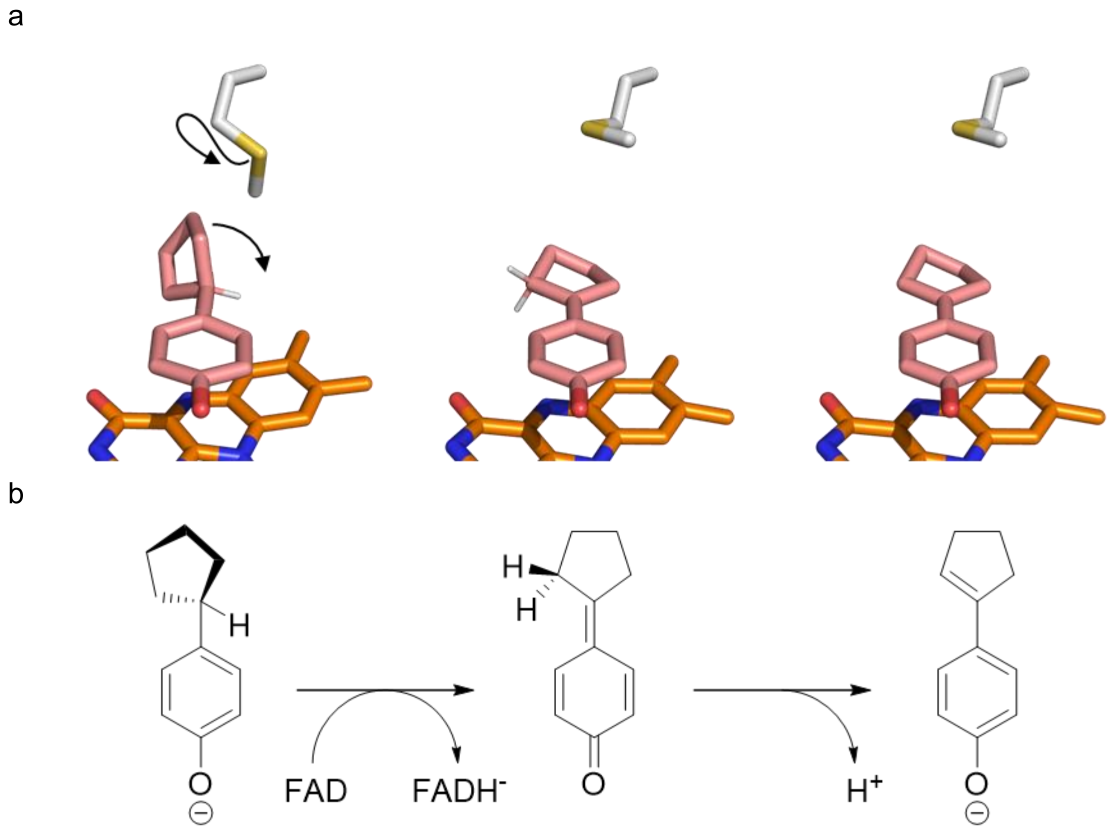

Figure 43. Proposed movement of M282 during conversion of 4-cyclopentylphenol (**42**). **a** Structural data obtained from autodocking experiments in *Sc*EUGO V427I L282M variant using **42** and the dehydrogenation product 4-(cyclopent-1-en-1-yl)phenol as ligands. The docked structures were refined by molecular dynamic simulation. **b** Schematic representation of the mechanistical steps. The hydride abstraction to the FAD causes the benzylic carbon to change from sp^3 to sp^2 hybridization. As a consequence, the cyclopentyl ring moves in plane with the phenolic ring. M282 has to move to generate space for the intermediate and residue fills the space on top the cyclopentyl ring.

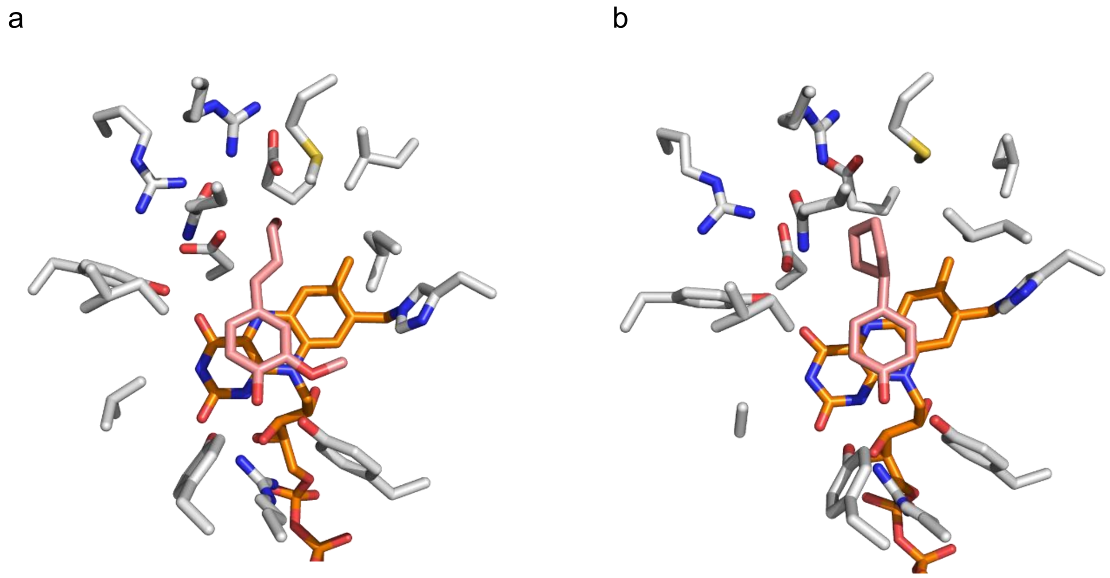

Figure S44. Comparison of the catalytic center from the crystal structure of *Rj*EUGO (PDB: 5FXE, **a**) and the homology model of *Sc*EUGO V427I L282M (**b**) docked with 4-cyclopentylphenol (**42**) after 0.1 ns refinement by molecular dynamics simulation. The FAD cofactor is shown in orange and the respective substrate in light red. All first shell residues of the catalytic center are shown as sticks.

Structural analysis of *Sc*EUGO V427I

a b

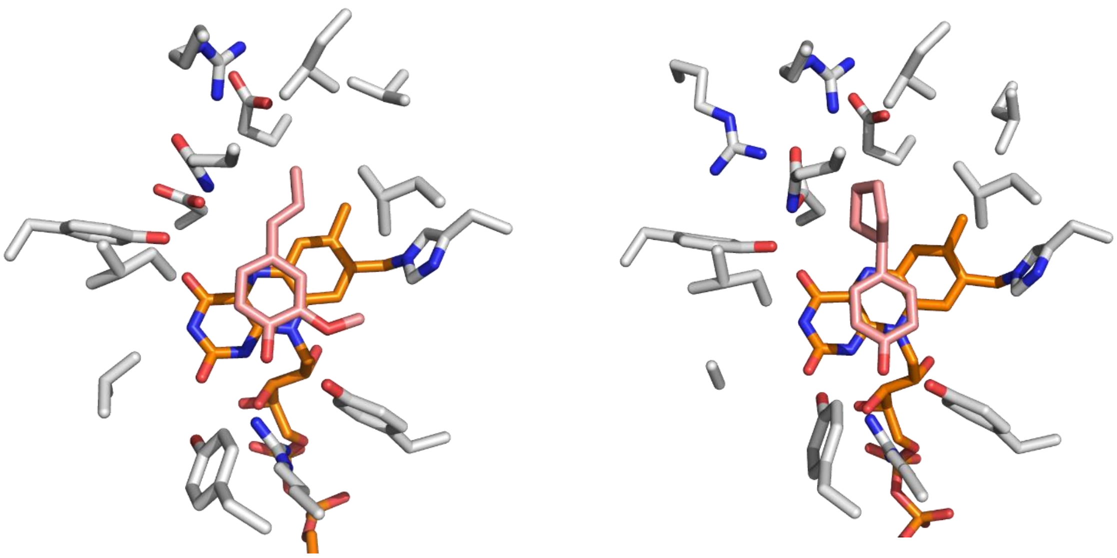

Figure S45. Comparison of the catalytic center from the homology model of *N*spEUGO (**a**) docked with eugenol (**2**) and *Sc*EUGO V427I (**b**) docked with 4-cyclopentylphenol (**42**) after 0.1 ns refinement by molecular dynamics simulation. The FAD cofactor is shown in orange and the respective substrate in light red. All first shell residues of the catalytic center are shown as sticks.

Supplementary References

1. Gibson, D. G. *et al.* Enzymatic assembly of DNA molecules up to several hundred kilobases. *Nature Methods* 6, 343–345; 10.1038/nmeth.1318 (2009).
2. Jin, J., Mazon, H., van den Heuvel, R. H. H., Janssen, D. B. & Fraaije, M. W. Discovery of a eugenol oxidase from *Rhodococcus* sp. strain RHA1. *The FEBS Journal* 274, 2311–2321; 10.1111/j.1742-4658.2007.05767.x (2007).
3. Eggerichs, D., Zilske, K. & Tischler, D. Large scale production of vanillin using an eugenol oxidase from *Nocardioides* sp. YR527. *Molecular Catalysis* 546, 113277; 10.1016/j.mcat.2023.113277 (2023).
4. Alvigini, L. *et al.* Discovery, Biocatalytic exploration and structural analysis of a 4-ethylphenol oxidase from *Gulosibacter chungangensis*. *Chembiochem: A European Journal of Chemical Biology* 22, 3225–3233; 10.1002/cbic.202100457 (2021).
5. Tamura, K., Stecher, G. & Kumar, S. MEGA11: Molecular Evolutionary Genetics Analysis version 11. *Molecular Biology and Evolution* 38, 3022-3027 (2021).
6. Ewing, T. A., van Noord, A., Paul, C. E. & van Berkel, W. J. H. A xylenol orange-based screening assay for the substrate specificity of flavin-dependent *para*-phenol oxidases. *Molecules (Basel, Switzerland)* 23; 10.3390/molecules23010164 (2018).
7. Gay, C., Collins, J. & Gebicki, J. M. Determination of iron in solutions with the ferric-xylenol orange complex. *Analytical Biochemistry* 273, 143–148; 10.1006/abio.1999.4207 (1999).
8. Krieger, E. & Vriend, G. YASARA View - molecular graphics for all devices - from smartphones to workstations. *Bioinformatics (Oxford, England)* 30, 2981–2982; 10.1093/bioinformatics/btu426 (2014).
9. Schrödinger, L. L. C. & DeLano, W. *PyMOL* (2020).
10. Trott, O. & Olson, A. J. AutoDock Vina: Improving the speed and accuracy of docking with a new scoring function, efficient optimization, and multithreading. *Journal of Computational Chemistry* 31, 455–461; 10.1002/jcc.21334 (2010).
11. Hill, A. V. A new mathematical treatment of changes of ionic concentration in muscle and nerve under the action of electric currents, with a theory as to their mode of excitation. *The Journal of Physiology* 40, 190–224; 10.1113/jphysiol.1910.sp001366 (1910).
12. Haldane, J. B. S. *Enzymes.* 3rd ed. (MIT Press, Cambridge, MA, 1930).
13. LiCata, V. J. & Allewell, N. M. Is substrate inhibition a consequence of allostery in aspartate transcarbamylase? *Biophysical Chemistry* 64, 225–234; 10.1016/s0301-4622(96)02204-1 (1997).
14. Debon, A. *et al.* Ultrahigh-throughput screening enables efficient single-round oxidase remodelling. *Nature Catalysis* 2, 740–747; 10.1038/s41929-019-0340-5 (2019).
15. Weiß, M. S., Pavlidis, I. V., Vickers, C., Höhne, M. & Bornscheuer, U. T. Glycine oxidase based high-throughput solid-phase assay for substrate profiling and directed evolution of (*R*)- and (*S*)-selective amine transaminases. *Analytical Chemistry* 86, 11847–11853; 10.1021/ac503445y (2014).
16. Bravo, J. *et al.* Crystal structure of catalase HPII from *Escherichia coli*. *Structure* 3, 491–502; 10.1016/s0969-2126(01)00182-4 (1995).

17. Bagnyukova, T. V., Vasylkiv, O. Y., Storey, K. B. & Lushchak, V. I. Catalase inhibition by amino triazole induces oxidative stress in goldfish brain. *Brain Research* 1052, 180–186; 10.1016/j.brainres.2005.06.002 (2005).

18. Margoliash, E. & Novogrodsky, A. A study of the inhibition of catalase by 3-amino-1:2:4:-triazole. *The Biochemical Journal* 68, 468–475; 10.1042/bj0680468 (1958).

19. Walton, P. A. & Pizzitelli, M. Effects of peroxisomal catalase inhibition on mitochondrial function. *Frontiers in Physiology* 3, 108; 10.3389/fphys.2012.00108 (2012).

20. Allgood, G. S. & Perry, J. J. Characterization of a manganese-containing catalase from the obligate thermophile *Thermoleophilum album*. *Journal of Bacteriology* 168, 563–567; 10.1128/jb.168.2.563-567.1986 (1986).
