# Supplementary material for: Substrate scope expansion of 4-phenol oxidases by rational enzyme selection and sequence-function relations
Source: Commun Chem. 2024 Jun 3;7:123. doi: 10.1038/s42004-024-01207-1 (PMC11148156; doi:10.1038/s42004-024-01207-1)
Supplement: Supplementary file 3 — Description of Additional Supplementary Files [file 42004_2024_1207_MOESM3_ESM.pdf]

Description of Additional Supplementary Files

File name- Supplementary Data 01:

File description- Phylogenetic analysis. Raw data for Figures 3, and S1 to S4 including the multiple sequence alignment and the phylogenetic tree

File name- Supplementary Data 02:

File description- Sequence-activity correlations. Raw data for Figures 4, and S26 to S28

File name- Supplementary Data 03:

File description- R code for correlation. Computer code for automatized correlation calculation and individual correlation plots

File name- Supplementary Data 04:

File description- Primary data for point diagrams

File name- Supplementary Data 05:

File description- Primary data for thermal shift assay
