# Supplementary figures and images for: Substrate scope expansion of 4-phenol oxidases by rational enzyme selection and sequence-function relations (part 2 of 2)
Source: Commun Chem. 2024 Jun 3;7:123. doi: 10.1038/s42004-024-01207-1 (PMC11148156; doi:10.1038/s42004-024-01207-1)

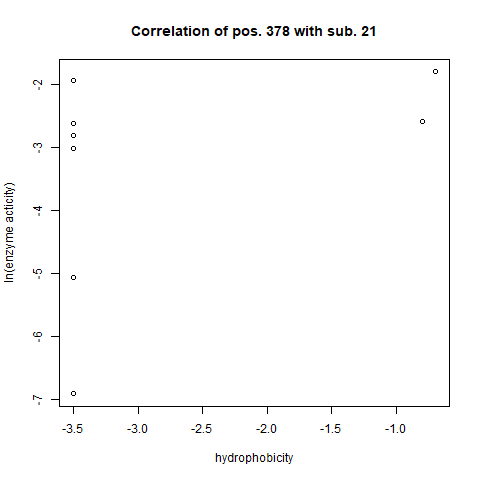

Supplement: Supplementary file 6 — Supplementary Data 3 [file 42004_2024_1207_MOESM6_ESM.zip › Supplementary Data 3/plots/hydrophobicity - 378 - 21 .png]

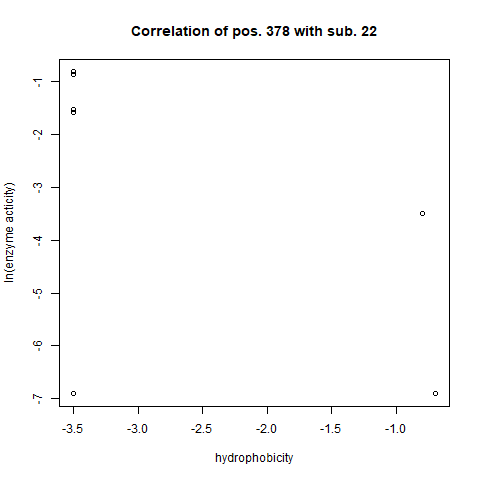

Supplement: Supplementary file 6 — Supplementary Data 3 [file 42004_2024_1207_MOESM6_ESM.zip › Supplementary Data 3/plots/hydrophobicity - 378 - 22 .png]

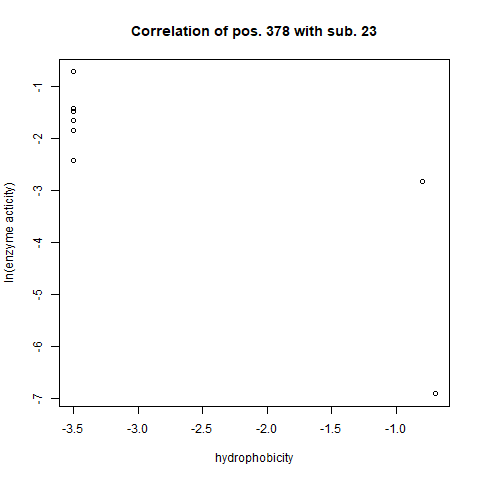

Supplement: Supplementary file 6 — Supplementary Data 3 [file 42004_2024_1207_MOESM6_ESM.zip › Supplementary Data 3/plots/hydrophobicity - 378 - 23 .png]

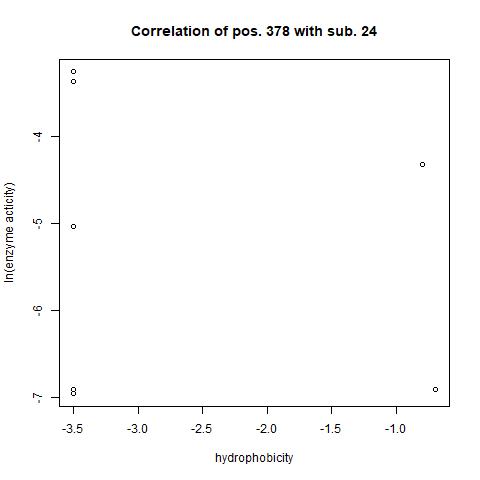

Supplement: Supplementary file 6 — Supplementary Data 3 [file 42004_2024_1207_MOESM6_ESM.zip › Supplementary Data 3/plots/hydrophobicity - 378 - 24 .png]

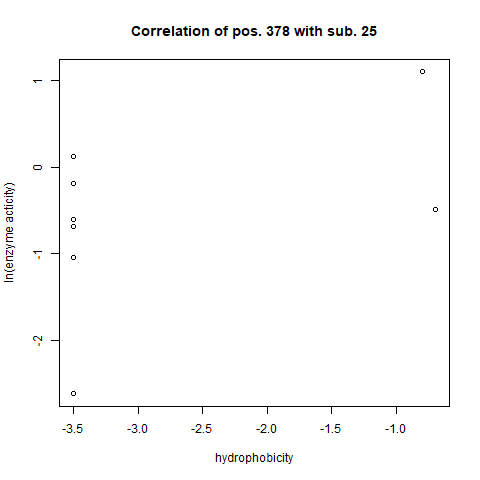

Supplement: Supplementary file 6 — Supplementary Data 3 [file 42004_2024_1207_MOESM6_ESM.zip › Supplementary Data 3/plots/hydrophobicity - 378 - 25 .png]

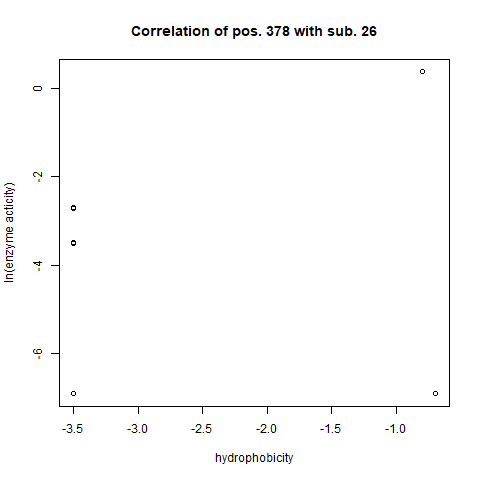

Supplement: Supplementary file 6 — Supplementary Data 3 [file 42004_2024_1207_MOESM6_ESM.zip › Supplementary Data 3/plots/hydrophobicity - 378 - 26 .png]

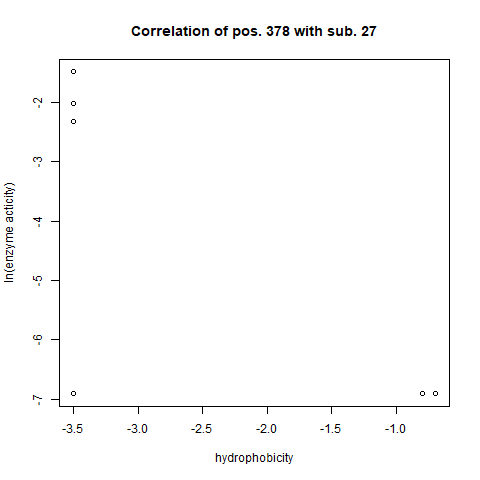

Supplement: Supplementary file 6 — Supplementary Data 3 [file 42004_2024_1207_MOESM6_ESM.zip › Supplementary Data 3/plots/hydrophobicity - 378 - 27 .png]

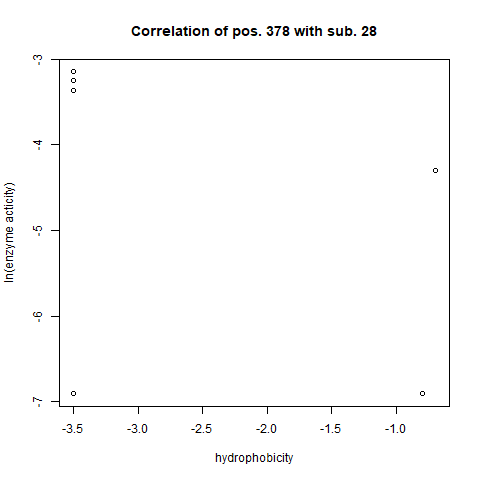

Supplement: Supplementary file 6 — Supplementary Data 3 [file 42004_2024_1207_MOESM6_ESM.zip › Supplementary Data 3/plots/hydrophobicity - 378 - 28 .png]

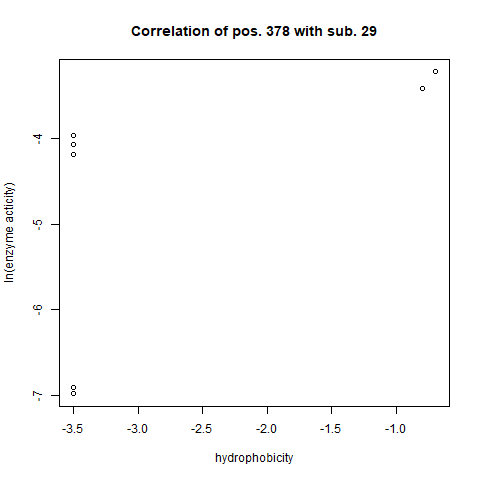

Supplement: Supplementary file 6 — Supplementary Data 3 [file 42004_2024_1207_MOESM6_ESM.zip › Supplementary Data 3/plots/hydrophobicity - 378 - 29 .png]

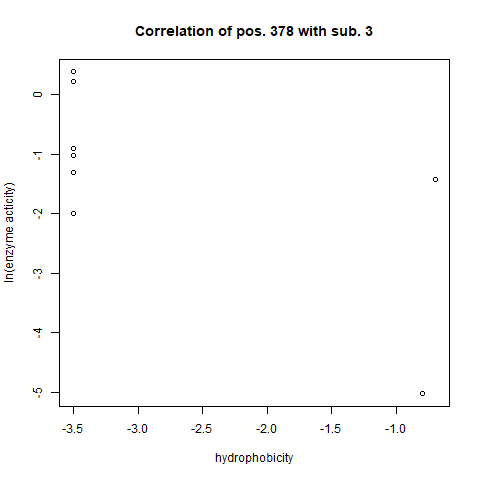

Supplement: Supplementary file 6 — Supplementary Data 3 [file 42004_2024_1207_MOESM6_ESM.zip › Supplementary Data 3/plots/hydrophobicity - 378 - 3 .png]

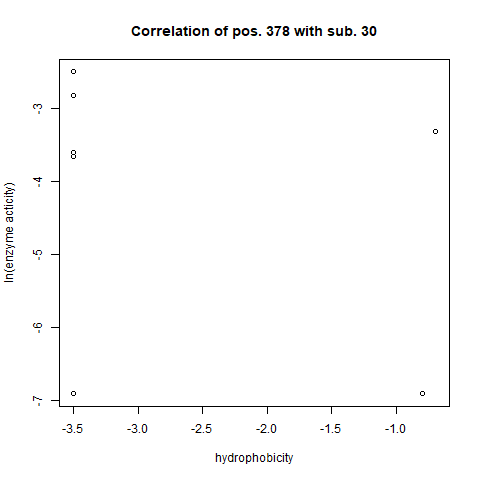

Supplement: Supplementary file 6 — Supplementary Data 3 [file 42004_2024_1207_MOESM6_ESM.zip › Supplementary Data 3/plots/hydrophobicity - 378 - 30 .png]

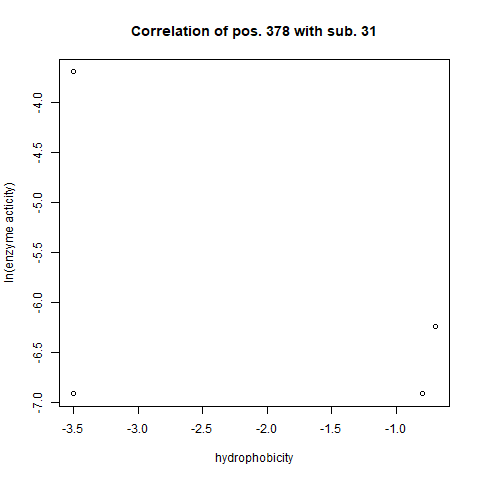

Supplement: Supplementary file 6 — Supplementary Data 3 [file 42004_2024_1207_MOESM6_ESM.zip › Supplementary Data 3/plots/hydrophobicity - 378 - 31 .png]

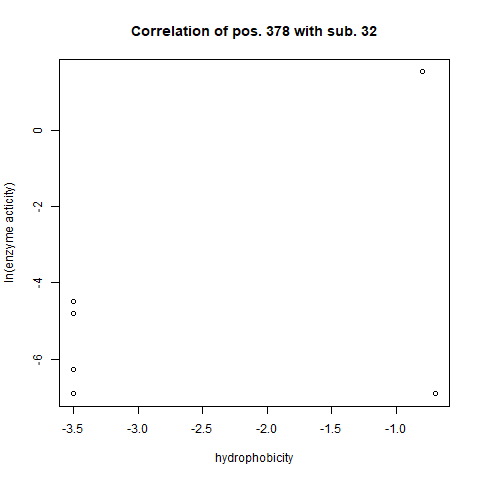

Supplement: Supplementary file 6 — Supplementary Data 3 [file 42004_2024_1207_MOESM6_ESM.zip › Supplementary Data 3/plots/hydrophobicity - 378 - 32 .png]

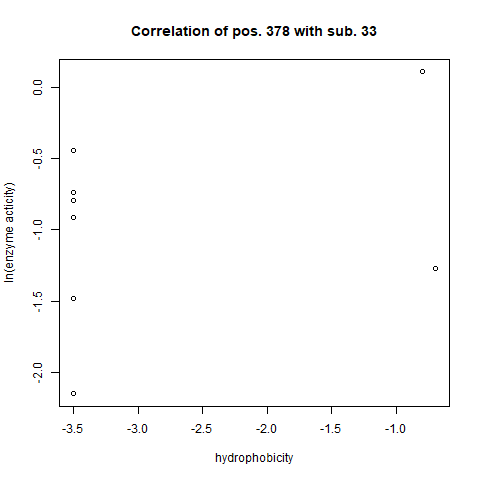

Supplement: Supplementary file 6 — Supplementary Data 3 [file 42004_2024_1207_MOESM6_ESM.zip › Supplementary Data 3/plots/hydrophobicity - 378 - 33 .png]

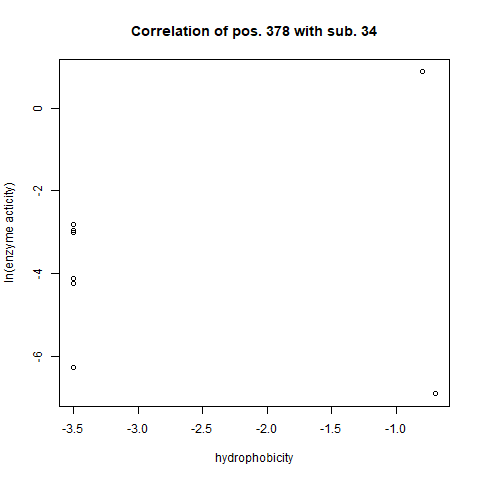

Supplement: Supplementary file 6 — Supplementary Data 3 [file 42004_2024_1207_MOESM6_ESM.zip › Supplementary Data 3/plots/hydrophobicity - 378 - 34 .png]

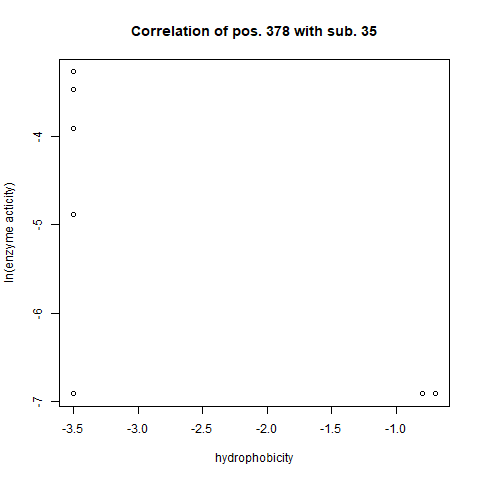

Supplement: Supplementary file 6 — Supplementary Data 3 [file 42004_2024_1207_MOESM6_ESM.zip › Supplementary Data 3/plots/hydrophobicity - 378 - 35 .png]

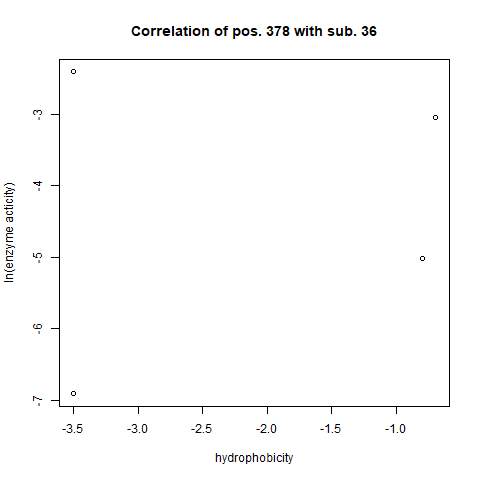

Supplement: Supplementary file 6 — Supplementary Data 3 [file 42004_2024_1207_MOESM6_ESM.zip › Supplementary Data 3/plots/hydrophobicity - 378 - 36 .png]

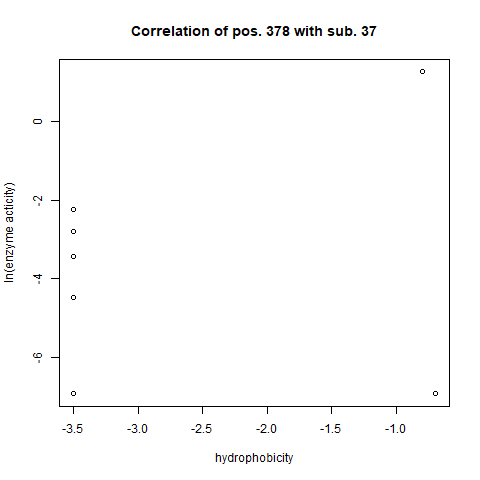

Supplement: Supplementary file 6 — Supplementary Data 3 [file 42004_2024_1207_MOESM6_ESM.zip › Supplementary Data 3/plots/hydrophobicity - 378 - 37 .png]

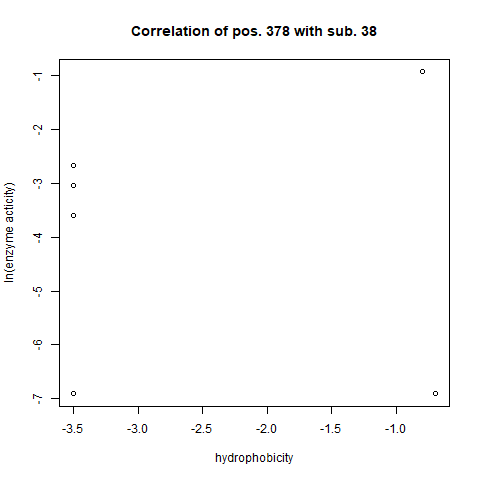

Supplement: Supplementary file 6 — Supplementary Data 3 [file 42004_2024_1207_MOESM6_ESM.zip › Supplementary Data 3/plots/hydrophobicity - 378 - 38 .png]

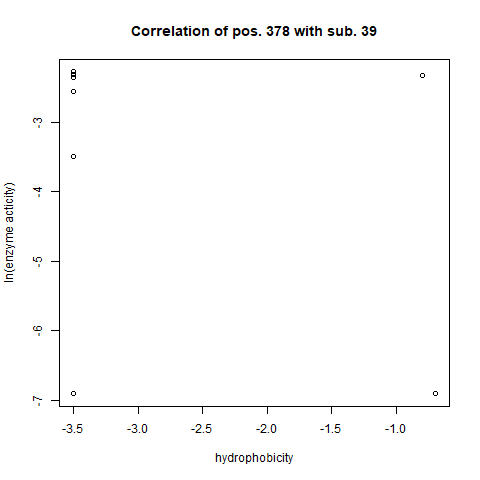

Supplement: Supplementary file 6 — Supplementary Data 3 [file 42004_2024_1207_MOESM6_ESM.zip › Supplementary Data 3/plots/hydrophobicity - 378 - 39 .png]

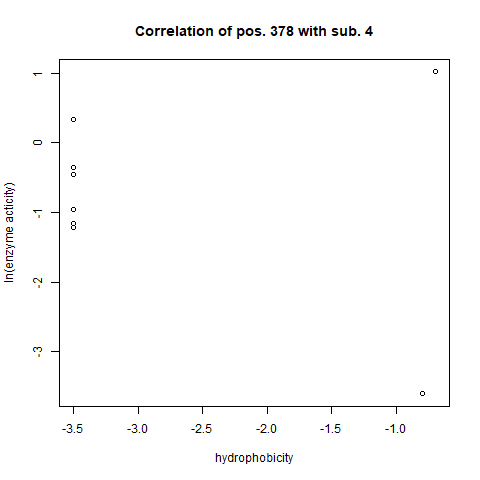

Supplement: Supplementary file 6 — Supplementary Data 3 [file 42004_2024_1207_MOESM6_ESM.zip › Supplementary Data 3/plots/hydrophobicity - 378 - 4 .png]

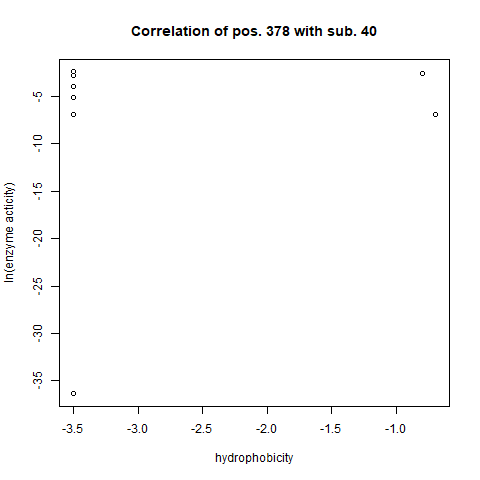

Supplement: Supplementary file 6 — Supplementary Data 3 [file 42004_2024_1207_MOESM6_ESM.zip › Supplementary Data 3/plots/hydrophobicity - 378 - 40 .png]

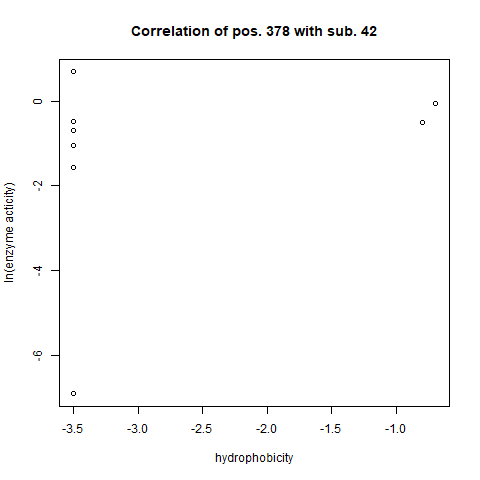

Supplement: Supplementary file 6 — Supplementary Data 3 [file 42004_2024_1207_MOESM6_ESM.zip › Supplementary Data 3/plots/hydrophobicity - 378 - 42 .png]

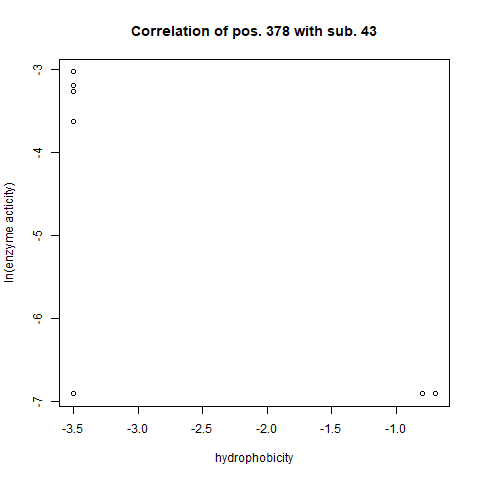

Supplement: Supplementary file 6 — Supplementary Data 3 [file 42004_2024_1207_MOESM6_ESM.zip › Supplementary Data 3/plots/hydrophobicity - 378 - 43 .png]

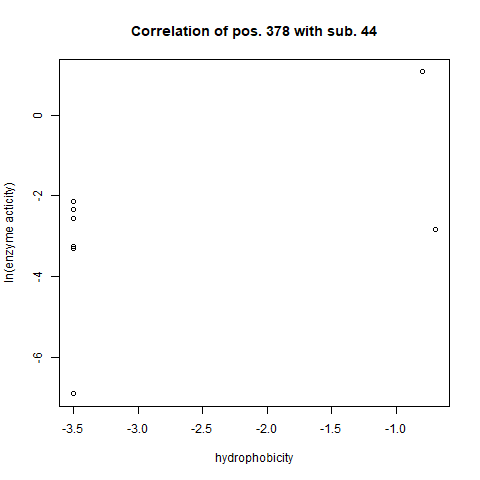

Supplement: Supplementary file 6 — Supplementary Data 3 [file 42004_2024_1207_MOESM6_ESM.zip › Supplementary Data 3/plots/hydrophobicity - 378 - 44 .png]

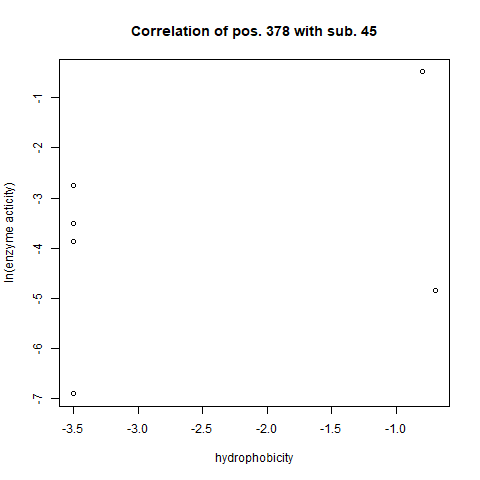

Supplement: Supplementary file 6 — Supplementary Data 3 [file 42004_2024_1207_MOESM6_ESM.zip › Supplementary Data 3/plots/hydrophobicity - 378 - 45 .png]

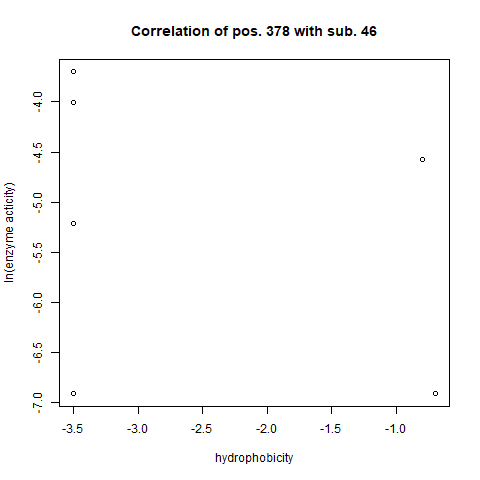

Supplement: Supplementary file 6 — Supplementary Data 3 [file 42004_2024_1207_MOESM6_ESM.zip › Supplementary Data 3/plots/hydrophobicity - 378 - 46 .png]

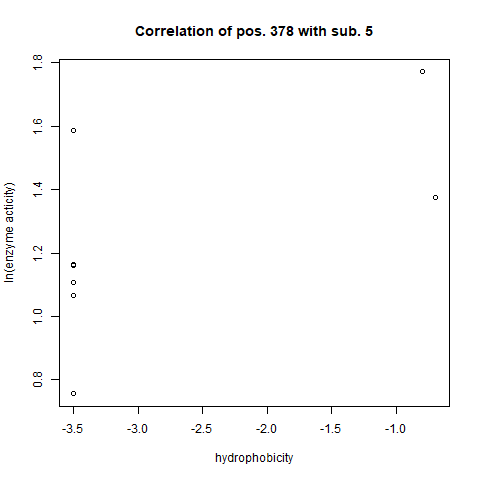

Supplement: Supplementary file 6 — Supplementary Data 3 [file 42004_2024_1207_MOESM6_ESM.zip › Supplementary Data 3/plots/hydrophobicity - 378 - 5 .png]

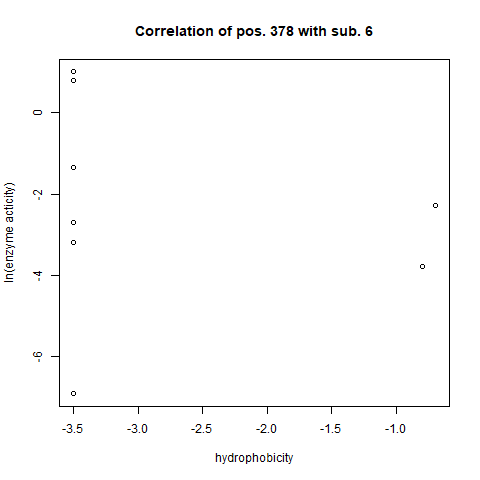

Supplement: Supplementary file 6 — Supplementary Data 3 [file 42004_2024_1207_MOESM6_ESM.zip › Supplementary Data 3/plots/hydrophobicity - 378 - 6 .png]

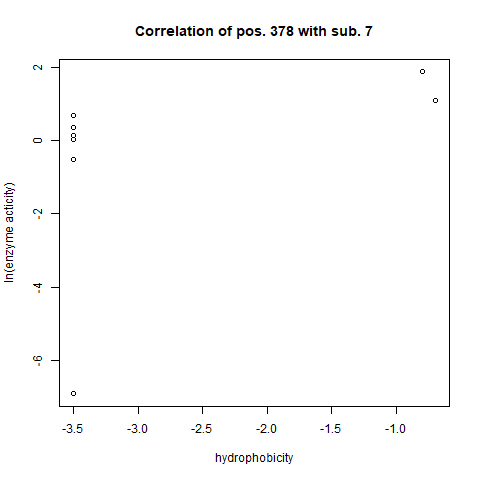

Supplement: Supplementary file 6 — Supplementary Data 3 [file 42004_2024_1207_MOESM6_ESM.zip › Supplementary Data 3/plots/hydrophobicity - 378 - 7 .png]

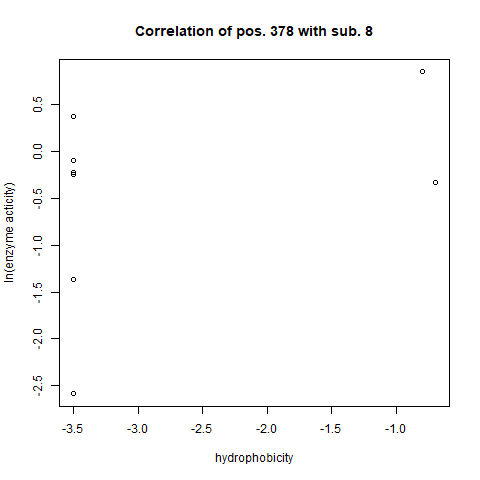

Supplement: Supplementary file 6 — Supplementary Data 3 [file 42004_2024_1207_MOESM6_ESM.zip › Supplementary Data 3/plots/hydrophobicity - 378 - 8 .png]

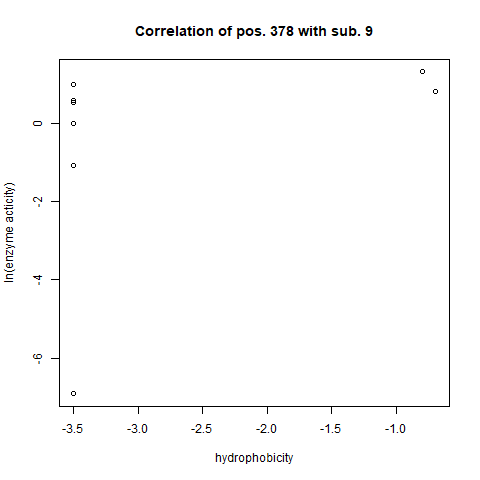

Supplement: Supplementary file 6 — Supplementary Data 3 [file 42004_2024_1207_MOESM6_ESM.zip › Supplementary Data 3/plots/hydrophobicity - 378 - 9 .png]

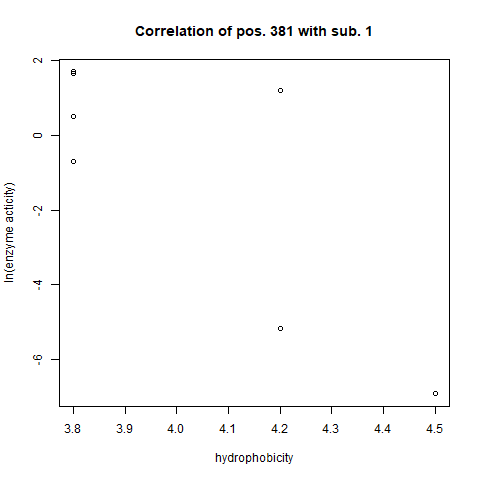

Supplement: Supplementary file 6 — Supplementary Data 3 [file 42004_2024_1207_MOESM6_ESM.zip › Supplementary Data 3/plots/hydrophobicity - 381 - 1 .png]

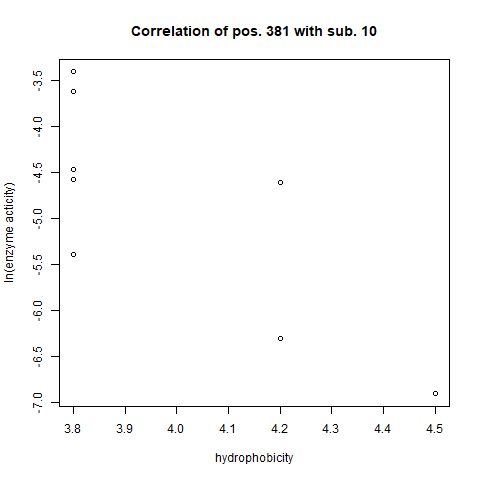

Supplement: Supplementary file 6 — Supplementary Data 3 [file 42004_2024_1207_MOESM6_ESM.zip › Supplementary Data 3/plots/hydrophobicity - 381 - 10 .png]

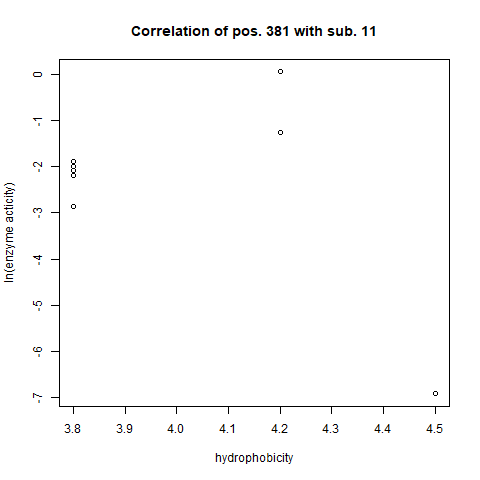

Supplement: Supplementary file 6 — Supplementary Data 3 [file 42004_2024_1207_MOESM6_ESM.zip › Supplementary Data 3/plots/hydrophobicity - 381 - 11 .png]

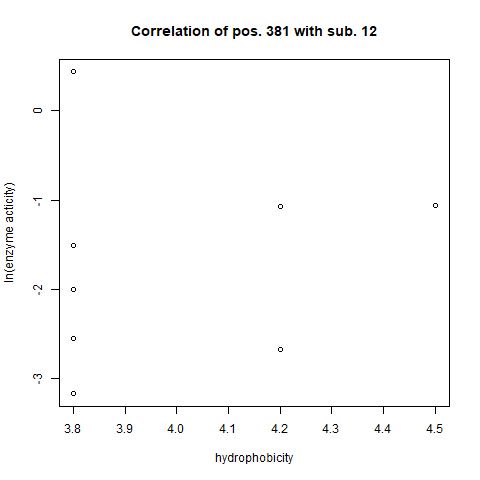

Supplement: Supplementary file 6 — Supplementary Data 3 [file 42004_2024_1207_MOESM6_ESM.zip › Supplementary Data 3/plots/hydrophobicity - 381 - 12 .png]

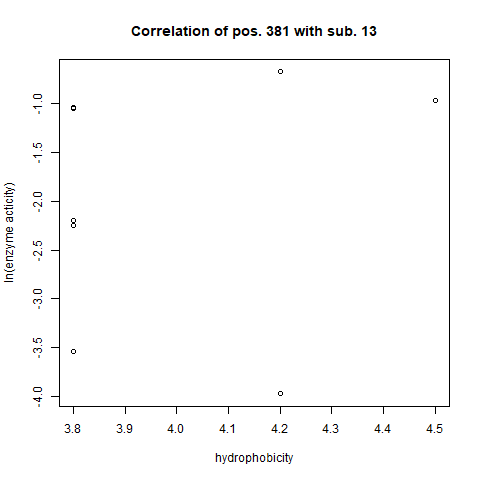

Supplement: Supplementary file 6 — Supplementary Data 3 [file 42004_2024_1207_MOESM6_ESM.zip › Supplementary Data 3/plots/hydrophobicity - 381 - 13 .png]

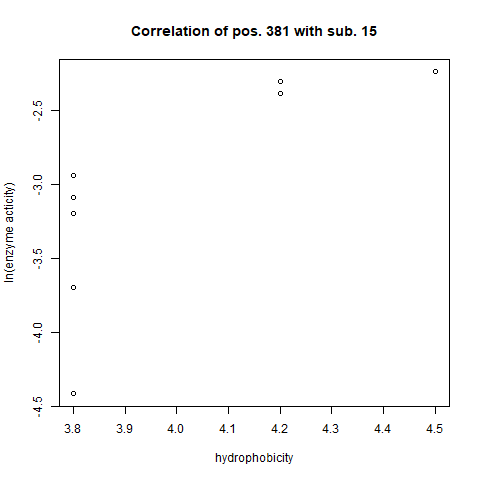

Supplement: Supplementary file 6 — Supplementary Data 3 [file 42004_2024_1207_MOESM6_ESM.zip › Supplementary Data 3/plots/hydrophobicity - 381 - 15 .png]

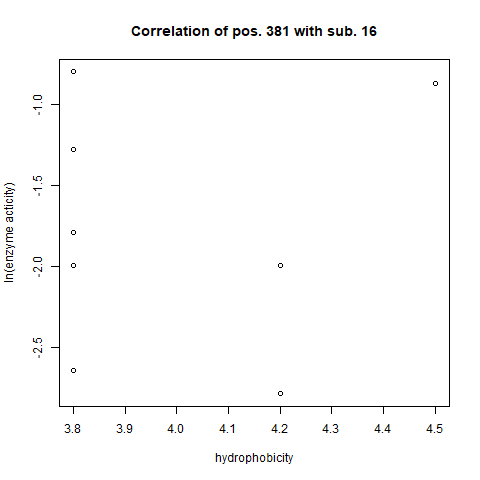

Supplement: Supplementary file 6 — Supplementary Data 3 [file 42004_2024_1207_MOESM6_ESM.zip › Supplementary Data 3/plots/hydrophobicity - 381 - 16 .png]

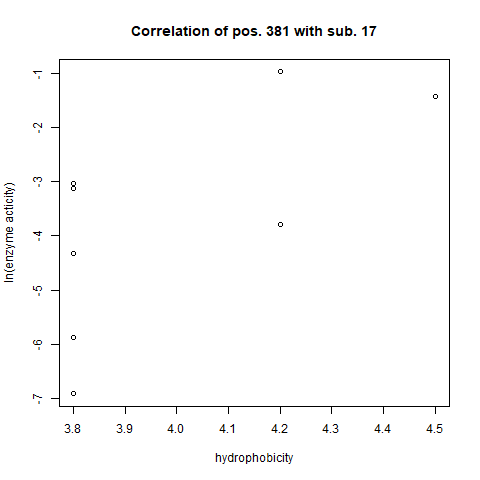

Supplement: Supplementary file 6 — Supplementary Data 3 [file 42004_2024_1207_MOESM6_ESM.zip › Supplementary Data 3/plots/hydrophobicity - 381 - 17 .png]

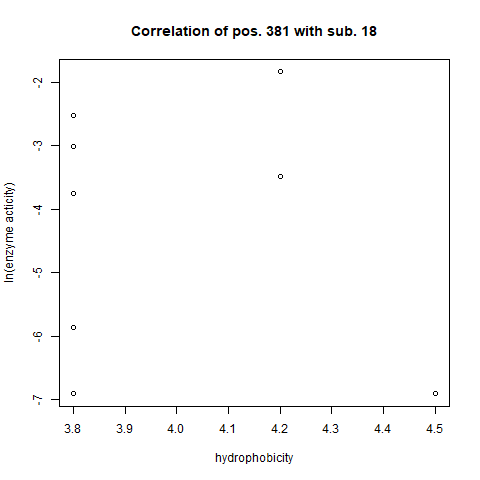

Supplement: Supplementary file 6 — Supplementary Data 3 [file 42004_2024_1207_MOESM6_ESM.zip › Supplementary Data 3/plots/hydrophobicity - 381 - 18 .png]

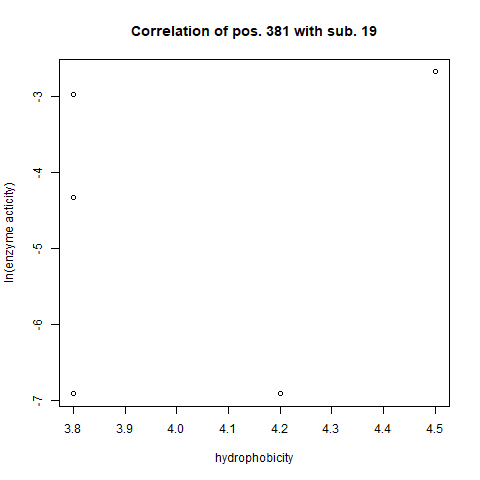

Supplement: Supplementary file 6 — Supplementary Data 3 [file 42004_2024_1207_MOESM6_ESM.zip › Supplementary Data 3/plots/hydrophobicity - 381 - 19 .png]

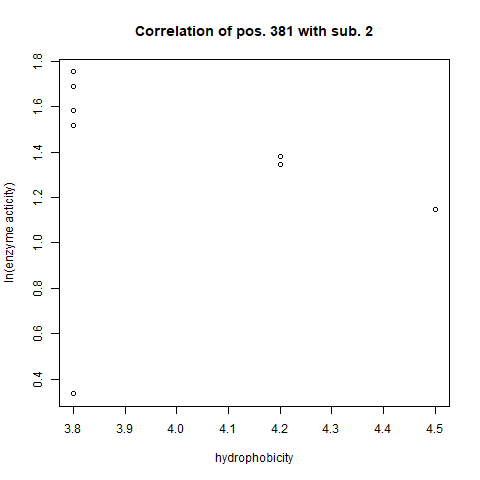

Supplement: Supplementary file 6 — Supplementary Data 3 [file 42004_2024_1207_MOESM6_ESM.zip › Supplementary Data 3/plots/hydrophobicity - 381 - 2 .png]

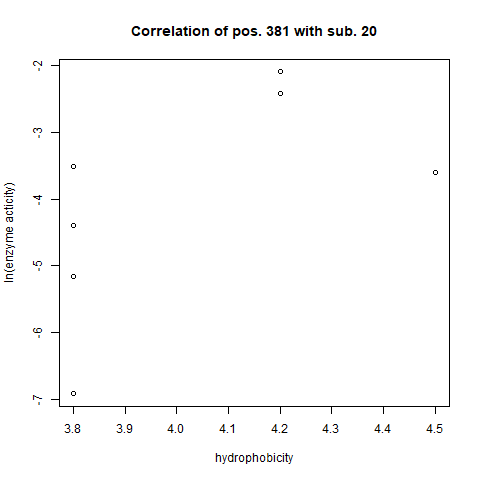

Supplement: Supplementary file 6 — Supplementary Data 3 [file 42004_2024_1207_MOESM6_ESM.zip › Supplementary Data 3/plots/hydrophobicity - 381 - 20 .png]

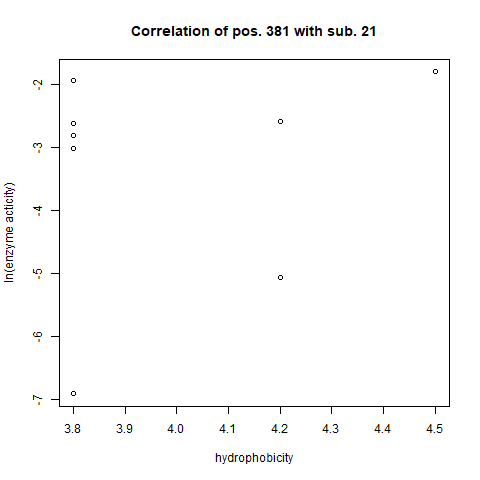

Supplement: Supplementary file 6 — Supplementary Data 3 [file 42004_2024_1207_MOESM6_ESM.zip › Supplementary Data 3/plots/hydrophobicity - 381 - 21 .png]

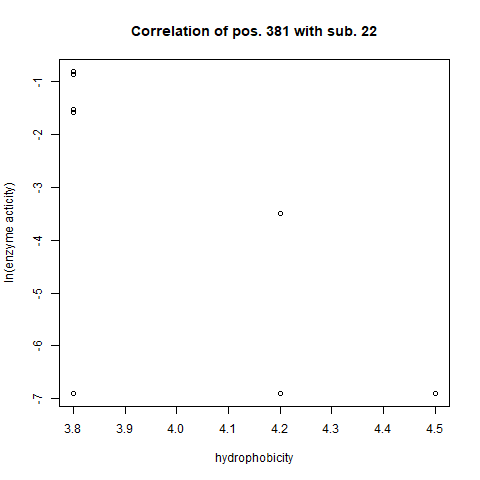

Supplement: Supplementary file 6 — Supplementary Data 3 [file 42004_2024_1207_MOESM6_ESM.zip › Supplementary Data 3/plots/hydrophobicity - 381 - 22 .png]

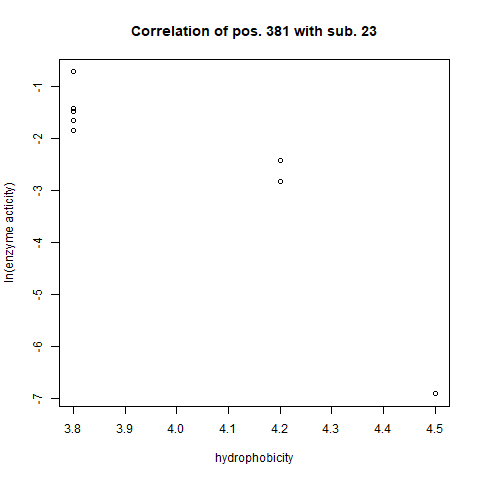

Supplement: Supplementary file 6 — Supplementary Data 3 [file 42004_2024_1207_MOESM6_ESM.zip › Supplementary Data 3/plots/hydrophobicity - 381 - 23 .png]

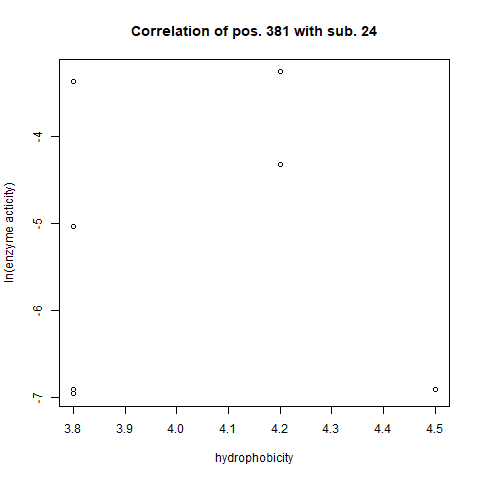

Supplement: Supplementary file 6 — Supplementary Data 3 [file 42004_2024_1207_MOESM6_ESM.zip › Supplementary Data 3/plots/hydrophobicity - 381 - 24 .png]

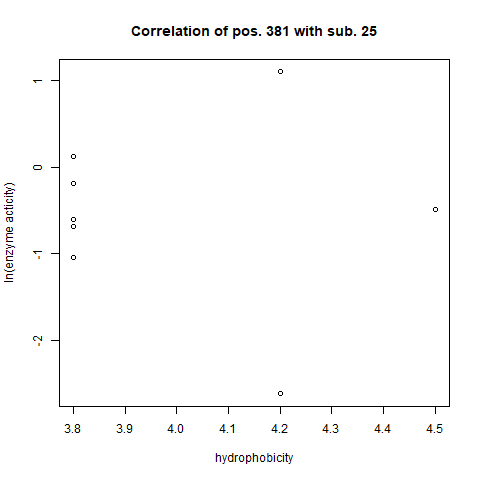

Supplement: Supplementary file 6 — Supplementary Data 3 [file 42004_2024_1207_MOESM6_ESM.zip › Supplementary Data 3/plots/hydrophobicity - 381 - 25 .png]

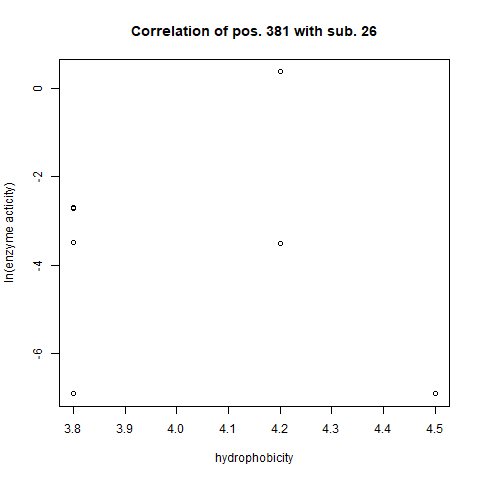

Supplement: Supplementary file 6 — Supplementary Data 3 [file 42004_2024_1207_MOESM6_ESM.zip › Supplementary Data 3/plots/hydrophobicity - 381 - 26 .png]

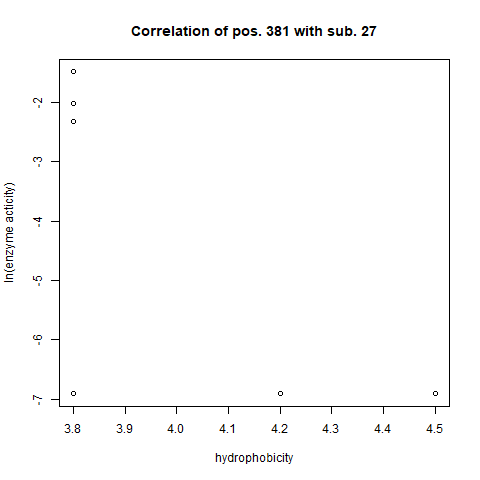

Supplement: Supplementary file 6 — Supplementary Data 3 [file 42004_2024_1207_MOESM6_ESM.zip › Supplementary Data 3/plots/hydrophobicity - 381 - 27 .png]

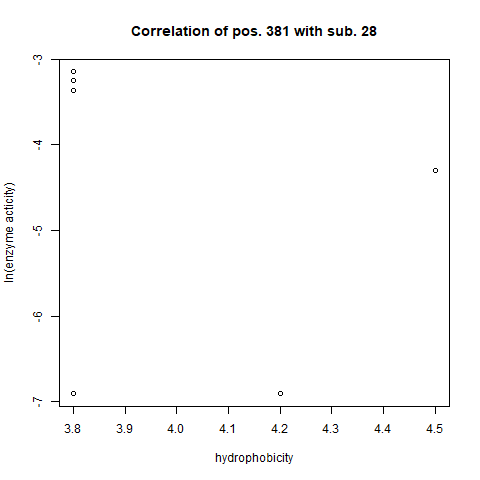

Supplement: Supplementary file 6 — Supplementary Data 3 [file 42004_2024_1207_MOESM6_ESM.zip › Supplementary Data 3/plots/hydrophobicity - 381 - 28 .png]

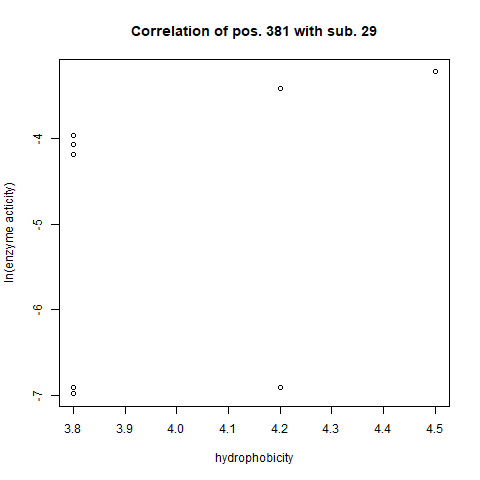

Supplement: Supplementary file 6 — Supplementary Data 3 [file 42004_2024_1207_MOESM6_ESM.zip › Supplementary Data 3/plots/hydrophobicity - 381 - 29 .png]

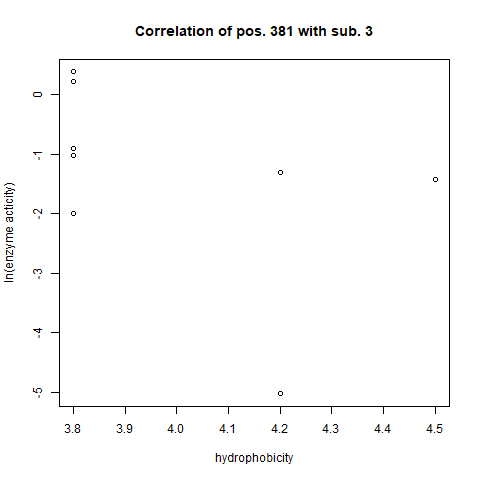

Supplement: Supplementary file 6 — Supplementary Data 3 [file 42004_2024_1207_MOESM6_ESM.zip › Supplementary Data 3/plots/hydrophobicity - 381 - 3 .png]

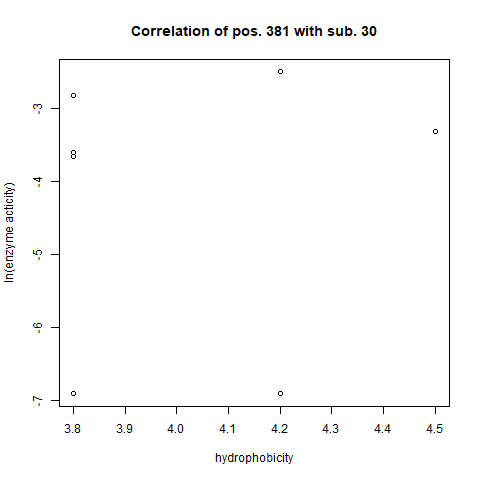

Supplement: Supplementary file 6 — Supplementary Data 3 [file 42004_2024_1207_MOESM6_ESM.zip › Supplementary Data 3/plots/hydrophobicity - 381 - 30 .png]

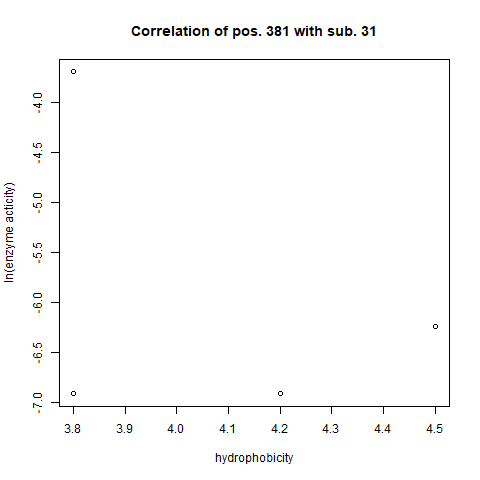

Supplement: Supplementary file 6 — Supplementary Data 3 [file 42004_2024_1207_MOESM6_ESM.zip › Supplementary Data 3/plots/hydrophobicity - 381 - 31 .png]

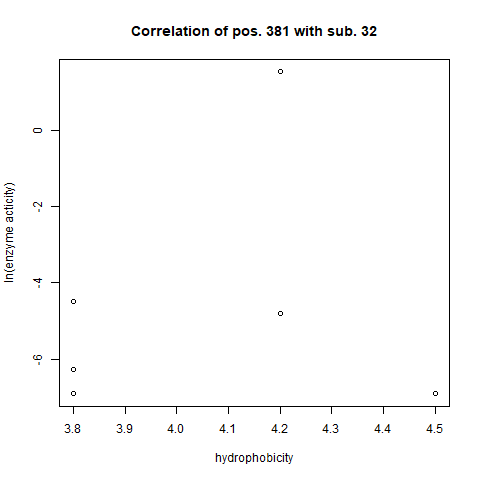

Supplement: Supplementary file 6 — Supplementary Data 3 [file 42004_2024_1207_MOESM6_ESM.zip › Supplementary Data 3/plots/hydrophobicity - 381 - 32 .png]

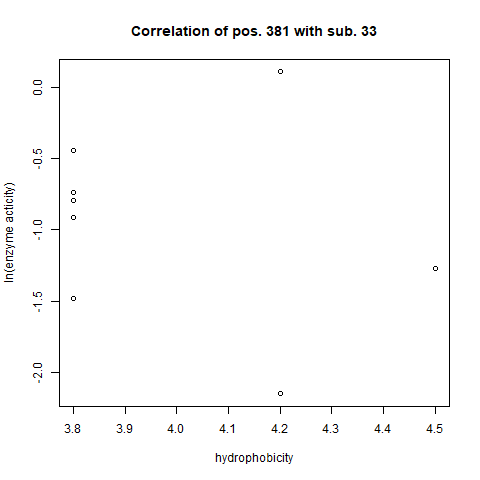

Supplement: Supplementary file 6 — Supplementary Data 3 [file 42004_2024_1207_MOESM6_ESM.zip › Supplementary Data 3/plots/hydrophobicity - 381 - 33 .png]

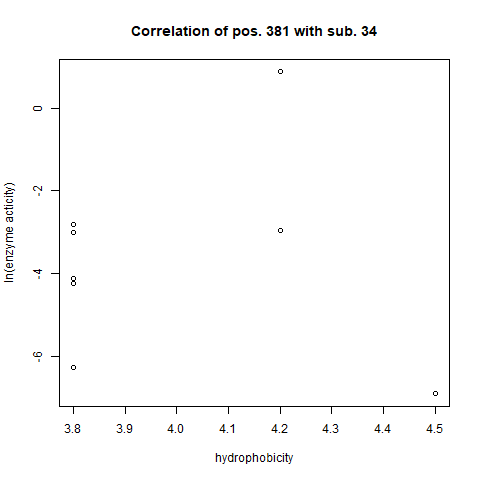

Supplement: Supplementary file 6 — Supplementary Data 3 [file 42004_2024_1207_MOESM6_ESM.zip › Supplementary Data 3/plots/hydrophobicity - 381 - 34 .png]

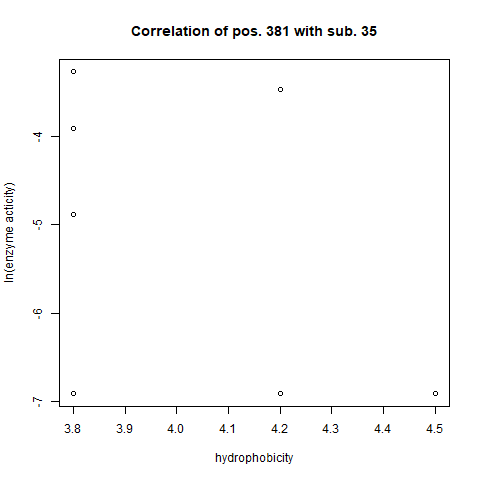

Supplement: Supplementary file 6 — Supplementary Data 3 [file 42004_2024_1207_MOESM6_ESM.zip › Supplementary Data 3/plots/hydrophobicity - 381 - 35 .png]

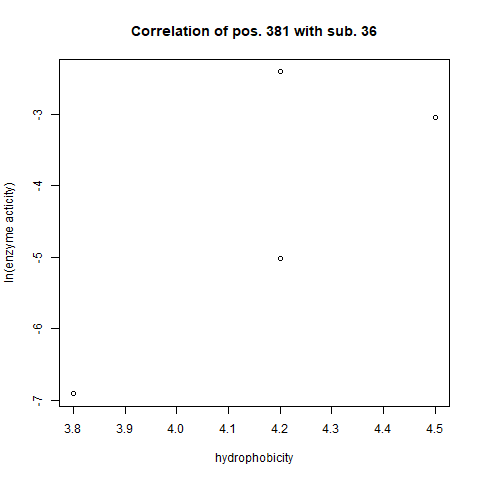

Supplement: Supplementary file 6 — Supplementary Data 3 [file 42004_2024_1207_MOESM6_ESM.zip › Supplementary Data 3/plots/hydrophobicity - 381 - 36 .png]

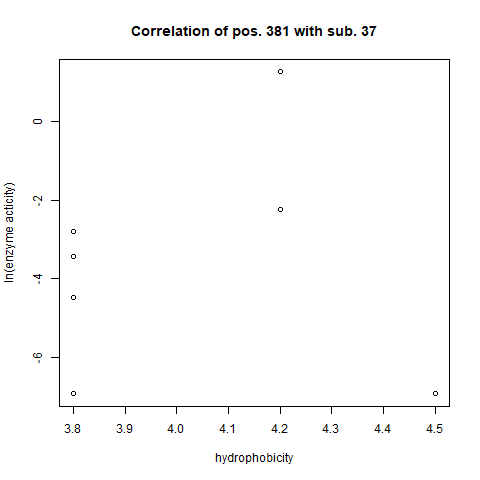

Supplement: Supplementary file 6 — Supplementary Data 3 [file 42004_2024_1207_MOESM6_ESM.zip › Supplementary Data 3/plots/hydrophobicity - 381 - 37 .png]

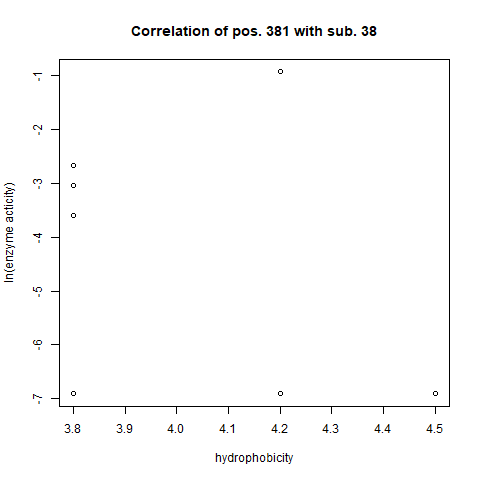

Supplement: Supplementary file 6 — Supplementary Data 3 [file 42004_2024_1207_MOESM6_ESM.zip › Supplementary Data 3/plots/hydrophobicity - 381 - 38 .png]

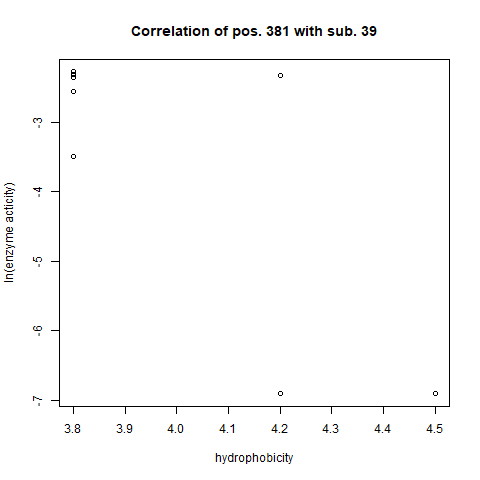

Supplement: Supplementary file 6 — Supplementary Data 3 [file 42004_2024_1207_MOESM6_ESM.zip › Supplementary Data 3/plots/hydrophobicity - 381 - 39 .png]

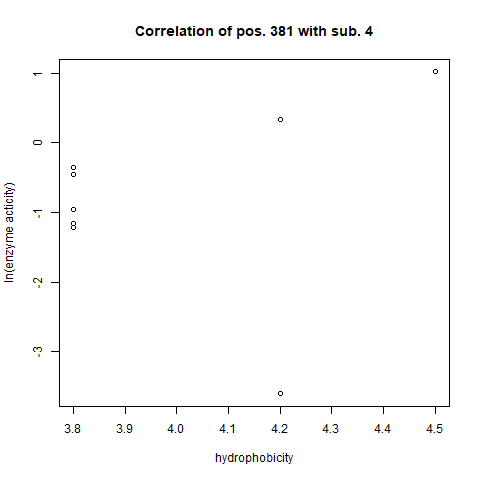

Supplement: Supplementary file 6 — Supplementary Data 3 [file 42004_2024_1207_MOESM6_ESM.zip › Supplementary Data 3/plots/hydrophobicity - 381 - 4 .png]

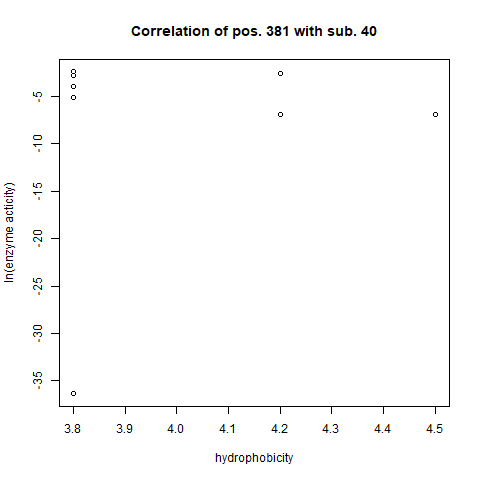

Supplement: Supplementary file 6 — Supplementary Data 3 [file 42004_2024_1207_MOESM6_ESM.zip › Supplementary Data 3/plots/hydrophobicity - 381 - 40 .png]

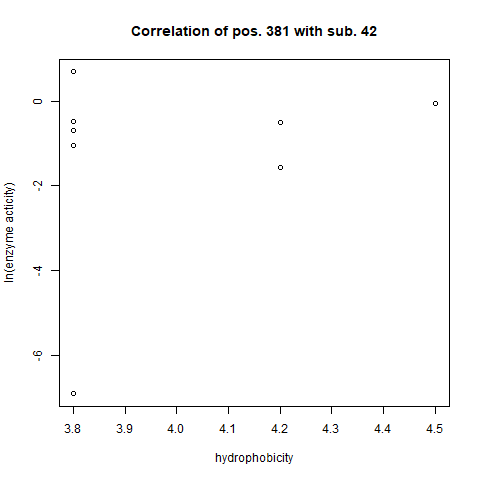

Supplement: Supplementary file 6 — Supplementary Data 3 [file 42004_2024_1207_MOESM6_ESM.zip › Supplementary Data 3/plots/hydrophobicity - 381 - 42 .png]

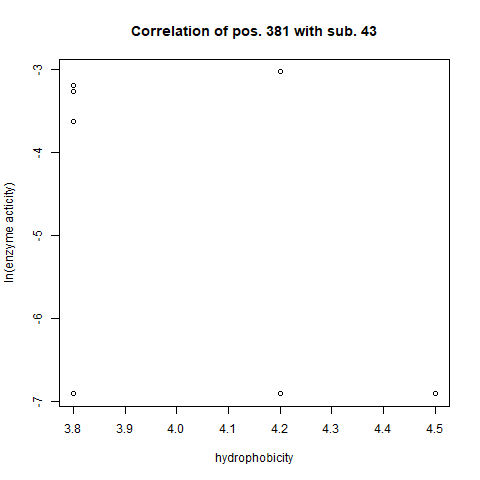

Supplement: Supplementary file 6 — Supplementary Data 3 [file 42004_2024_1207_MOESM6_ESM.zip › Supplementary Data 3/plots/hydrophobicity - 381 - 43 .png]

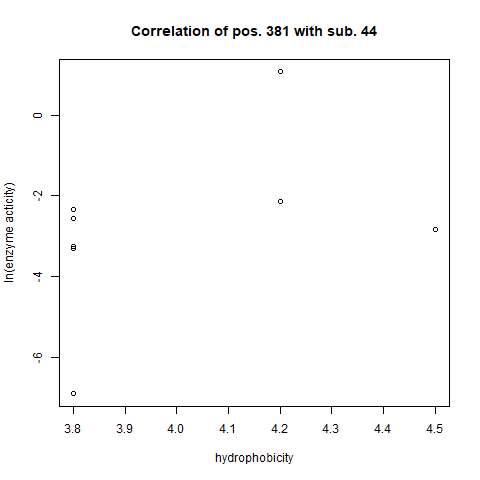

Supplement: Supplementary file 6 — Supplementary Data 3 [file 42004_2024_1207_MOESM6_ESM.zip › Supplementary Data 3/plots/hydrophobicity - 381 - 44 .png]

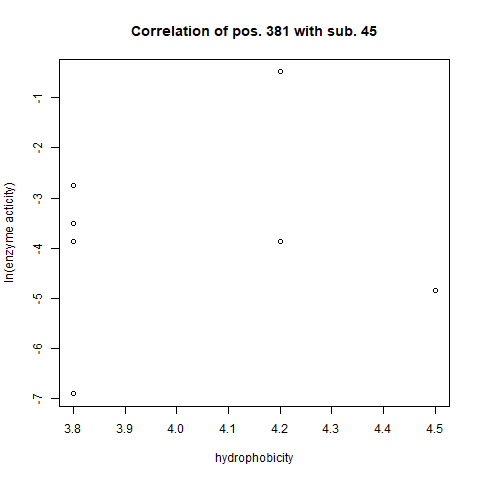

Supplement: Supplementary file 6 — Supplementary Data 3 [file 42004_2024_1207_MOESM6_ESM.zip › Supplementary Data 3/plots/hydrophobicity - 381 - 45 .png]

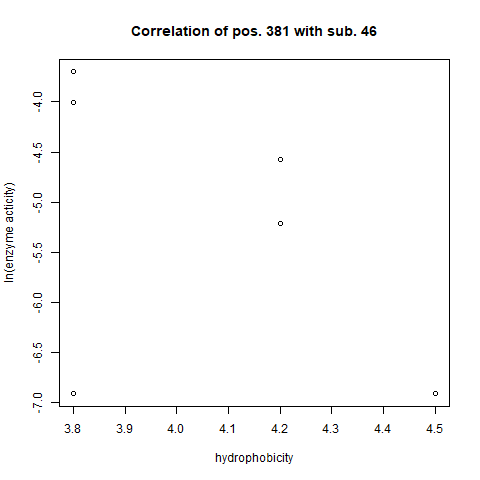

Supplement: Supplementary file 6 — Supplementary Data 3 [file 42004_2024_1207_MOESM6_ESM.zip › Supplementary Data 3/plots/hydrophobicity - 381 - 46 .png]

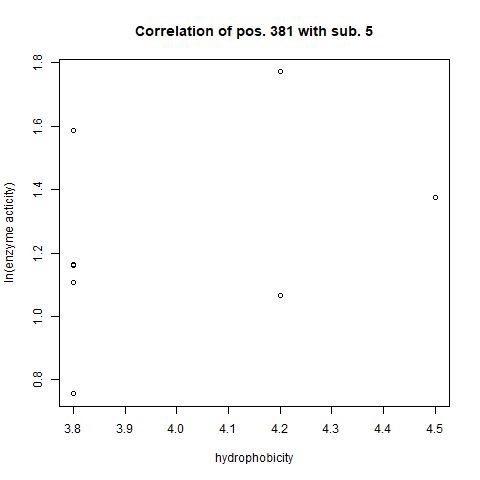

Supplement: Supplementary file 6 — Supplementary Data 3 [file 42004_2024_1207_MOESM6_ESM.zip › Supplementary Data 3/plots/hydrophobicity - 381 - 5 .png]

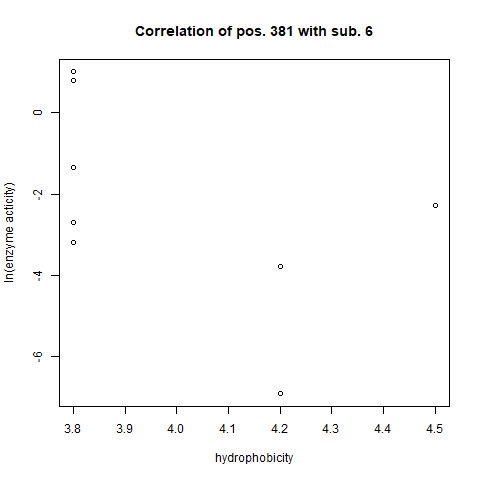

Supplement: Supplementary file 6 — Supplementary Data 3 [file 42004_2024_1207_MOESM6_ESM.zip › Supplementary Data 3/plots/hydrophobicity - 381 - 6 .png]

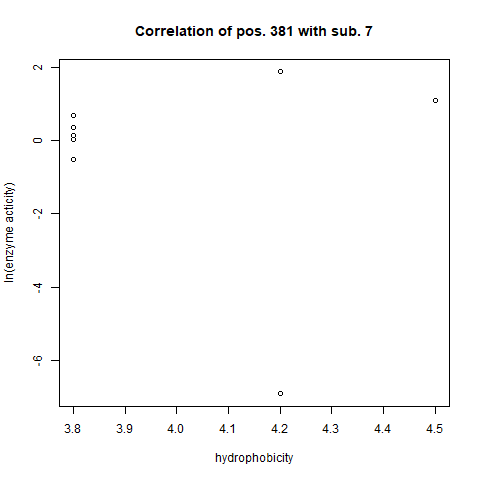

Supplement: Supplementary file 6 — Supplementary Data 3 [file 42004_2024_1207_MOESM6_ESM.zip › Supplementary Data 3/plots/hydrophobicity - 381 - 7 .png]

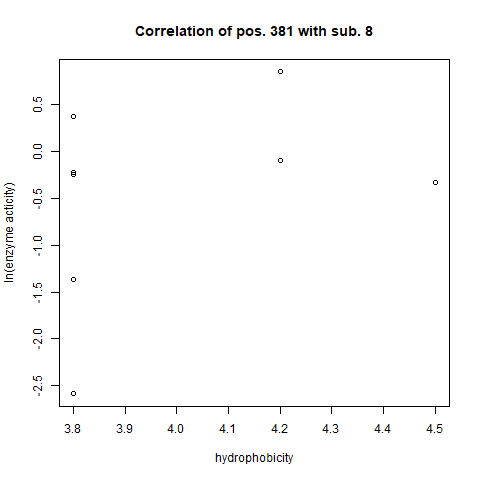

Supplement: Supplementary file 6 — Supplementary Data 3 [file 42004_2024_1207_MOESM6_ESM.zip › Supplementary Data 3/plots/hydrophobicity - 381 - 8 .png]

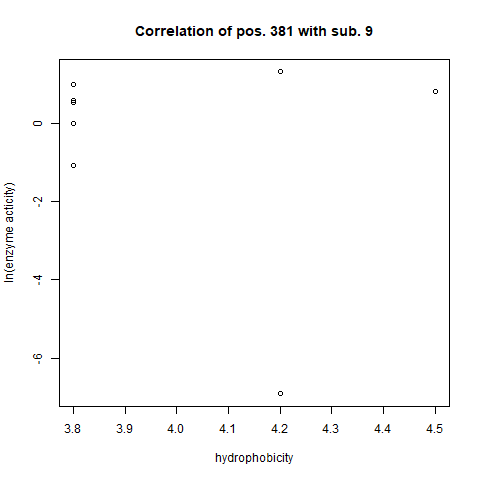

Supplement: Supplementary file 6 — Supplementary Data 3 [file 42004_2024_1207_MOESM6_ESM.zip › Supplementary Data 3/plots/hydrophobicity - 381 - 9 .png]

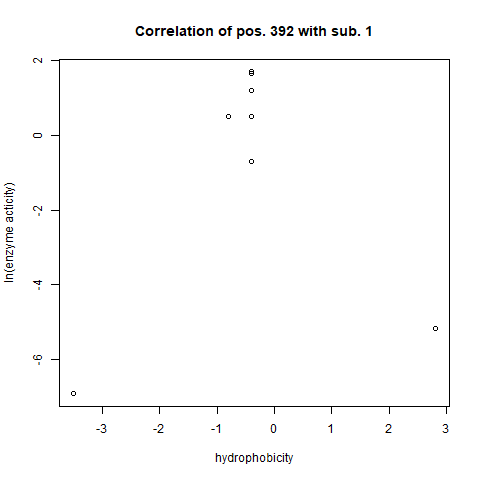

Supplement: Supplementary file 6 — Supplementary Data 3 [file 42004_2024_1207_MOESM6_ESM.zip › Supplementary Data 3/plots/hydrophobicity - 392 - 1 .png]

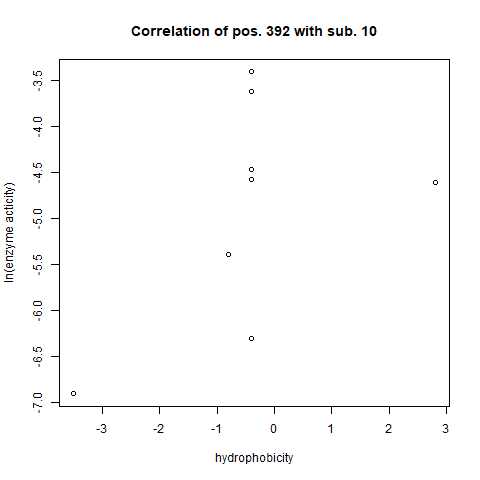

Supplement: Supplementary file 6 — Supplementary Data 3 [file 42004_2024_1207_MOESM6_ESM.zip › Supplementary Data 3/plots/hydrophobicity - 392 - 10 .png]

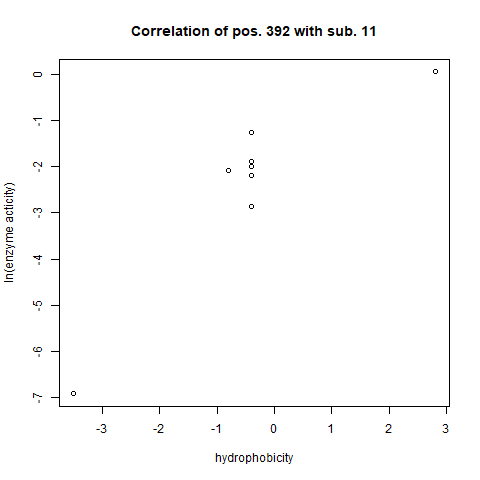

Supplement: Supplementary file 6 — Supplementary Data 3 [file 42004_2024_1207_MOESM6_ESM.zip › Supplementary Data 3/plots/hydrophobicity - 392 - 11 .png]

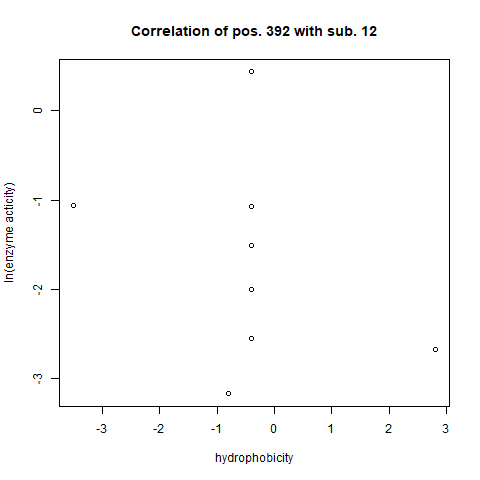

Supplement: Supplementary file 6 — Supplementary Data 3 [file 42004_2024_1207_MOESM6_ESM.zip › Supplementary Data 3/plots/hydrophobicity - 392 - 12 .png]

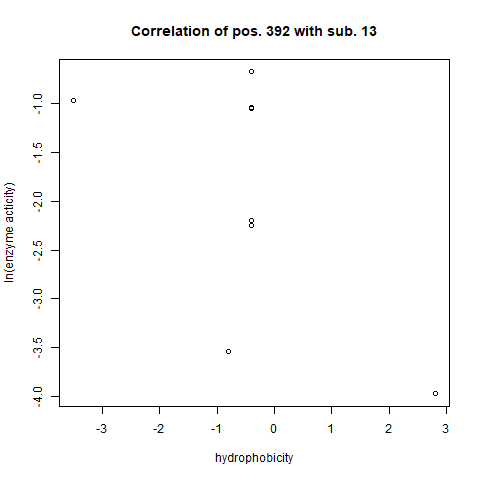

Supplement: Supplementary file 6 — Supplementary Data 3 [file 42004_2024_1207_MOESM6_ESM.zip › Supplementary Data 3/plots/hydrophobicity - 392 - 13 .png]

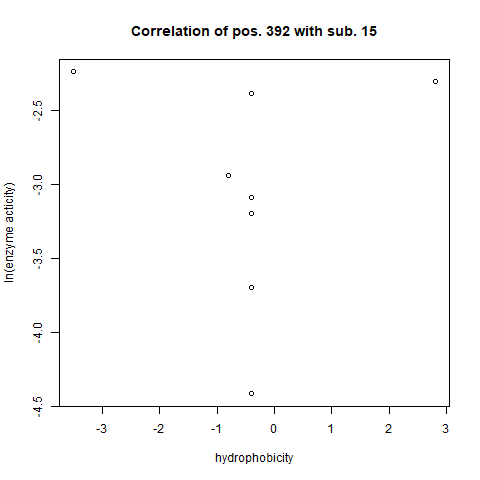

Supplement: Supplementary file 6 — Supplementary Data 3 [file 42004_2024_1207_MOESM6_ESM.zip › Supplementary Data 3/plots/hydrophobicity - 392 - 15 .png]

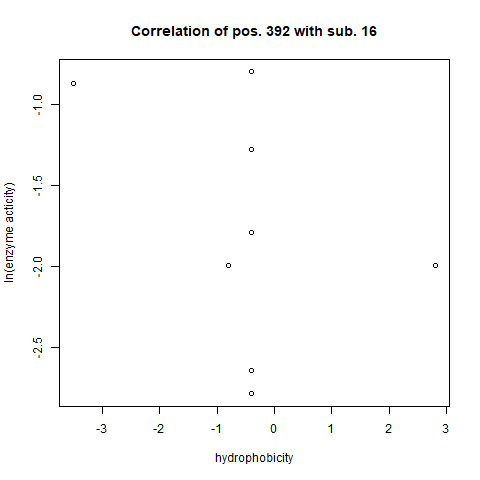

Supplement: Supplementary file 6 — Supplementary Data 3 [file 42004_2024_1207_MOESM6_ESM.zip › Supplementary Data 3/plots/hydrophobicity - 392 - 16 .png]

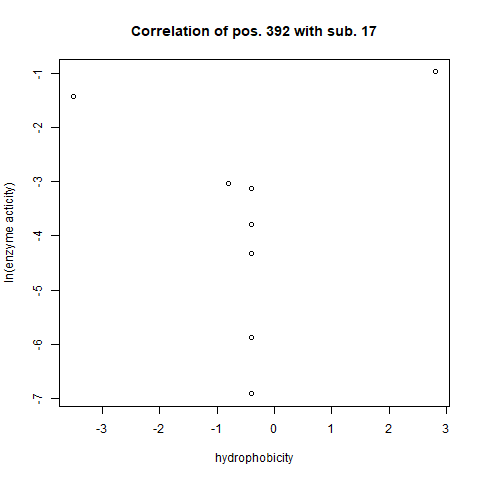

Supplement: Supplementary file 6 — Supplementary Data 3 [file 42004_2024_1207_MOESM6_ESM.zip › Supplementary Data 3/plots/hydrophobicity - 392 - 17 .png]

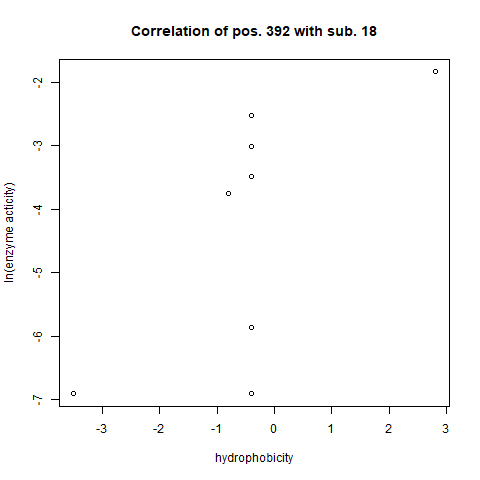

Supplement: Supplementary file 6 — Supplementary Data 3 [file 42004_2024_1207_MOESM6_ESM.zip › Supplementary Data 3/plots/hydrophobicity - 392 - 18 .png]

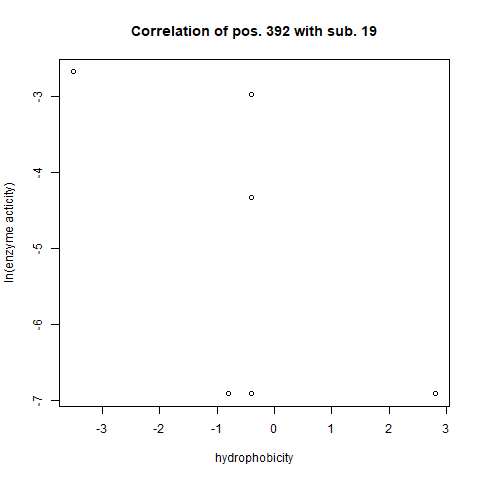

Supplement: Supplementary file 6 — Supplementary Data 3 [file 42004_2024_1207_MOESM6_ESM.zip › Supplementary Data 3/plots/hydrophobicity - 392 - 19 .png]

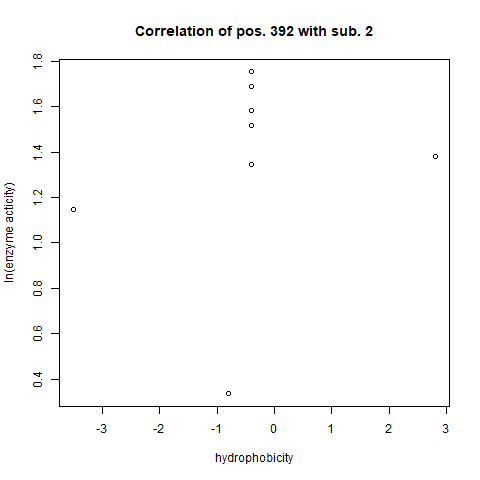

Supplement: Supplementary file 6 — Supplementary Data 3 [file 42004_2024_1207_MOESM6_ESM.zip › Supplementary Data 3/plots/hydrophobicity - 392 - 2 .png]

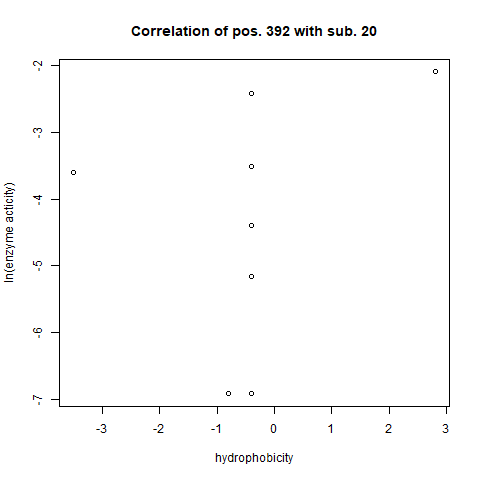

Supplement: Supplementary file 6 — Supplementary Data 3 [file 42004_2024_1207_MOESM6_ESM.zip › Supplementary Data 3/plots/hydrophobicity - 392 - 20 .png]

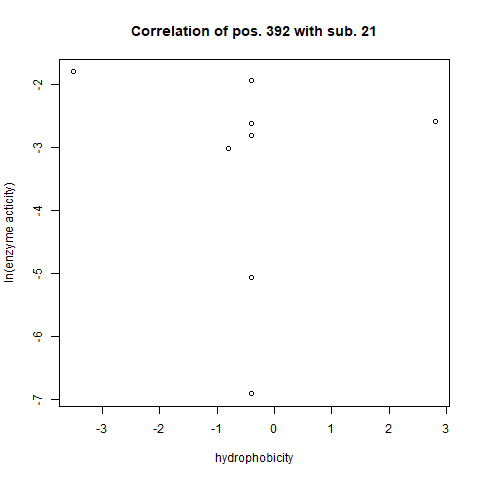

Supplement: Supplementary file 6 — Supplementary Data 3 [file 42004_2024_1207_MOESM6_ESM.zip › Supplementary Data 3/plots/hydrophobicity - 392 - 21 .png]

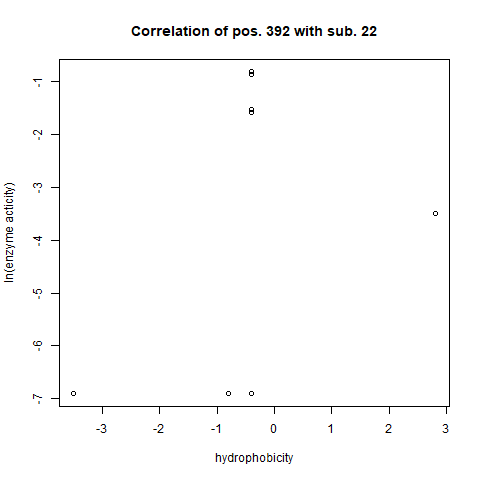

Supplement: Supplementary file 6 — Supplementary Data 3 [file 42004_2024_1207_MOESM6_ESM.zip › Supplementary Data 3/plots/hydrophobicity - 392 - 22 .png]

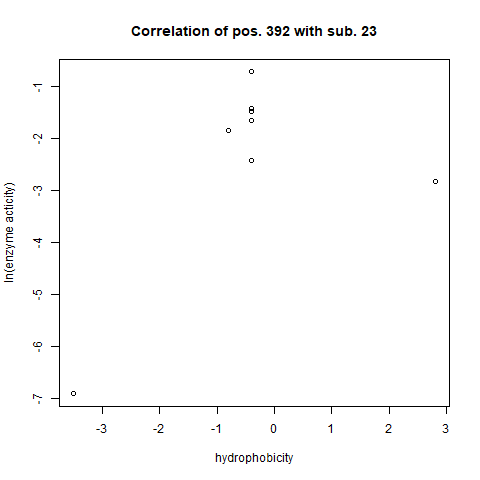

Supplement: Supplementary file 6 — Supplementary Data 3 [file 42004_2024_1207_MOESM6_ESM.zip › Supplementary Data 3/plots/hydrophobicity - 392 - 23 .png]

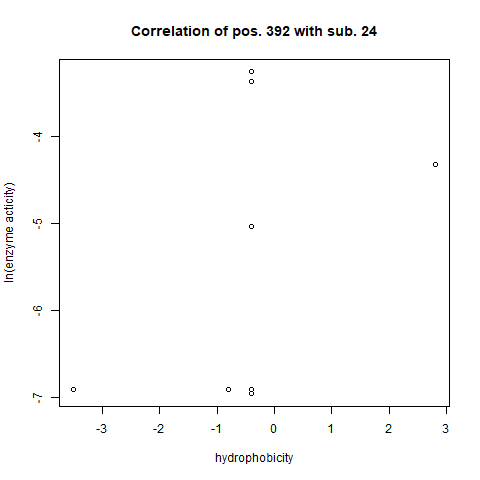

Supplement: Supplementary file 6 — Supplementary Data 3 [file 42004_2024_1207_MOESM6_ESM.zip › Supplementary Data 3/plots/hydrophobicity - 392 - 24 .png]
