# Supplementary figures and images for: Substrate scope expansion of 4-phenol oxidases by rational enzyme selection and sequence-function relations (part 1 of 2)
Source: Commun Chem. 2024 Jun 3;7:123. doi: 10.1038/s42004-024-01207-1 (PMC11148156; doi:10.1038/s42004-024-01207-1)

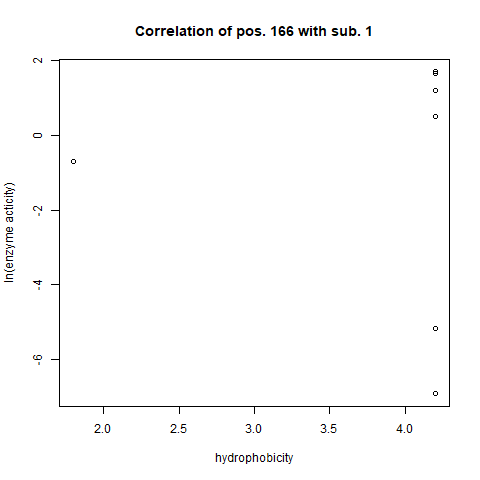

Supplement: Supplementary file 6 — Supplementary Data 3 [file 42004_2024_1207_MOESM6_ESM.zip › Supplementary Data 3/plots/hydrophobicity - 166 - 1 .png]

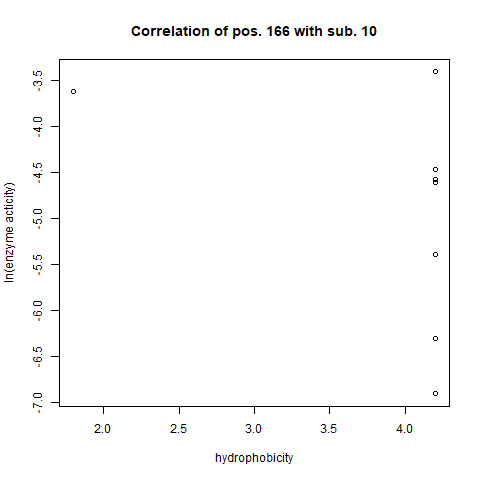

Supplement: Supplementary file 6 — Supplementary Data 3 [file 42004_2024_1207_MOESM6_ESM.zip › Supplementary Data 3/plots/hydrophobicity - 166 - 10 .png]

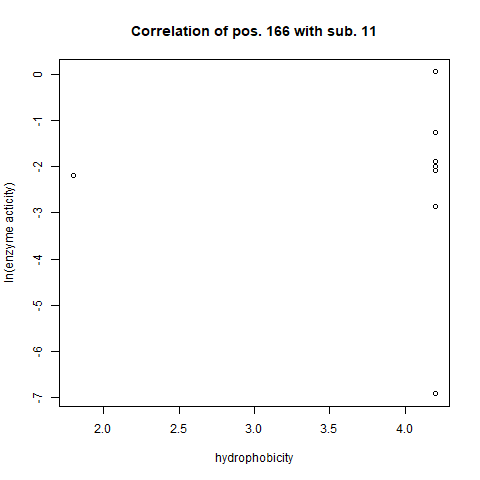

Supplement: Supplementary file 6 — Supplementary Data 3 [file 42004_2024_1207_MOESM6_ESM.zip › Supplementary Data 3/plots/hydrophobicity - 166 - 11 .png]

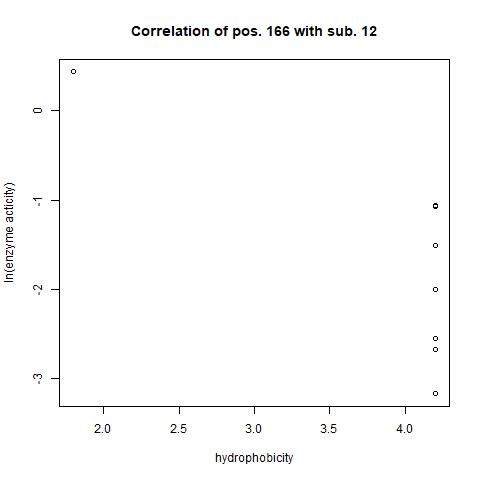

Supplement: Supplementary file 6 — Supplementary Data 3 [file 42004_2024_1207_MOESM6_ESM.zip › Supplementary Data 3/plots/hydrophobicity - 166 - 12 .png]

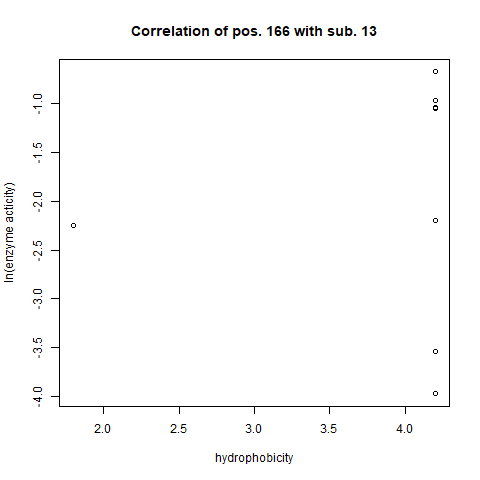

Supplement: Supplementary file 6 — Supplementary Data 3 [file 42004_2024_1207_MOESM6_ESM.zip › Supplementary Data 3/plots/hydrophobicity - 166 - 13 .png]

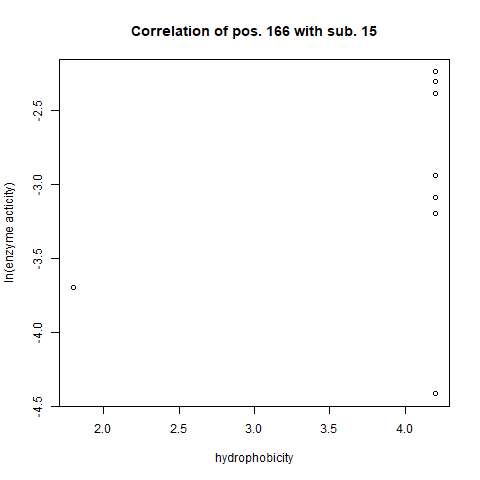

Supplement: Supplementary file 6 — Supplementary Data 3 [file 42004_2024_1207_MOESM6_ESM.zip › Supplementary Data 3/plots/hydrophobicity - 166 - 15 .png]

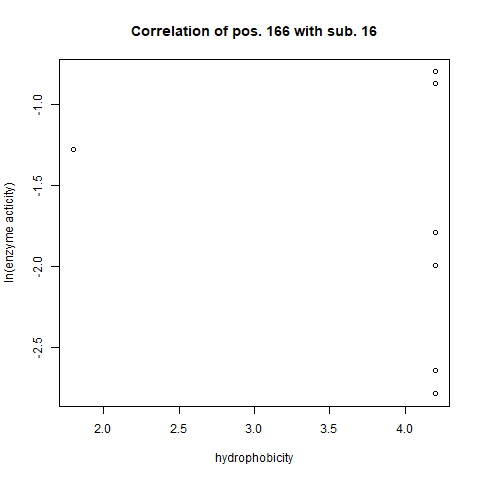

Supplement: Supplementary file 6 — Supplementary Data 3 [file 42004_2024_1207_MOESM6_ESM.zip › Supplementary Data 3/plots/hydrophobicity - 166 - 16 .png]

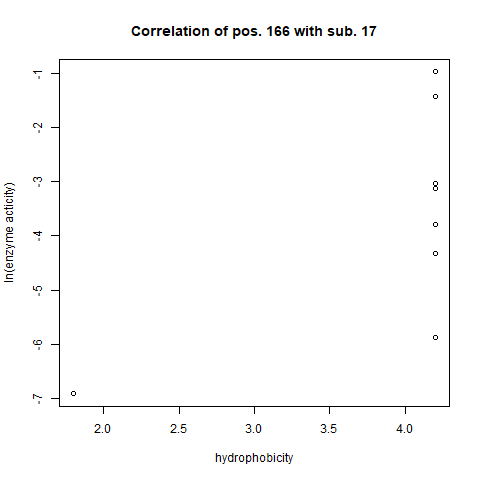

Supplement: Supplementary file 6 — Supplementary Data 3 [file 42004_2024_1207_MOESM6_ESM.zip › Supplementary Data 3/plots/hydrophobicity - 166 - 17 .png]

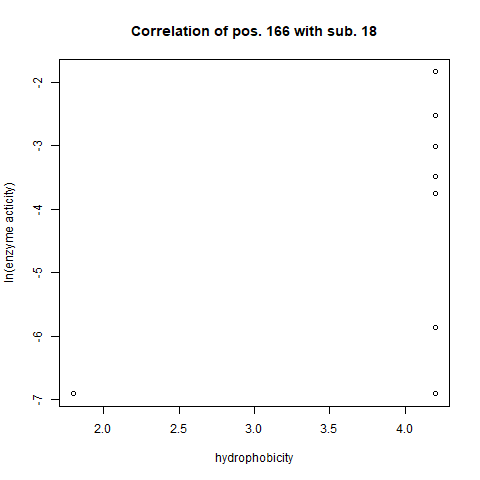

Supplement: Supplementary file 6 — Supplementary Data 3 [file 42004_2024_1207_MOESM6_ESM.zip › Supplementary Data 3/plots/hydrophobicity - 166 - 18 .png]

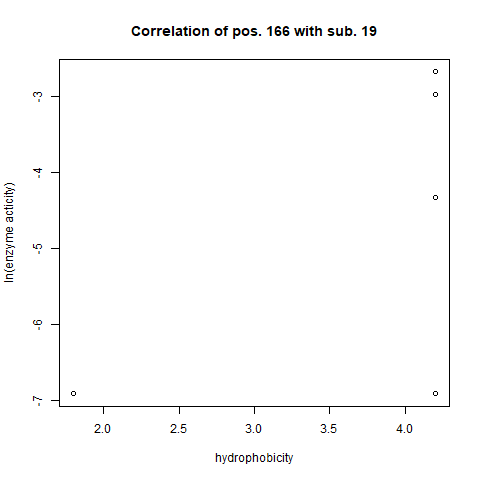

Supplement: Supplementary file 6 — Supplementary Data 3 [file 42004_2024_1207_MOESM6_ESM.zip › Supplementary Data 3/plots/hydrophobicity - 166 - 19 .png]

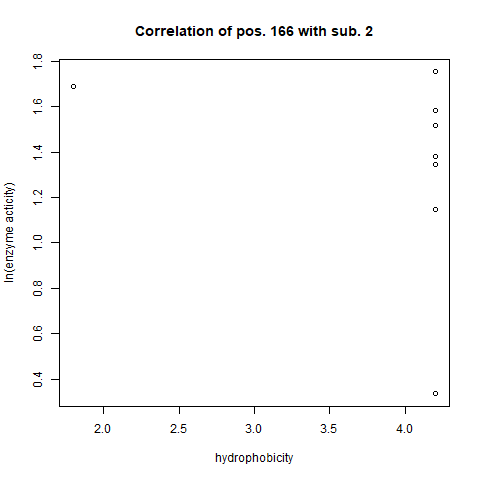

Supplement: Supplementary file 6 — Supplementary Data 3 [file 42004_2024_1207_MOESM6_ESM.zip › Supplementary Data 3/plots/hydrophobicity - 166 - 2 .png]

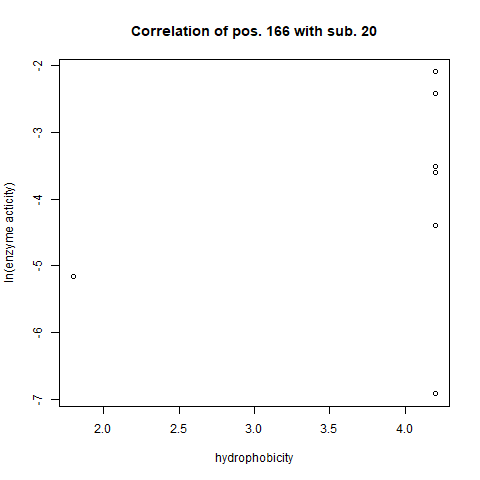

Supplement: Supplementary file 6 — Supplementary Data 3 [file 42004_2024_1207_MOESM6_ESM.zip › Supplementary Data 3/plots/hydrophobicity - 166 - 20 .png]

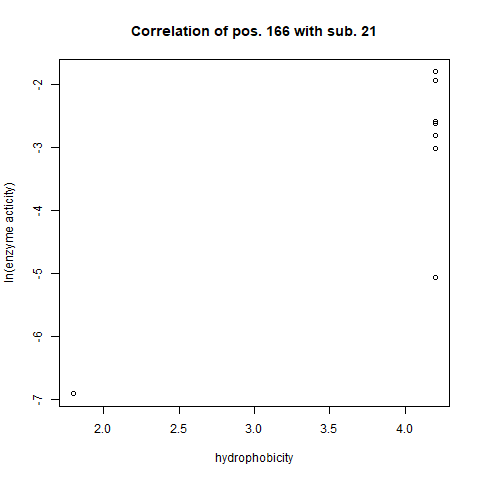

Supplement: Supplementary file 6 — Supplementary Data 3 [file 42004_2024_1207_MOESM6_ESM.zip › Supplementary Data 3/plots/hydrophobicity - 166 - 21 .png]

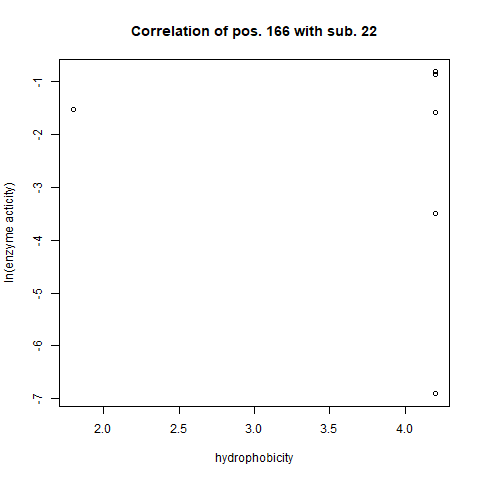

Supplement: Supplementary file 6 — Supplementary Data 3 [file 42004_2024_1207_MOESM6_ESM.zip › Supplementary Data 3/plots/hydrophobicity - 166 - 22 .png]

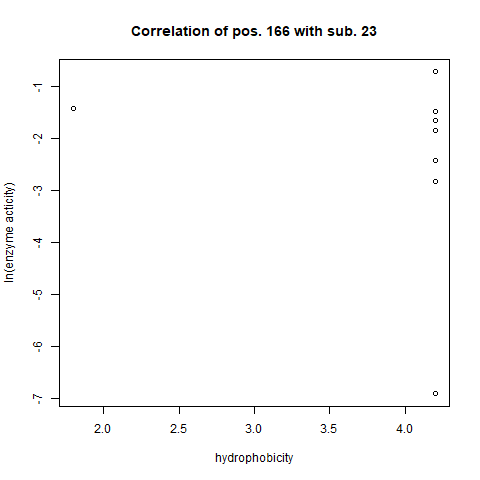

Supplement: Supplementary file 6 — Supplementary Data 3 [file 42004_2024_1207_MOESM6_ESM.zip › Supplementary Data 3/plots/hydrophobicity - 166 - 23 .png]

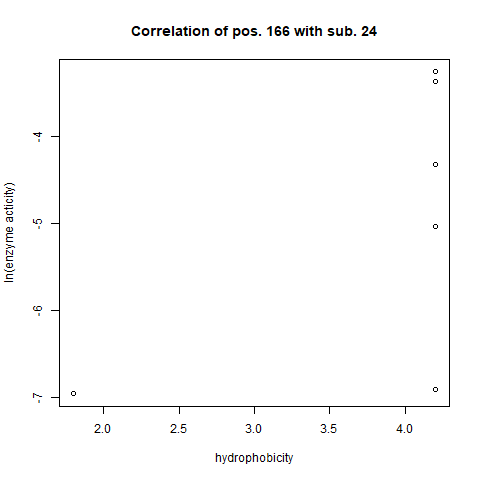

Supplement: Supplementary file 6 — Supplementary Data 3 [file 42004_2024_1207_MOESM6_ESM.zip › Supplementary Data 3/plots/hydrophobicity - 166 - 24 .png]

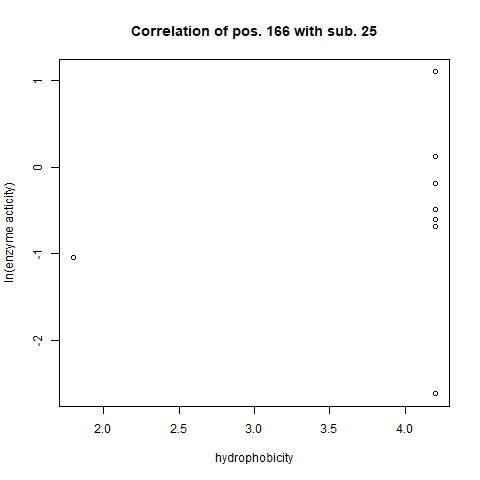

Supplement: Supplementary file 6 — Supplementary Data 3 [file 42004_2024_1207_MOESM6_ESM.zip › Supplementary Data 3/plots/hydrophobicity - 166 - 25 .png]

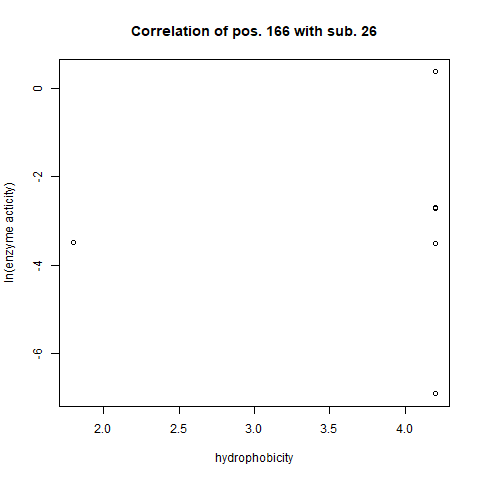

Supplement: Supplementary file 6 — Supplementary Data 3 [file 42004_2024_1207_MOESM6_ESM.zip › Supplementary Data 3/plots/hydrophobicity - 166 - 26 .png]

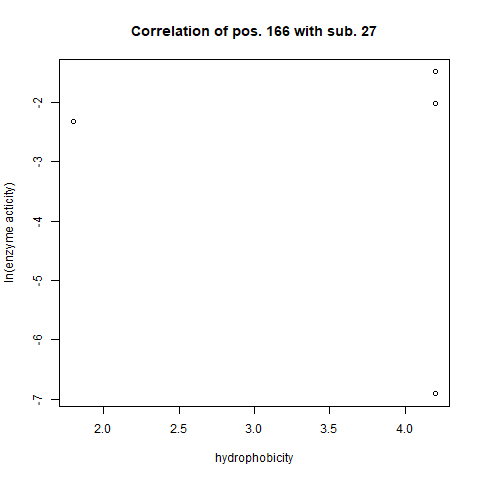

Supplement: Supplementary file 6 — Supplementary Data 3 [file 42004_2024_1207_MOESM6_ESM.zip › Supplementary Data 3/plots/hydrophobicity - 166 - 27 .png]

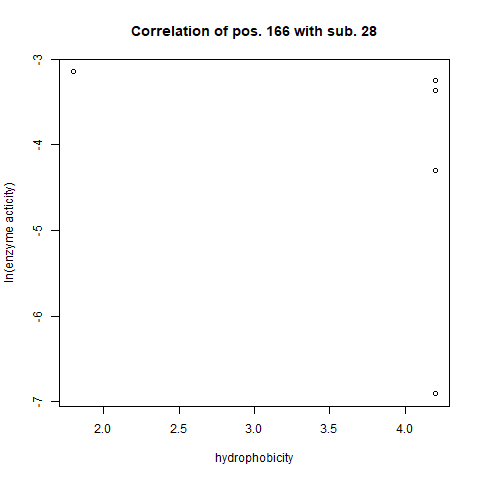

Supplement: Supplementary file 6 — Supplementary Data 3 [file 42004_2024_1207_MOESM6_ESM.zip › Supplementary Data 3/plots/hydrophobicity - 166 - 28 .png]

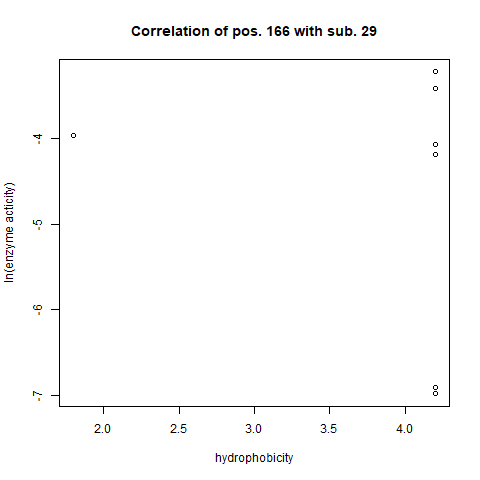

Supplement: Supplementary file 6 — Supplementary Data 3 [file 42004_2024_1207_MOESM6_ESM.zip › Supplementary Data 3/plots/hydrophobicity - 166 - 29 .png]

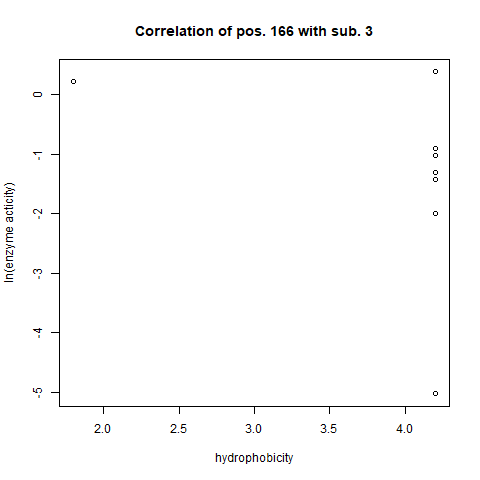

Supplement: Supplementary file 6 — Supplementary Data 3 [file 42004_2024_1207_MOESM6_ESM.zip › Supplementary Data 3/plots/hydrophobicity - 166 - 3 .png]

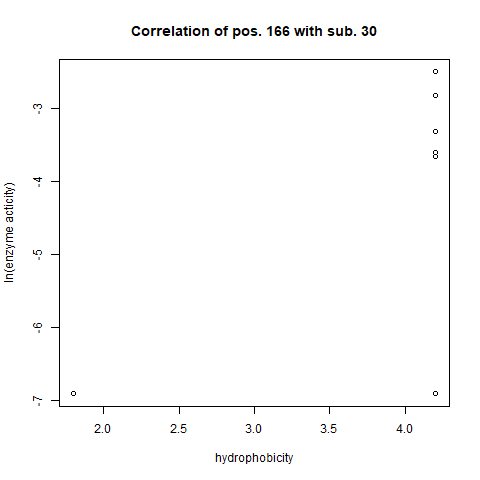

Supplement: Supplementary file 6 — Supplementary Data 3 [file 42004_2024_1207_MOESM6_ESM.zip › Supplementary Data 3/plots/hydrophobicity - 166 - 30 .png]

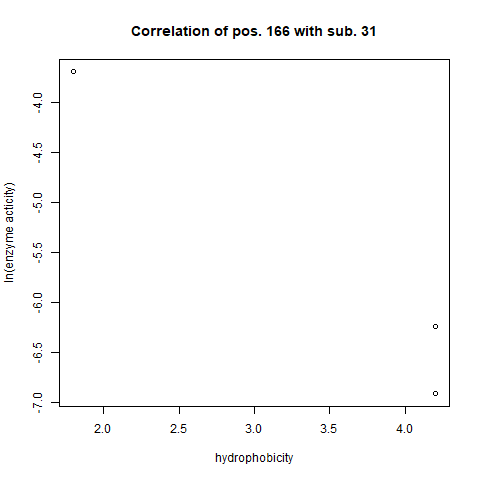

Supplement: Supplementary file 6 — Supplementary Data 3 [file 42004_2024_1207_MOESM6_ESM.zip › Supplementary Data 3/plots/hydrophobicity - 166 - 31 .png]

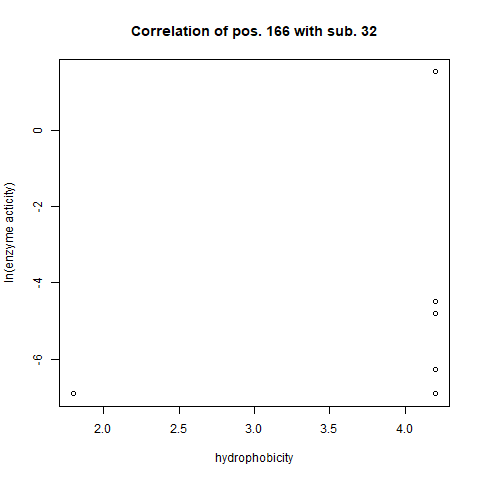

Supplement: Supplementary file 6 — Supplementary Data 3 [file 42004_2024_1207_MOESM6_ESM.zip › Supplementary Data 3/plots/hydrophobicity - 166 - 32 .png]

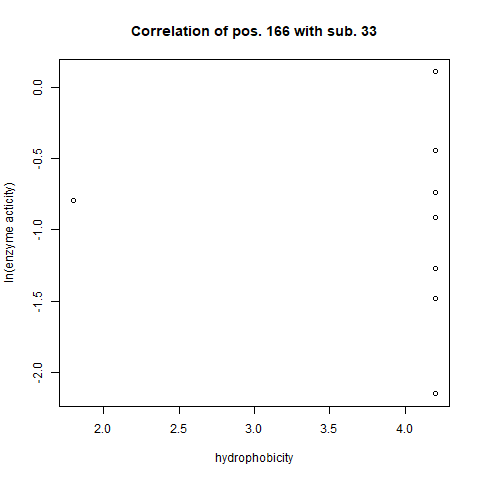

Supplement: Supplementary file 6 — Supplementary Data 3 [file 42004_2024_1207_MOESM6_ESM.zip › Supplementary Data 3/plots/hydrophobicity - 166 - 33 .png]

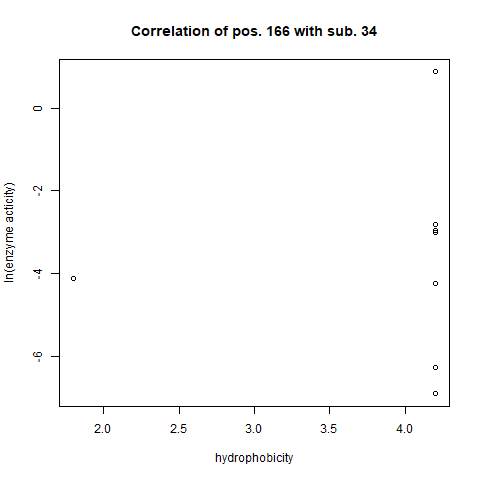

Supplement: Supplementary file 6 — Supplementary Data 3 [file 42004_2024_1207_MOESM6_ESM.zip › Supplementary Data 3/plots/hydrophobicity - 166 - 34 .png]

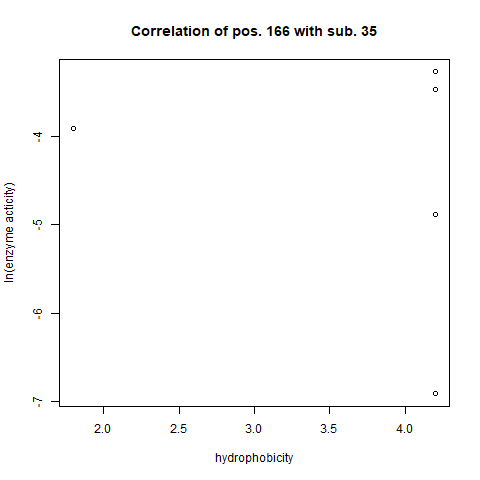

Supplement: Supplementary file 6 — Supplementary Data 3 [file 42004_2024_1207_MOESM6_ESM.zip › Supplementary Data 3/plots/hydrophobicity - 166 - 35 .png]

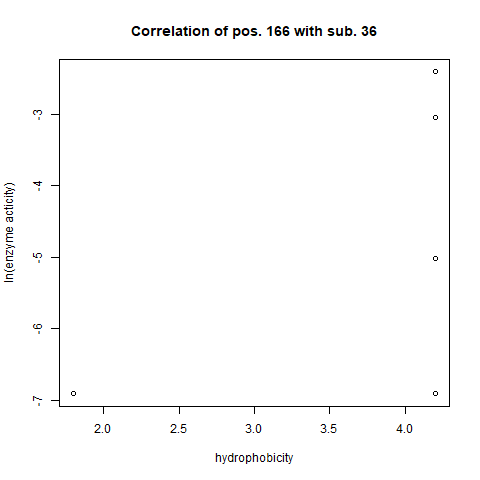

Supplement: Supplementary file 6 — Supplementary Data 3 [file 42004_2024_1207_MOESM6_ESM.zip › Supplementary Data 3/plots/hydrophobicity - 166 - 36 .png]

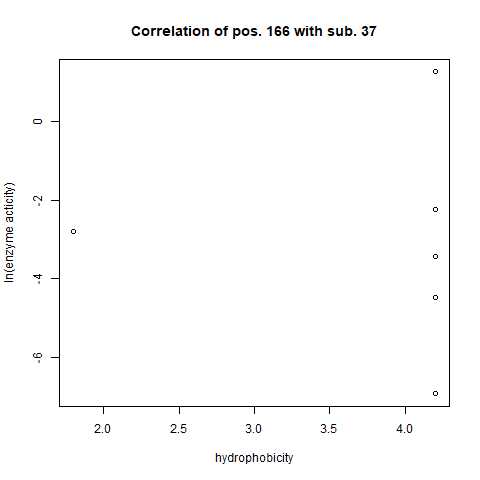

Supplement: Supplementary file 6 — Supplementary Data 3 [file 42004_2024_1207_MOESM6_ESM.zip › Supplementary Data 3/plots/hydrophobicity - 166 - 37 .png]

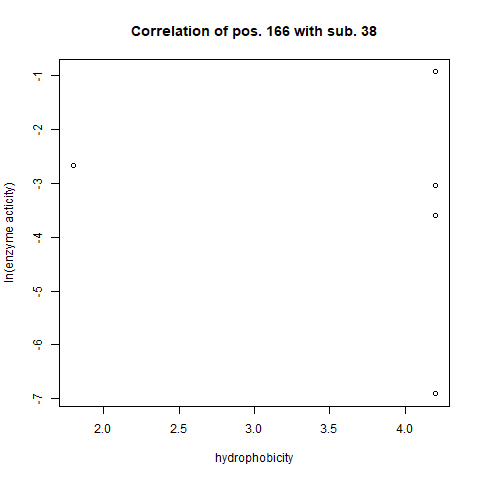

Supplement: Supplementary file 6 — Supplementary Data 3 [file 42004_2024_1207_MOESM6_ESM.zip › Supplementary Data 3/plots/hydrophobicity - 166 - 38 .png]

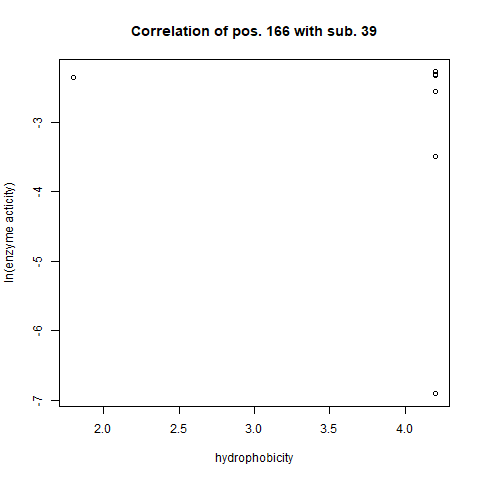

Supplement: Supplementary file 6 — Supplementary Data 3 [file 42004_2024_1207_MOESM6_ESM.zip › Supplementary Data 3/plots/hydrophobicity - 166 - 39 .png]

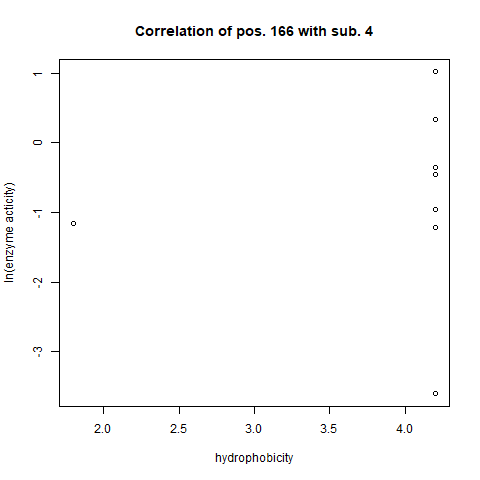

Supplement: Supplementary file 6 — Supplementary Data 3 [file 42004_2024_1207_MOESM6_ESM.zip › Supplementary Data 3/plots/hydrophobicity - 166 - 4 .png]

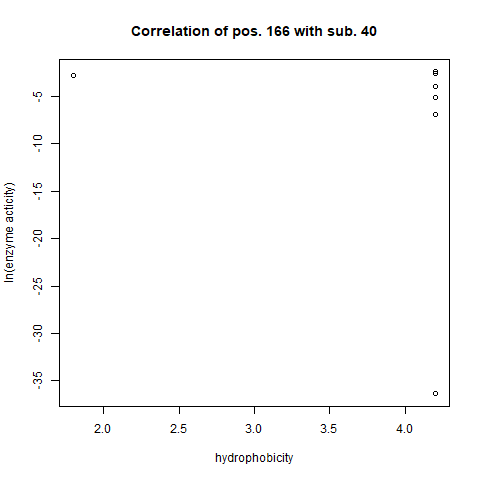

Supplement: Supplementary file 6 — Supplementary Data 3 [file 42004_2024_1207_MOESM6_ESM.zip › Supplementary Data 3/plots/hydrophobicity - 166 - 40 .png]

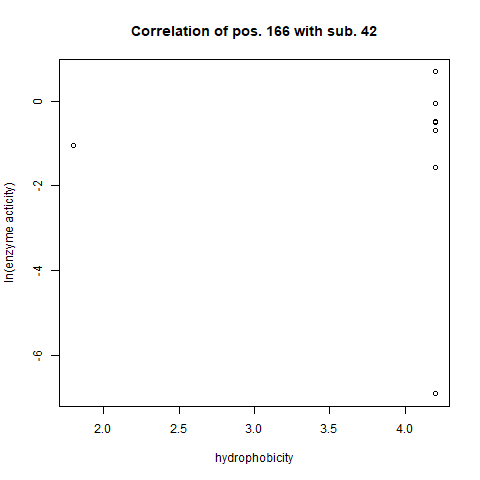

Supplement: Supplementary file 6 — Supplementary Data 3 [file 42004_2024_1207_MOESM6_ESM.zip › Supplementary Data 3/plots/hydrophobicity - 166 - 42 .png]

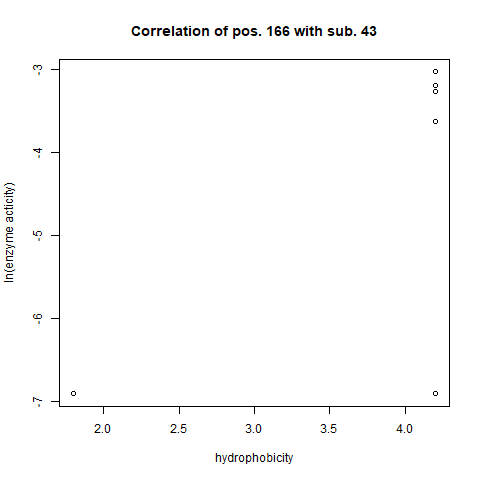

Supplement: Supplementary file 6 — Supplementary Data 3 [file 42004_2024_1207_MOESM6_ESM.zip › Supplementary Data 3/plots/hydrophobicity - 166 - 43 .png]

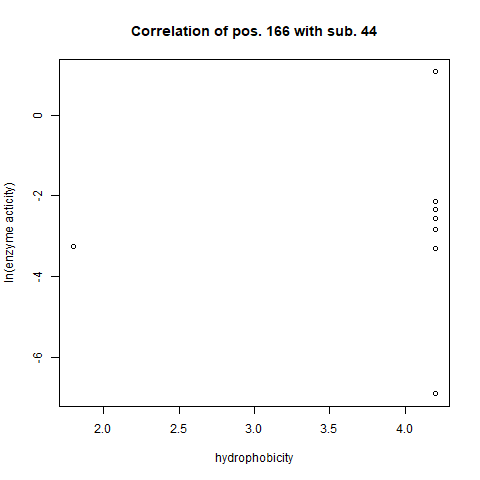

Supplement: Supplementary file 6 — Supplementary Data 3 [file 42004_2024_1207_MOESM6_ESM.zip › Supplementary Data 3/plots/hydrophobicity - 166 - 44 .png]

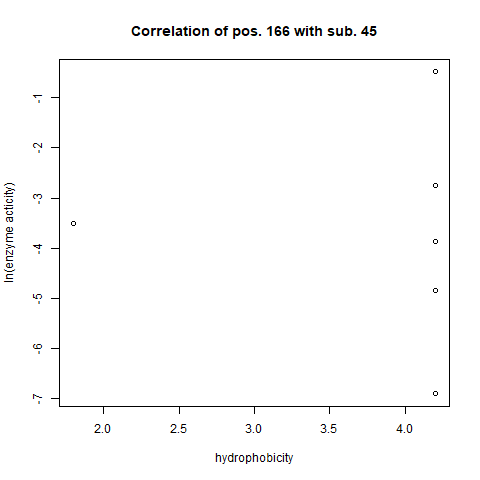

Supplement: Supplementary file 6 — Supplementary Data 3 [file 42004_2024_1207_MOESM6_ESM.zip › Supplementary Data 3/plots/hydrophobicity - 166 - 45 .png]

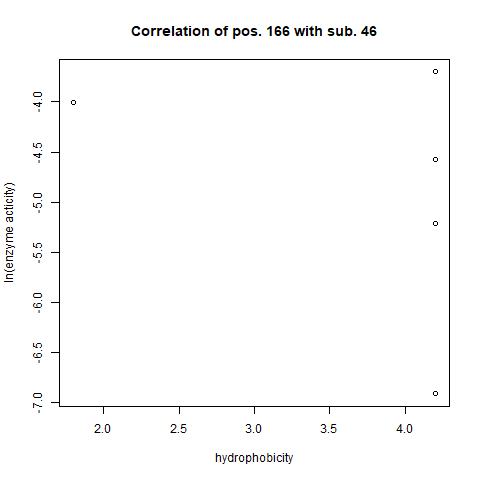

Supplement: Supplementary file 6 — Supplementary Data 3 [file 42004_2024_1207_MOESM6_ESM.zip › Supplementary Data 3/plots/hydrophobicity - 166 - 46 .png]

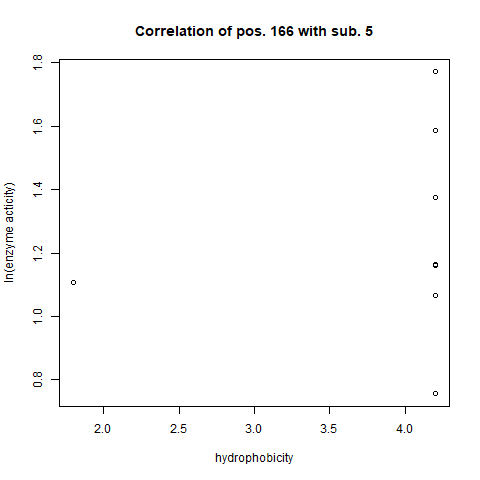

Supplement: Supplementary file 6 — Supplementary Data 3 [file 42004_2024_1207_MOESM6_ESM.zip › Supplementary Data 3/plots/hydrophobicity - 166 - 5 .png]

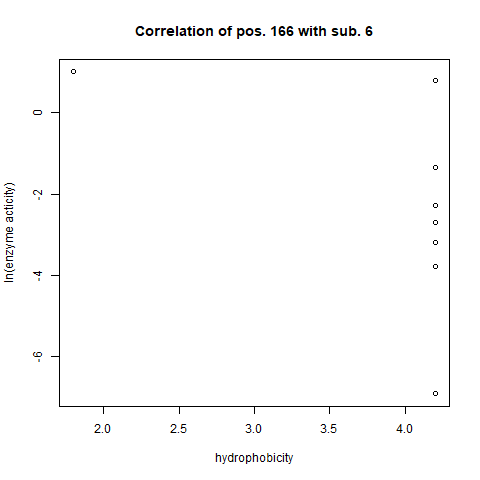

Supplement: Supplementary file 6 — Supplementary Data 3 [file 42004_2024_1207_MOESM6_ESM.zip › Supplementary Data 3/plots/hydrophobicity - 166 - 6 .png]

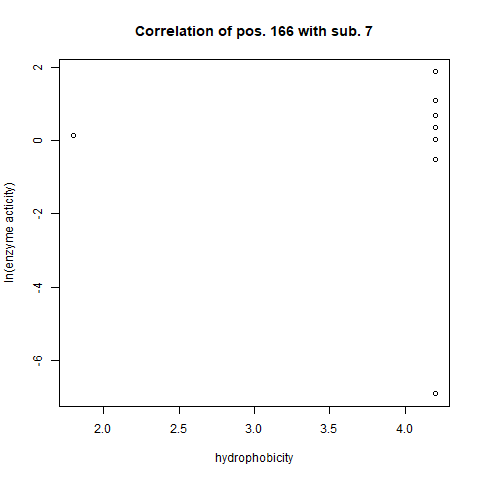

Supplement: Supplementary file 6 — Supplementary Data 3 [file 42004_2024_1207_MOESM6_ESM.zip › Supplementary Data 3/plots/hydrophobicity - 166 - 7 .png]

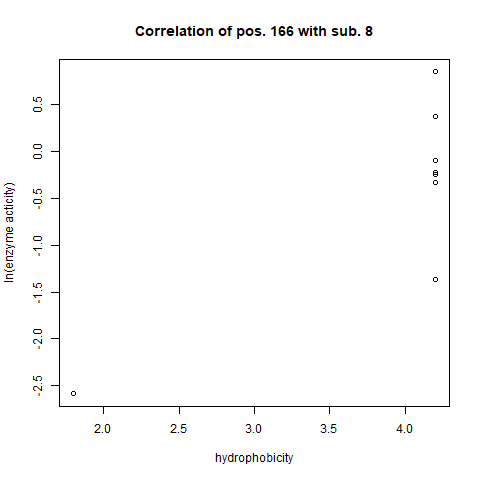

Supplement: Supplementary file 6 — Supplementary Data 3 [file 42004_2024_1207_MOESM6_ESM.zip › Supplementary Data 3/plots/hydrophobicity - 166 - 8 .png]

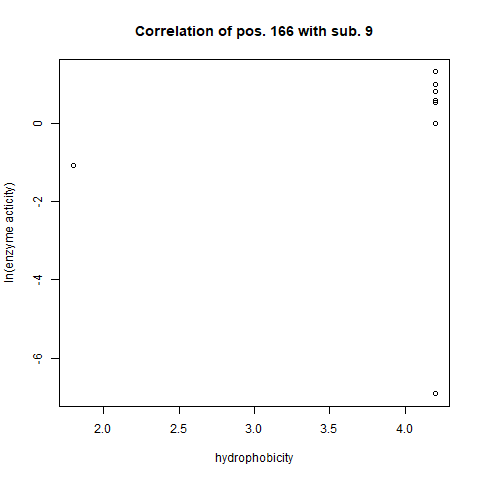

Supplement: Supplementary file 6 — Supplementary Data 3 [file 42004_2024_1207_MOESM6_ESM.zip › Supplementary Data 3/plots/hydrophobicity - 166 - 9 .png]

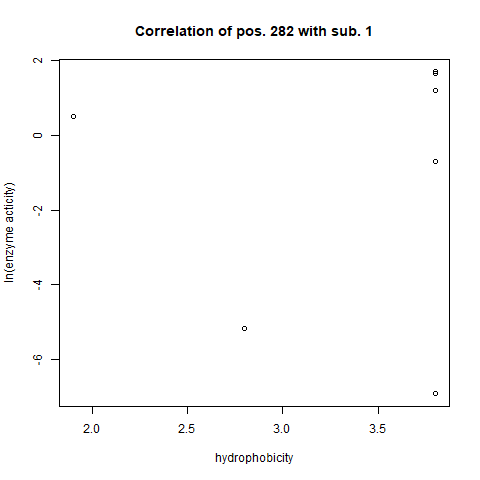

Supplement: Supplementary file 6 — Supplementary Data 3 [file 42004_2024_1207_MOESM6_ESM.zip › Supplementary Data 3/plots/hydrophobicity - 282 - 1 .png]

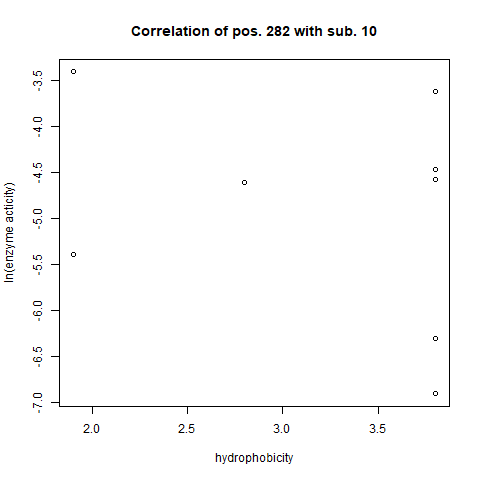

Supplement: Supplementary file 6 — Supplementary Data 3 [file 42004_2024_1207_MOESM6_ESM.zip › Supplementary Data 3/plots/hydrophobicity - 282 - 10 .png]

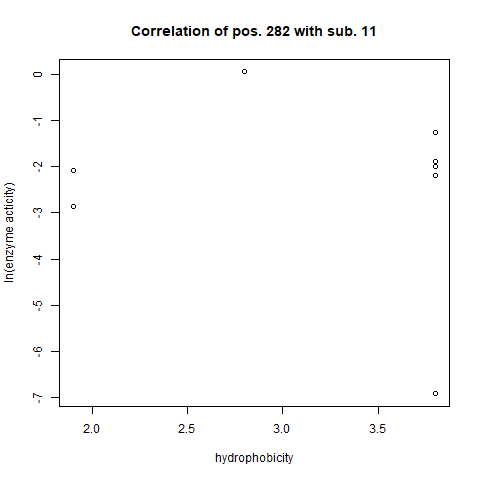

Supplement: Supplementary file 6 — Supplementary Data 3 [file 42004_2024_1207_MOESM6_ESM.zip › Supplementary Data 3/plots/hydrophobicity - 282 - 11 .png]

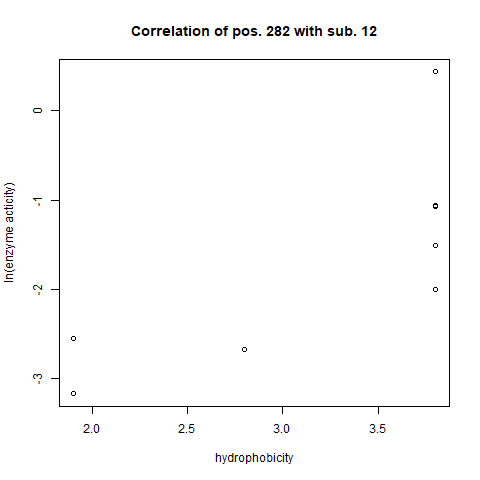

Supplement: Supplementary file 6 — Supplementary Data 3 [file 42004_2024_1207_MOESM6_ESM.zip › Supplementary Data 3/plots/hydrophobicity - 282 - 12 .png]

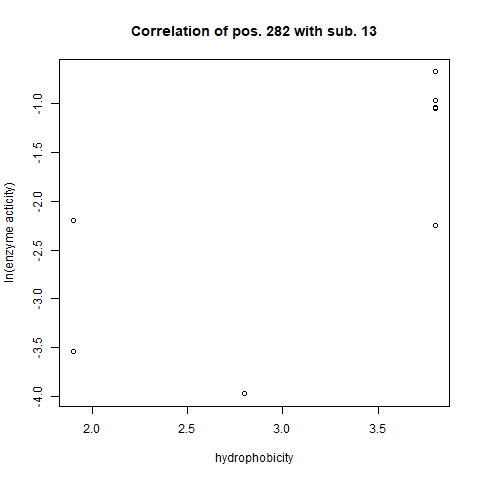

Supplement: Supplementary file 6 — Supplementary Data 3 [file 42004_2024_1207_MOESM6_ESM.zip › Supplementary Data 3/plots/hydrophobicity - 282 - 13 .png]

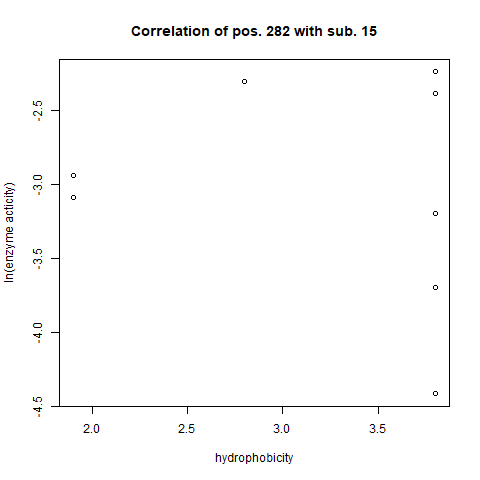

Supplement: Supplementary file 6 — Supplementary Data 3 [file 42004_2024_1207_MOESM6_ESM.zip › Supplementary Data 3/plots/hydrophobicity - 282 - 15 .png]

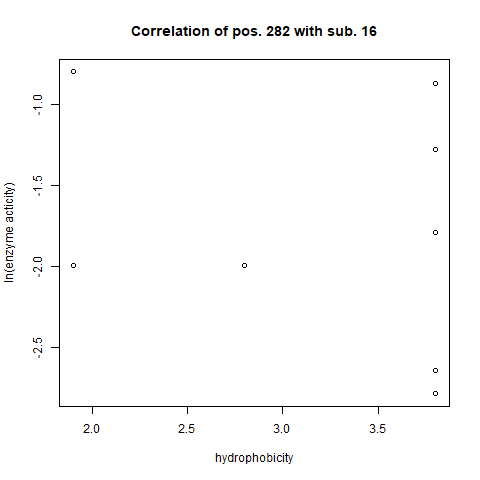

Supplement: Supplementary file 6 — Supplementary Data 3 [file 42004_2024_1207_MOESM6_ESM.zip › Supplementary Data 3/plots/hydrophobicity - 282 - 16 .png]

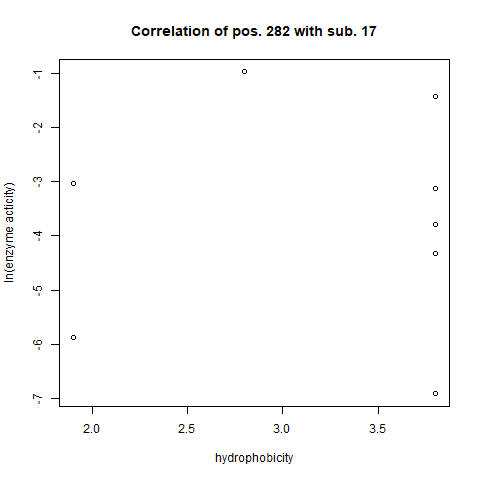

Supplement: Supplementary file 6 — Supplementary Data 3 [file 42004_2024_1207_MOESM6_ESM.zip › Supplementary Data 3/plots/hydrophobicity - 282 - 17 .png]

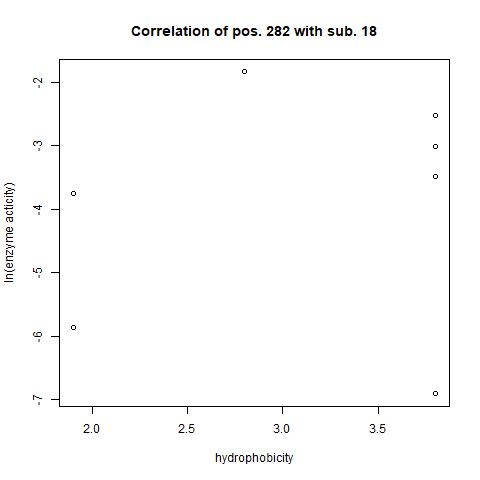

Supplement: Supplementary file 6 — Supplementary Data 3 [file 42004_2024_1207_MOESM6_ESM.zip › Supplementary Data 3/plots/hydrophobicity - 282 - 18 .png]

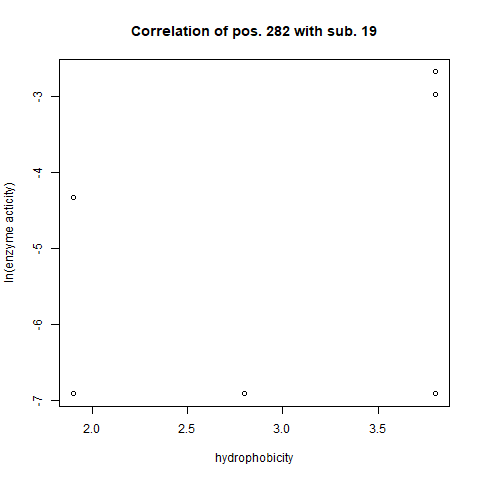

Supplement: Supplementary file 6 — Supplementary Data 3 [file 42004_2024_1207_MOESM6_ESM.zip › Supplementary Data 3/plots/hydrophobicity - 282 - 19 .png]

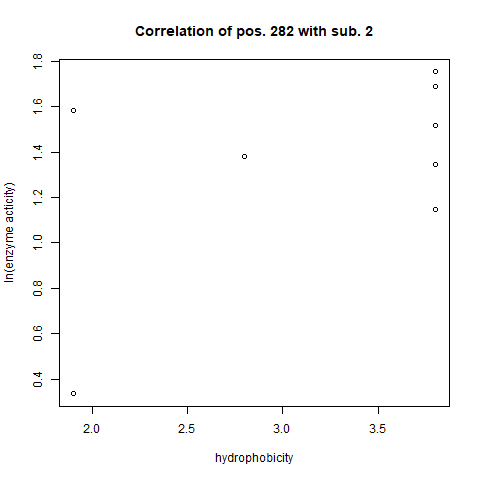

Supplement: Supplementary file 6 — Supplementary Data 3 [file 42004_2024_1207_MOESM6_ESM.zip › Supplementary Data 3/plots/hydrophobicity - 282 - 2 .png]

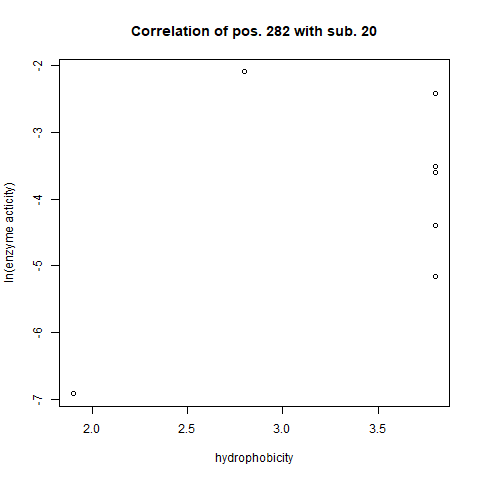

Supplement: Supplementary file 6 — Supplementary Data 3 [file 42004_2024_1207_MOESM6_ESM.zip › Supplementary Data 3/plots/hydrophobicity - 282 - 20 .png]

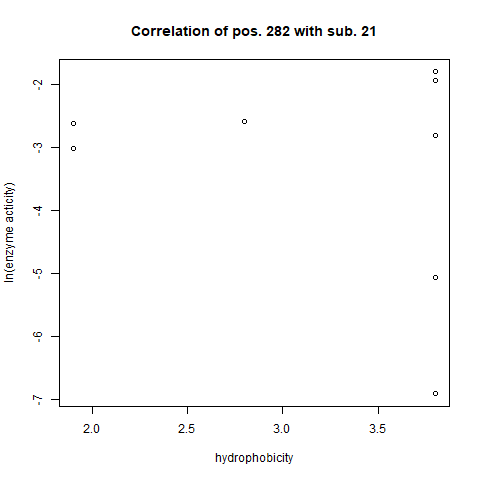

Supplement: Supplementary file 6 — Supplementary Data 3 [file 42004_2024_1207_MOESM6_ESM.zip › Supplementary Data 3/plots/hydrophobicity - 282 - 21 .png]

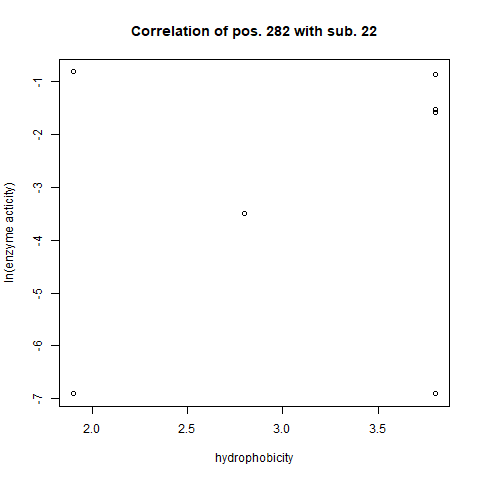

Supplement: Supplementary file 6 — Supplementary Data 3 [file 42004_2024_1207_MOESM6_ESM.zip › Supplementary Data 3/plots/hydrophobicity - 282 - 22 .png]

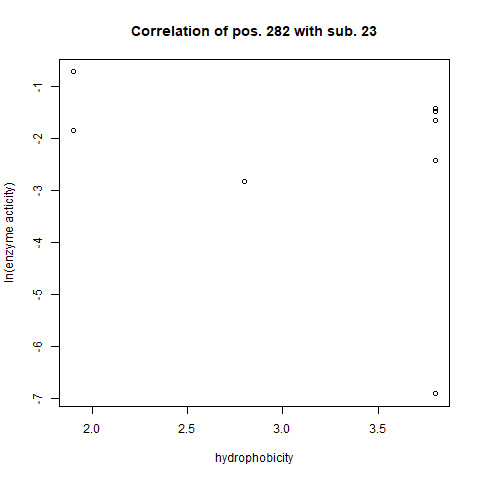

Supplement: Supplementary file 6 — Supplementary Data 3 [file 42004_2024_1207_MOESM6_ESM.zip › Supplementary Data 3/plots/hydrophobicity - 282 - 23 .png]

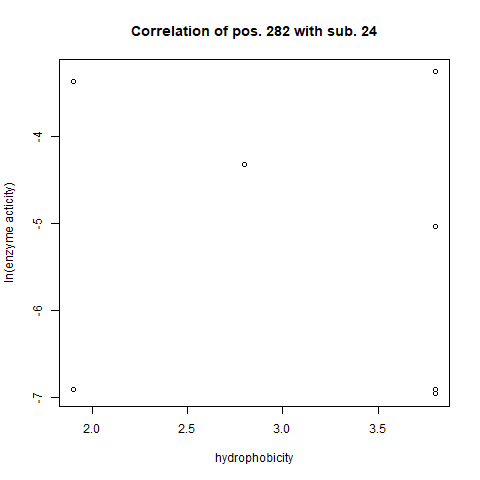

Supplement: Supplementary file 6 — Supplementary Data 3 [file 42004_2024_1207_MOESM6_ESM.zip › Supplementary Data 3/plots/hydrophobicity - 282 - 24 .png]

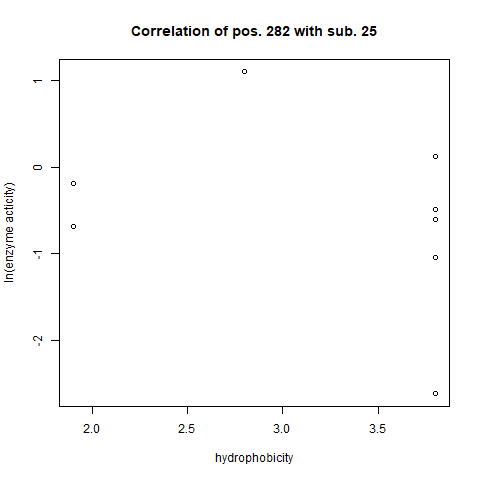

Supplement: Supplementary file 6 — Supplementary Data 3 [file 42004_2024_1207_MOESM6_ESM.zip › Supplementary Data 3/plots/hydrophobicity - 282 - 25 .png]

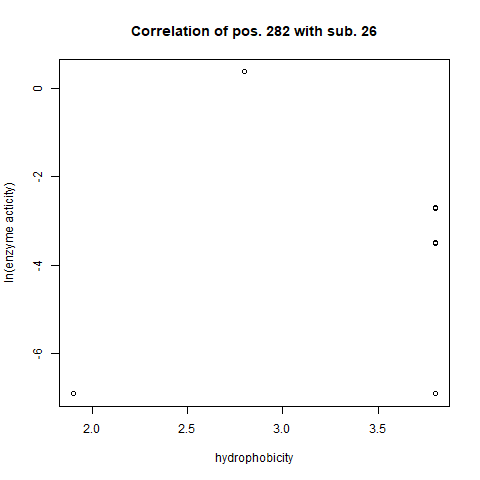

Supplement: Supplementary file 6 — Supplementary Data 3 [file 42004_2024_1207_MOESM6_ESM.zip › Supplementary Data 3/plots/hydrophobicity - 282 - 26 .png]

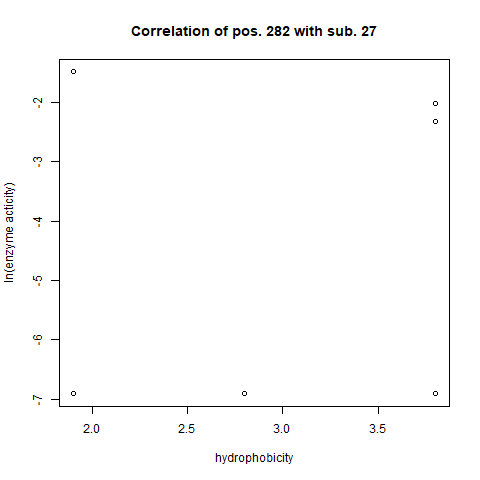

Supplement: Supplementary file 6 — Supplementary Data 3 [file 42004_2024_1207_MOESM6_ESM.zip › Supplementary Data 3/plots/hydrophobicity - 282 - 27 .png]

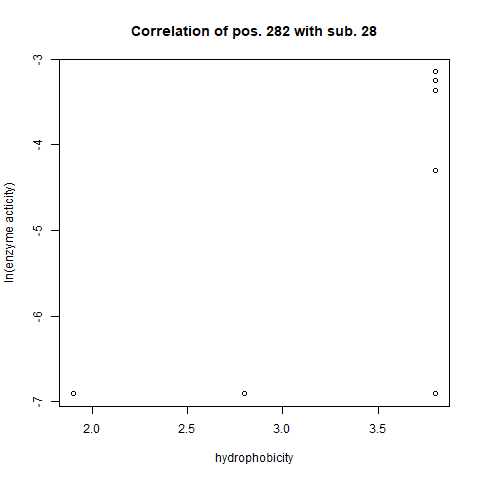

Supplement: Supplementary file 6 — Supplementary Data 3 [file 42004_2024_1207_MOESM6_ESM.zip › Supplementary Data 3/plots/hydrophobicity - 282 - 28 .png]

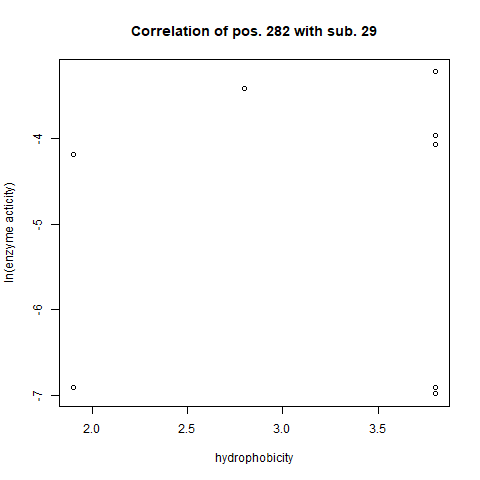

Supplement: Supplementary file 6 — Supplementary Data 3 [file 42004_2024_1207_MOESM6_ESM.zip › Supplementary Data 3/plots/hydrophobicity - 282 - 29 .png]

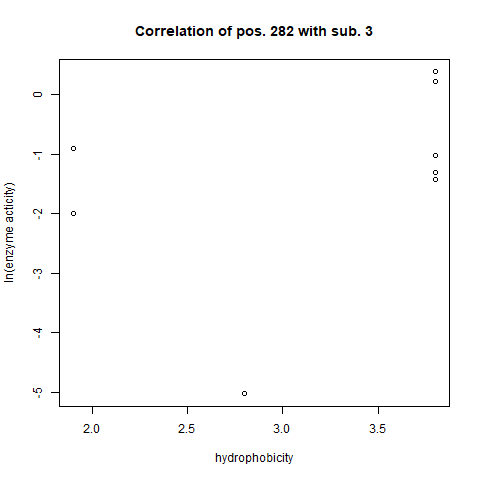

Supplement: Supplementary file 6 — Supplementary Data 3 [file 42004_2024_1207_MOESM6_ESM.zip › Supplementary Data 3/plots/hydrophobicity - 282 - 3 .png]

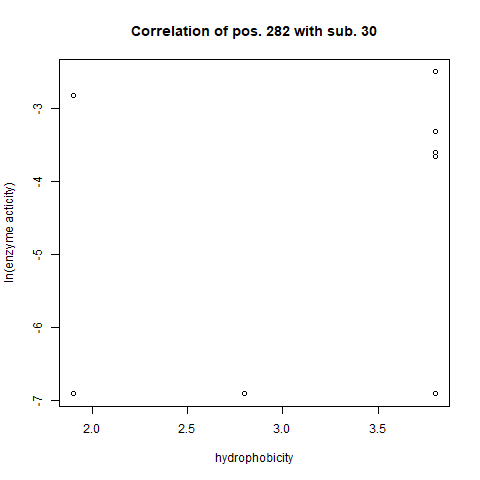

Supplement: Supplementary file 6 — Supplementary Data 3 [file 42004_2024_1207_MOESM6_ESM.zip › Supplementary Data 3/plots/hydrophobicity - 282 - 30 .png]

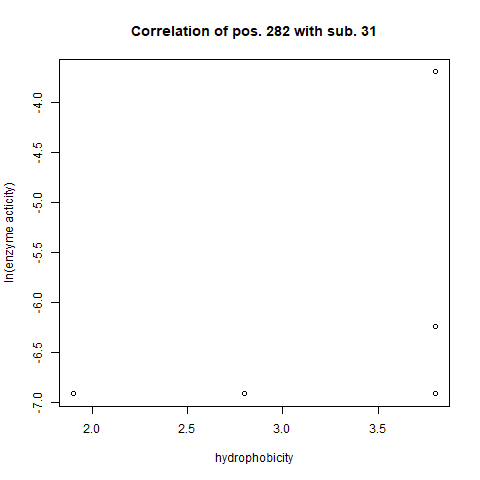

Supplement: Supplementary file 6 — Supplementary Data 3 [file 42004_2024_1207_MOESM6_ESM.zip › Supplementary Data 3/plots/hydrophobicity - 282 - 31 .png]

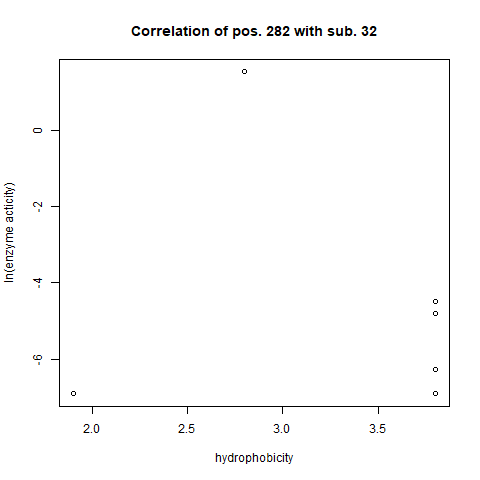

Supplement: Supplementary file 6 — Supplementary Data 3 [file 42004_2024_1207_MOESM6_ESM.zip › Supplementary Data 3/plots/hydrophobicity - 282 - 32 .png]

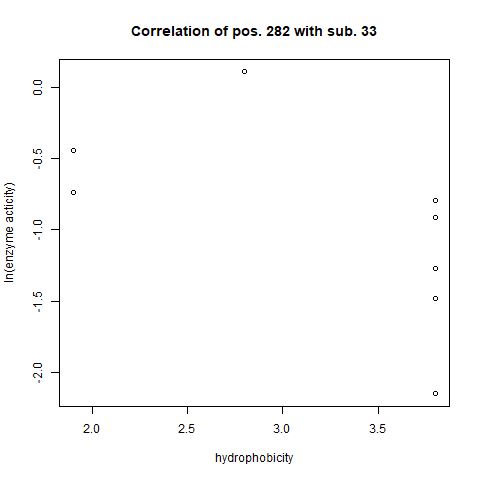

Supplement: Supplementary file 6 — Supplementary Data 3 [file 42004_2024_1207_MOESM6_ESM.zip › Supplementary Data 3/plots/hydrophobicity - 282 - 33 .png]

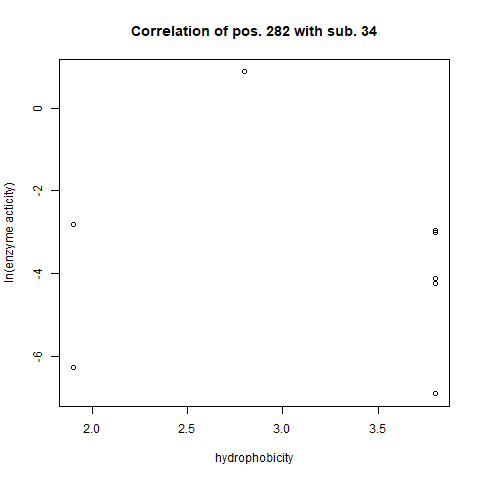

Supplement: Supplementary file 6 — Supplementary Data 3 [file 42004_2024_1207_MOESM6_ESM.zip › Supplementary Data 3/plots/hydrophobicity - 282 - 34 .png]

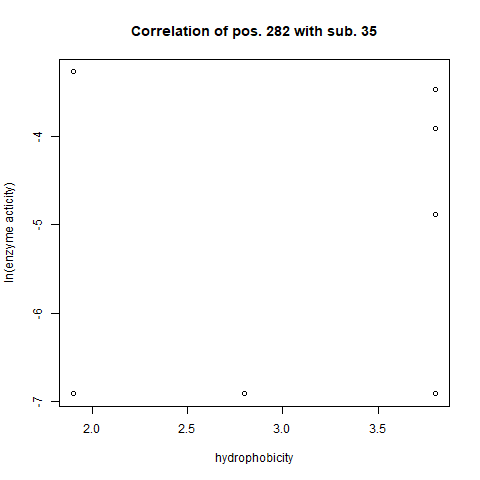

Supplement: Supplementary file 6 — Supplementary Data 3 [file 42004_2024_1207_MOESM6_ESM.zip › Supplementary Data 3/plots/hydrophobicity - 282 - 35 .png]

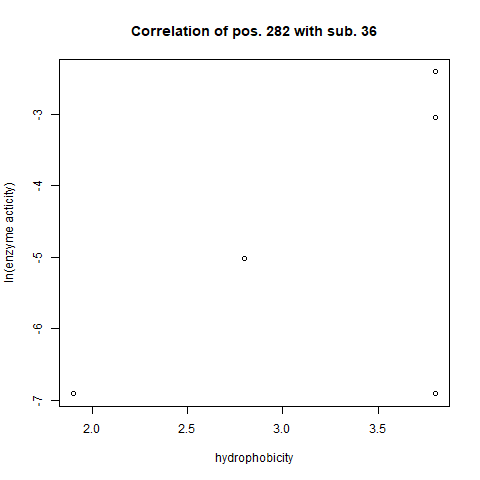

Supplement: Supplementary file 6 — Supplementary Data 3 [file 42004_2024_1207_MOESM6_ESM.zip › Supplementary Data 3/plots/hydrophobicity - 282 - 36 .png]

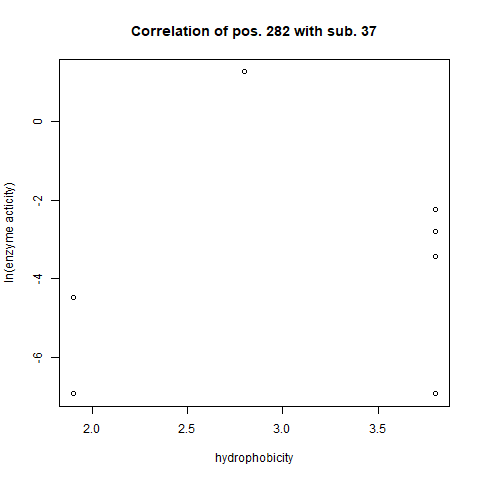

Supplement: Supplementary file 6 — Supplementary Data 3 [file 42004_2024_1207_MOESM6_ESM.zip › Supplementary Data 3/plots/hydrophobicity - 282 - 37 .png]

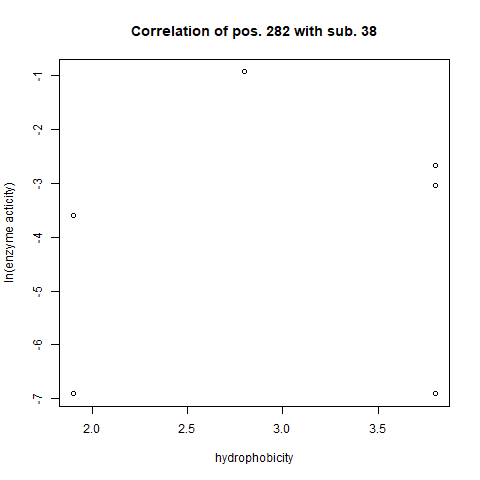

Supplement: Supplementary file 6 — Supplementary Data 3 [file 42004_2024_1207_MOESM6_ESM.zip › Supplementary Data 3/plots/hydrophobicity - 282 - 38 .png]

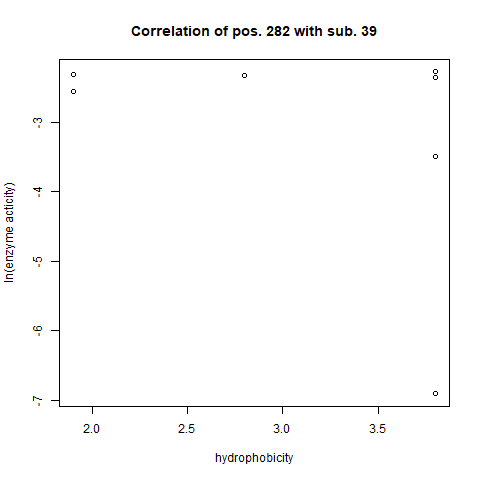

Supplement: Supplementary file 6 — Supplementary Data 3 [file 42004_2024_1207_MOESM6_ESM.zip › Supplementary Data 3/plots/hydrophobicity - 282 - 39 .png]

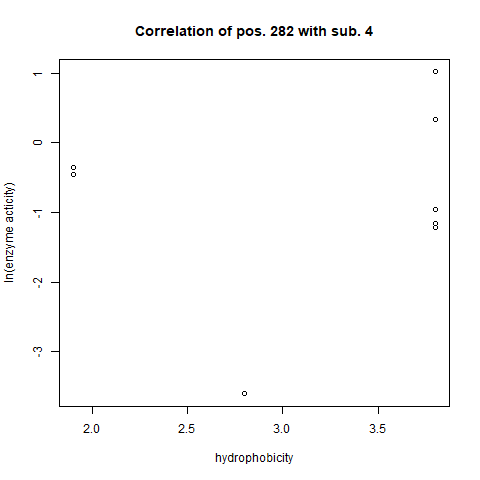

Supplement: Supplementary file 6 — Supplementary Data 3 [file 42004_2024_1207_MOESM6_ESM.zip › Supplementary Data 3/plots/hydrophobicity - 282 - 4 .png]

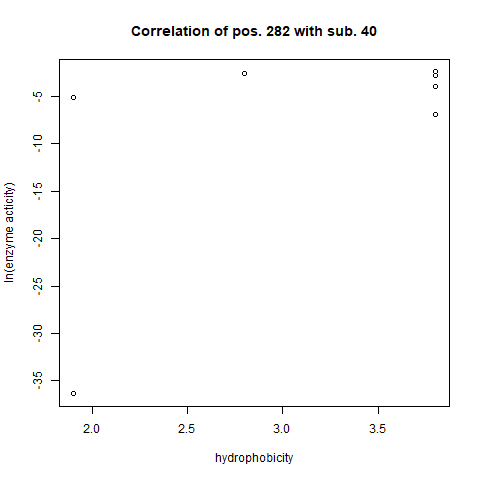

Supplement: Supplementary file 6 — Supplementary Data 3 [file 42004_2024_1207_MOESM6_ESM.zip › Supplementary Data 3/plots/hydrophobicity - 282 - 40 .png]

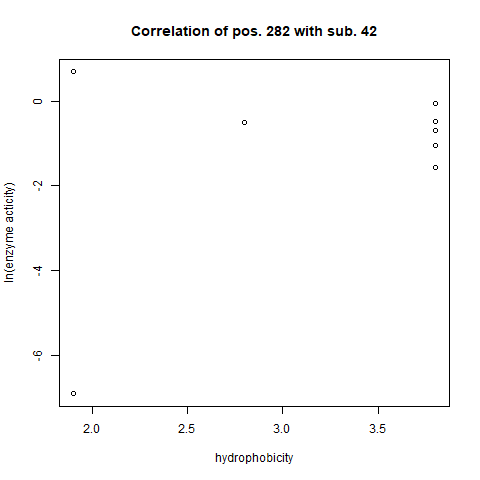

Supplement: Supplementary file 6 — Supplementary Data 3 [file 42004_2024_1207_MOESM6_ESM.zip › Supplementary Data 3/plots/hydrophobicity - 282 - 42 .png]

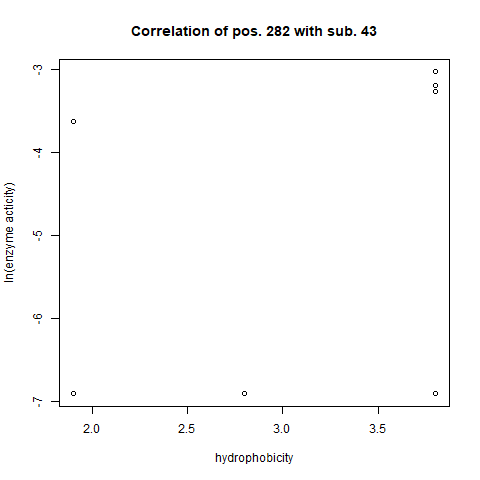

Supplement: Supplementary file 6 — Supplementary Data 3 [file 42004_2024_1207_MOESM6_ESM.zip › Supplementary Data 3/plots/hydrophobicity - 282 - 43 .png]

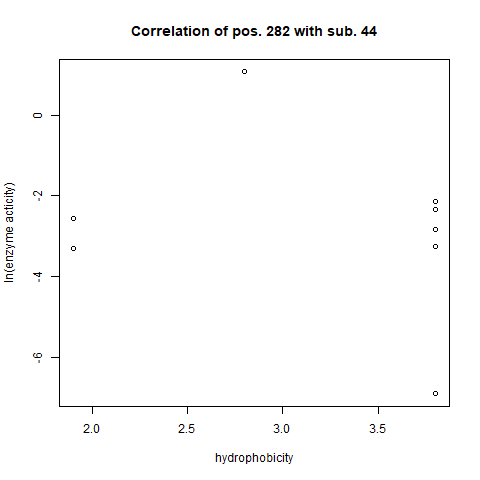

Supplement: Supplementary file 6 — Supplementary Data 3 [file 42004_2024_1207_MOESM6_ESM.zip › Supplementary Data 3/plots/hydrophobicity - 282 - 44 .png]

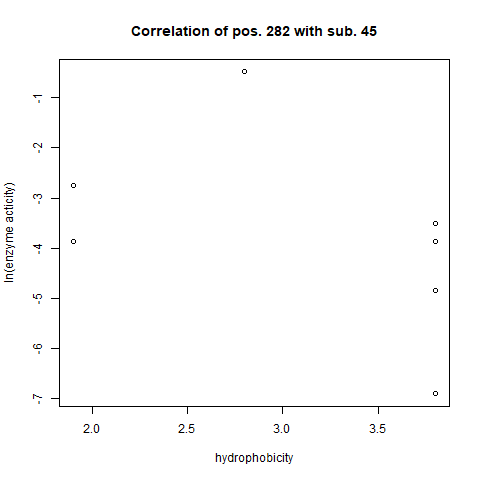

Supplement: Supplementary file 6 — Supplementary Data 3 [file 42004_2024_1207_MOESM6_ESM.zip › Supplementary Data 3/plots/hydrophobicity - 282 - 45 .png]

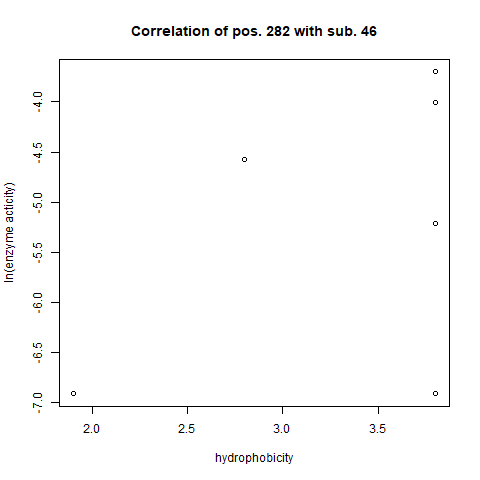

Supplement: Supplementary file 6 — Supplementary Data 3 [file 42004_2024_1207_MOESM6_ESM.zip › Supplementary Data 3/plots/hydrophobicity - 282 - 46 .png]

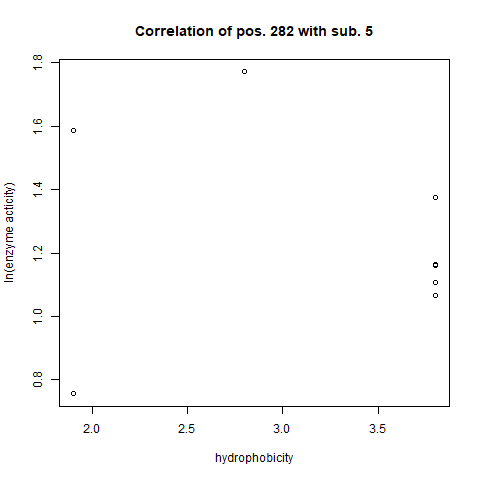

Supplement: Supplementary file 6 — Supplementary Data 3 [file 42004_2024_1207_MOESM6_ESM.zip › Supplementary Data 3/plots/hydrophobicity - 282 - 5 .png]

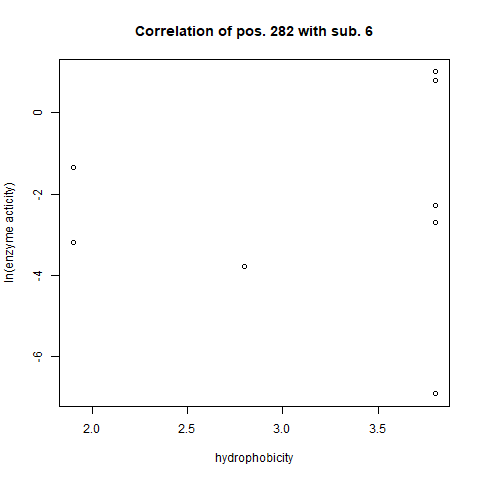

Supplement: Supplementary file 6 — Supplementary Data 3 [file 42004_2024_1207_MOESM6_ESM.zip › Supplementary Data 3/plots/hydrophobicity - 282 - 6 .png]

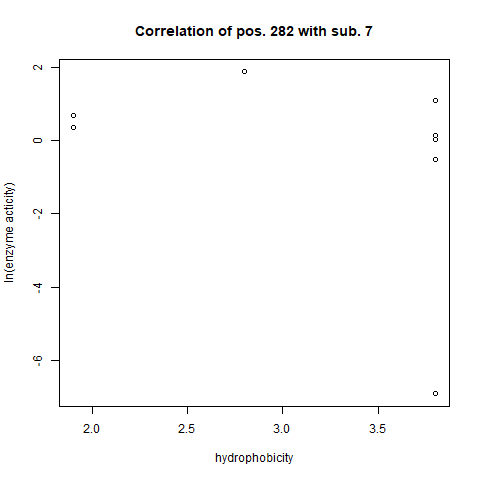

Supplement: Supplementary file 6 — Supplementary Data 3 [file 42004_2024_1207_MOESM6_ESM.zip › Supplementary Data 3/plots/hydrophobicity - 282 - 7 .png]

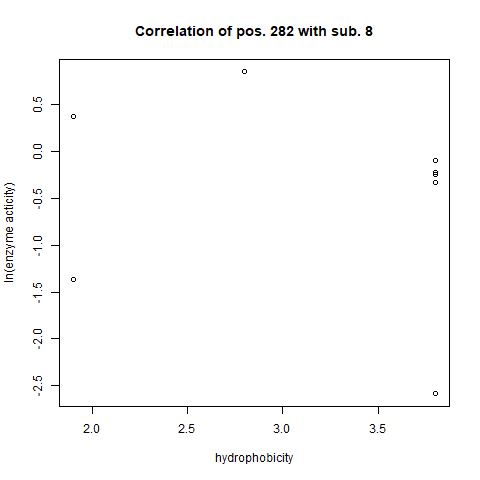

Supplement: Supplementary file 6 — Supplementary Data 3 [file 42004_2024_1207_MOESM6_ESM.zip › Supplementary Data 3/plots/hydrophobicity - 282 - 8 .png]

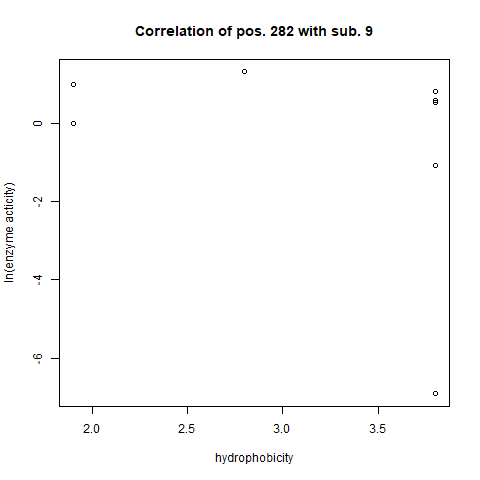

Supplement: Supplementary file 6 — Supplementary Data 3 [file 42004_2024_1207_MOESM6_ESM.zip › Supplementary Data 3/plots/hydrophobicity - 282 - 9 .png]

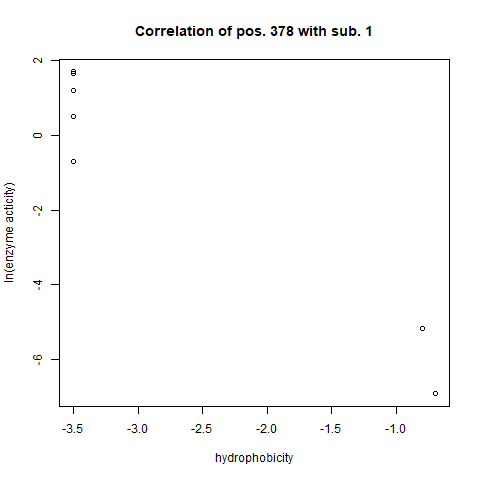

Supplement: Supplementary file 6 — Supplementary Data 3 [file 42004_2024_1207_MOESM6_ESM.zip › Supplementary Data 3/plots/hydrophobicity - 378 - 1 .png]

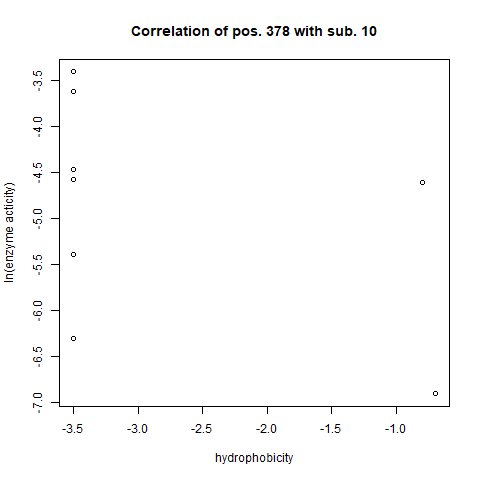

Supplement: Supplementary file 6 — Supplementary Data 3 [file 42004_2024_1207_MOESM6_ESM.zip › Supplementary Data 3/plots/hydrophobicity - 378 - 10 .png]

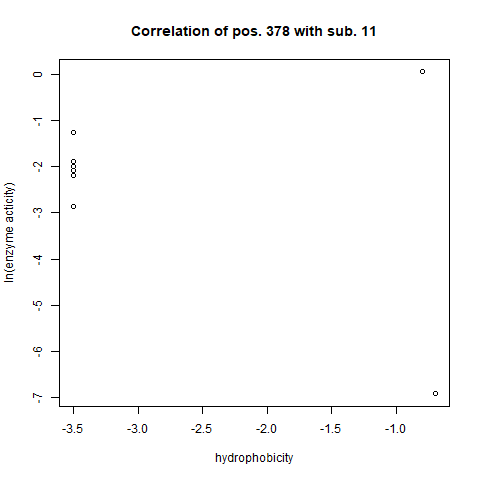

Supplement: Supplementary file 6 — Supplementary Data 3 [file 42004_2024_1207_MOESM6_ESM.zip › Supplementary Data 3/plots/hydrophobicity - 378 - 11 .png]

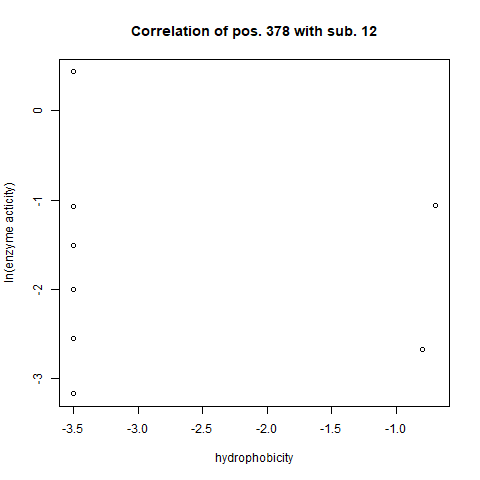

Supplement: Supplementary file 6 — Supplementary Data 3 [file 42004_2024_1207_MOESM6_ESM.zip › Supplementary Data 3/plots/hydrophobicity - 378 - 12 .png]

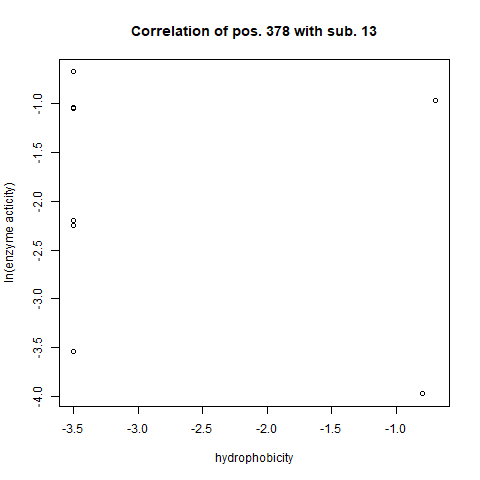

Supplement: Supplementary file 6 — Supplementary Data 3 [file 42004_2024_1207_MOESM6_ESM.zip › Supplementary Data 3/plots/hydrophobicity - 378 - 13 .png]

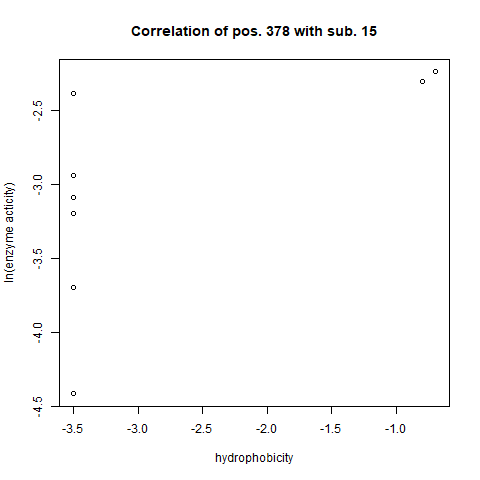

Supplement: Supplementary file 6 — Supplementary Data 3 [file 42004_2024_1207_MOESM6_ESM.zip › Supplementary Data 3/plots/hydrophobicity - 378 - 15 .png]

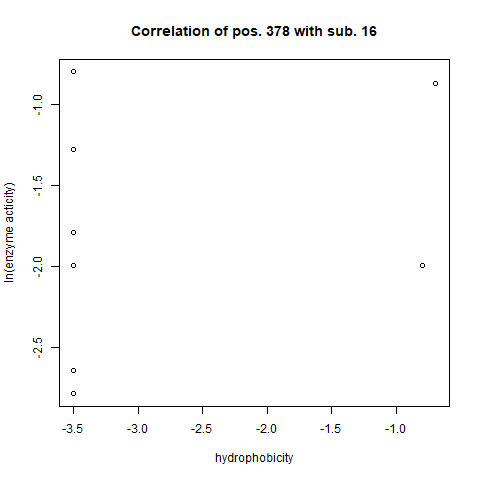

Supplement: Supplementary file 6 — Supplementary Data 3 [file 42004_2024_1207_MOESM6_ESM.zip › Supplementary Data 3/plots/hydrophobicity - 378 - 16 .png]

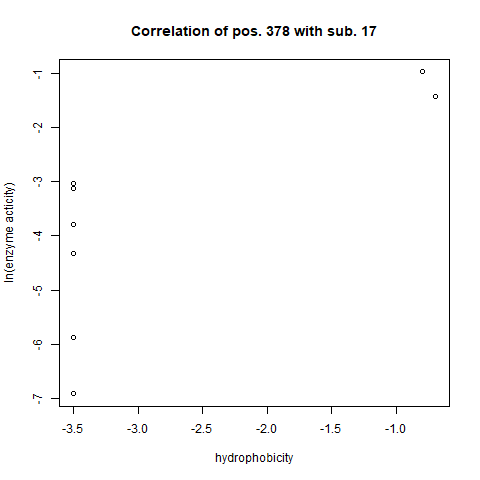

Supplement: Supplementary file 6 — Supplementary Data 3 [file 42004_2024_1207_MOESM6_ESM.zip › Supplementary Data 3/plots/hydrophobicity - 378 - 17 .png]

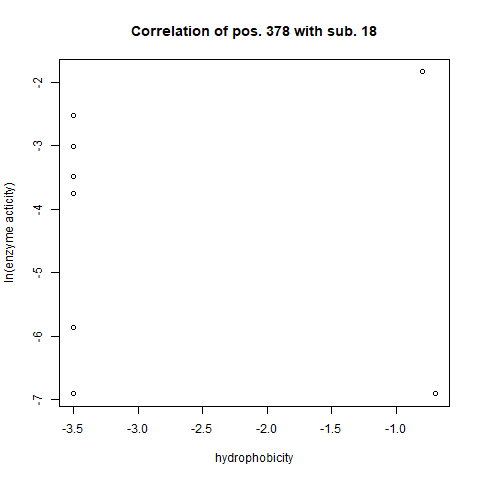

Supplement: Supplementary file 6 — Supplementary Data 3 [file 42004_2024_1207_MOESM6_ESM.zip › Supplementary Data 3/plots/hydrophobicity - 378 - 18 .png]

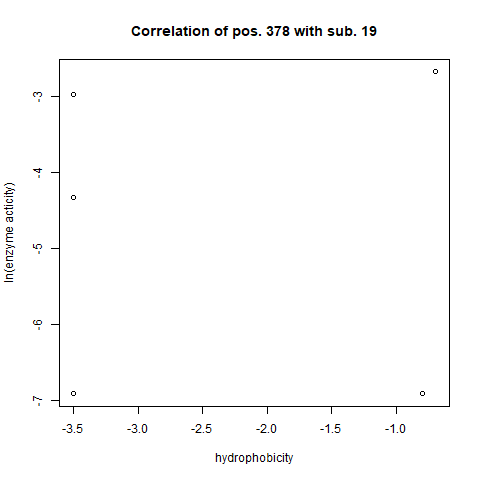

Supplement: Supplementary file 6 — Supplementary Data 3 [file 42004_2024_1207_MOESM6_ESM.zip › Supplementary Data 3/plots/hydrophobicity - 378 - 19 .png]

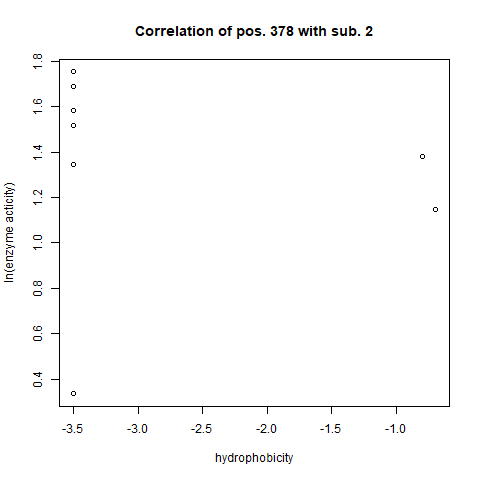

Supplement: Supplementary file 6 — Supplementary Data 3 [file 42004_2024_1207_MOESM6_ESM.zip › Supplementary Data 3/plots/hydrophobicity - 378 - 2 .png]

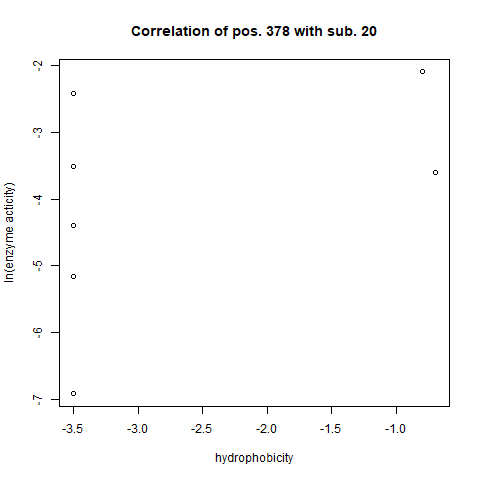

Supplement: Supplementary file 6 — Supplementary Data 3 [file 42004_2024_1207_MOESM6_ESM.zip › Supplementary Data 3/plots/hydrophobicity - 378 - 20 .png]
